# Adapting the ADAPTE framework for Traditional Chinese Medicine clinical practice guidelines: a methodological study

**DOI:** 10.1186/s13020-026-01323-1

**Published:** 2026-01-20

**Authors:** Xiuli Xie, Yangyang Wang, Heba Hussein, Hui Li, Yasser Sami Amer

**Affiliations:** 1 Research Group of Standardization of Chinese Medicine, The Second Affiliated Hospital of Guangzhou University of Chinese Medicine (Guangdong Provincial Hospital of Chinese Medicine), Guangzhou, 510120 China; 2 State Key Laboratory of Dampness Syndrome of Traditional Chinese Medicine, The Second Affiliated Hospital of Guangzhou University of Chinese Medicine, Guangzhou, 510120 China; 3 Guangdong Provincial Key Laboratory of Chinese Medicine for Prevention and Treatment of Refractory Chronic Diseases, Guangzhou, 510120 China; 4 Department of Oral Medicine and Periodontology, Faculty of Dentistry, Cairo University, Cairo, Egypt; 5 State Key Laboratory of Traditional Chinese Medicine Syndrome/Research Group of Standardization of Chinese Medicine, The Second Affiliated Hospital of Guangzhou University of Chinese Medicine, Guangzhou, 510120 China; 6 Pediatrics Department & Clinical Practice Guidelines and Quality Research Unit, Corporate Quality Management Department, King Saud University Medical City, Riyadh, Saudi Arabia; 7 Evidence-Based Health Care and Knowledge Translation, Deanship of Scientific Research, King Saud University, Riyadh, Saudi Arabia; 8 Department of Internal Medicine, Ribeirão Preto Medical School, University of São Paulo (FMRP-USP), Ribeirão Preto, São Paulo Brazil; 9 Adaptation Working Group, Guidelines International Network, Perth, Scotland, UK

**Keywords:** ADAPTE, Adjust and expand, Guidelines, Adaptation

## Abstract

Clinical practice guideline adaptation is critical for standardizing care, yet existing frameworks like the ADAPTE process lack specificity for Traditional Chinese Medicine (TCM), whose principles diverge fundamentally from western medicine. To address this gap, we modified the ADAPTE process to align with the unique characteristics of TCM. A dual-tiered working group (core team and expert panel) simulated tested the original ADAPTE process on Traditional Chinese Medicine clinical practice guidelines (TCM CPGs), identifying challenges such as incompatibility with TCM diagnostic frameworks and holistic treatment paradigms and formed the initial version of ADAPTE-TCM. 21 experts were invited to evaluate the initial version of ADAPTE-TCM and reached a high consensus, while also providing suggestions for modifications to the initial version. Ultimately, 20 steps were modified (e.g., evidence synthesis, consensus-building) and integration of TCM CPGs specific evaluation criteria and tool. The resulting ADAPTE-TCM framework provides methodological rigor for adapting TCM CPGs. This adaptation not only bridges a critical gap in TCM CPGs adaptation but also exemplifies how evidence-based methodologies can evolve to respect diverse medical traditions.

**Supplementary Information:**

The online version contains supplementary material available at 10.1186/s13020-026-01323-1.

## Introduction

For a long time, Chinese medicine and western medicine have complemented each other in promoting the health of the Chinese people. TCM is a major highlight of China's distinctive medical and health services, especially during the COVID-19 pandemic, when TCM played an important role in epidemic prevention and control. In recent years, with the Chinese government's vigorous promotion of TCM, the development of TCM at home and abroad has shown a booming trend, which has not only been comprehensively emphasized and promoted in China, but also gradually expanded its influence internationally. Domestically, medical institutions such as TCM hospitals and clinics have been widely set up at the grassroots level in townships and other areas, and appropriate technologies of TCM, such as acupuncture and Tuina, have been widely promoted [1]; Internationally, with the widespread dissemination and practice of TCM around the world, its unique therapeutic concepts and methods have attracted extensive attention from the international community [2]. The advantages of TCM in improving efficacy, reducing side effects, and promoting natural healing have been gradually recognized and accepted worldwide.

Clinical practice guidelines (CPGs) are the core technical standards in the field of medicine [3], and with the comprehensive development of TCM, TCM CPGs continue to emerge. However, the development and updating of high-quality CPGs requires a large number of resources, and most organizations are under pressure to produce more guidelines in a shorter time with increasingly limited resources. In order to make use of existing guidelines and reduce the duplication of effort, guideline adaptation has been proposed as an option for guideline development [4, 5], and how to make use of the existing clinical guidelines on TCM, and how to provide a systematic approach to adapting guidelines produced in different settings for use in a different cultural and organizational context have become another urgent need for the promotion and application of TCM.

At present, the internationally recognized guideline adaptation tools are all developed for western medical clinical guidelines. If the clinical guidelines adaptation tools of western medicine are directly applied for TCM guideline adaptation, there may be problems that the adaptation tools fail to fully consider the characteristics of TCM and fail to cover the unique contents of Chinese medicine and the special requirements for the context of use, which may lead to the loss of information, misinterpretation or inapplicability. Currently, among the internationally used guideline adaptation tools, the ADAPTE process is particularly the most widely used, mature and well developed [6, 7]. ADAPTE Manual and Resource Toolkit is a systematic guideline adaptation toolkit designed to help decision makers consider factors related to health care and develop clinical guidelines that are appropriate for local use. The process makes guideline adaptation more efficient and transparent through a clear set of steps. The project members of this study are members of the ADAPTE working group. They possess a deeper understanding of ADAPTE and have accumulated extensive application experience through years of practical implementation. The project members have found that there are certain in applications in applying ADAPTE directly to adapting TCM guidelines, and that the ADAPTE process does not take into account the specific content of TCM as well as some of the special needs in developing and implementing TCM guidelines. In order to make the ADAPTE process better applicable to the adaptation of TCM guidelines, we attempted to adjust and expand the ADAPTE process based on the characteristics of TCM in this study.

## Methods

### Establishing research working group

Firstly, we established a research working group for this study. It consisted of: (1) A core working group, including professionals in guideline methodology, clinicians, and statisticians who were responsible for applying the ADAPTE process for hands-on practice, proposing procedures and methodological recommendations applicable to TCM guideline adaptation; analyzing and summarizing the opinions of the Expert Consensus Group, and forming the final version of ADAPTE-TCM [2]. An expert consensus group, including guideline methodology experts, evidence-based medicine experts. The expert consensus group members were selected to ensure representation across geographical regions, TCM disciplines, and gender diversity. The group was responsible for evaluating and proposing modifications on the initial version of ADAPTE-TCM.

### Hands-on practice of ADAPTE-TCM guideline adaptation

To explore the application of ADAPTE in adaptation of TCM CPGs and to propose optimization suggestions based on the characteristics of TCM, we applied the ADAPTE tool to conduct a systematic simulation adaptation of TCM CPGs.

A core operational team was formed, consisting of two researchers (XXL and WYY) with extensive experience in guideline methodology. Both researchers had backgrounds in participating in the development of CPGs and possessed a deep understanding of TCM. The core team conducted a systematic and immersive study of the ADAPTE manual (Version 2.0). Through focused workshops and case analysis, both members ensured a comprehensive familiarity and understanding of the theoretical concepts and operational details of all 24 steps across the three phases (preparation, adaptation, and completion) of the ADAPTE process.

Two highly rated guidelines were selected using "Study on Quality Assessment of TCM Clinical Practice Guidelines Based on AGREE Instrument" for adapting operations [8]. They are *CPGs of Chinese Medicine for Migraine Headache* [9] and *CPGs of Chinese Medicine for Primary Osteoporosis* [10], which were funded by the WHO Western Pacific Region Fund and developed by the Chinese Academy of Traditional Chinese Medicine (CAMC), respectively.

The two researchers strictly followed the 24 steps of the ADAPTE framework, independently and concurrently performing a complete simulated adaptation for both cases. The key steps include: (1) In the preparation phase, first clarify the scope of the guidelines and the target users, and simulate the establishment of a multidisciplinary guideline group. During this process, focus on the TCM background of the personnel in the multidisciplinary guideline group, the source databases for TCM guidelines, and the precautions for selecting TCM guidelines. (2) In the adaptation phase, the main focus is on the applicable tools for TCM guideline adaptation and the unique content of TCM, such as syndrome differentiation and treatment, TCM evidence (such as classical literature and experiences of renowned TCM doctors), and the contextual applicability of TCM interventions, which pose challenges to guideline adaptation. In the finalization phase, the main attention is given to reporting standard tools applicable to TCM CPGs.

Throughout the simulated application, both researchers maintained detailed research logs, recording issues encountered at each step that were related to the unique characteristics of TCM. Following the practical application, the two researchers first reflected independently and then held a joint discussion. Each researcher, based on their practical experience, generated specific supplementary or modificative suggestions corresponding to each of the 24 ADAPTE steps. Subsequently, the core working group conducted a detailed line-by-line review of all modifications and additions proposed by the two researchers. Following this comprehensive discussion, the group integrated the approved changes to develop an initial version of the ADAPTE-TCM framework.

### Expert consensus

The modified Delphi method is applied, consisting of one or two rounds of expert investigation in the form of electronic questionnaire. Experts were invited to evaluate the 24 steps of the initial version of ADAPTE-TCM. When experts’ opinions reached convergence after the first round of the survey, the second round of investigation was skipped. Before the survey, all experts were informed of the purpose of this study and were invited to sign the informed consent form. We asked experts to score the importance of each item. For the evaluation index, we adopted a three-scale scoring method, which were “agree” “somewhat agree” and “disagree”, assigned three, two and one points, respectively.

When experts did not fully agree with certain content of the steps, they were invited to offer specific modification suggestions. Next, questionnaires were collected and sorted. The team members optimized the evaluation steps' content according to the results and drafted the final version of ADAPTE-TCM based on the expert consensus reached.

### Statistical analysis

We evaluated expert consensus using the degree of expert positivity and authority [3, 11] Expert positivity coefficient was the recovery rate of valid questionnaires. The principles for judging a questionnaire were as follows: (1) Unqualified answers accounted for a large proportion of the questionnaire. It's invalid if all the evaluated steps get full marks, for instance. (2) Missing responses to key variables in a survey topped 15%

Expert authority coefficient (Cr) is the basis for experts to make judgments on problems, represented by Ca, and the coefficient of experts' familiarity with indicators, represented by Cs. The sum of judgment coefficient and familiarity is equal to authority, that is [3, 12]:

$${\mathrm{Cr}} = \left( {{\mathrm{Ca}} + {\mathrm{Cs}}} \right)/{2}$$

Experts’ credibility hinges heavily on their own assessment. Expertise is linked to prediction precision, which grows with the former. Four dimensions of evaluation are theoretical analysis, practical experience, knowledge from peers, and intuition. Each dimension is divided into three grades: high, medium and low, according to the degree of impact it has on expert decision-making. The values are as indicated [3, 12]: theoretical analysis (0.3, 0.2, 0.1), practical experience (0.5, 0.4, 0.3), cognition from different perspectives (0.1, 0.1, 0.1) and intuition (0.1, 0.1, 0.1, 0.1). The degree of familiarity can be separated into five classes: 1, 0.8, 0.6, 0.4, and 0.2 [12].

Statistical analysis was conducted in Statistical Package for the Social Sciences (SPSS) 17.0. The concentration degree for experts’ opinions was represented by arithmetic mean, grade sum and full score rate; coordination degree for experts' opinions was represented by a variation coefficient and a coordination coefficient.

## Result

### Forming the initial version

The first draft of the extended version of the ADAPTE tool for TCM CPGs was drafted based on the modification suggestions of two evaluators, and after discussion at the core group meeting, adjustments and additions were proposed to the ADAPTE tool for Steps 1–4, 6–7, 10–17, 19–20, 22 and 24, a total of 18 steps. The content and rationale for the proposed changes are shown in Table 2.

(1) Step 1, additions to websites for TCM/integrated Chinese and western medicine (ICWM) guideline sources were presented in tool 1. And the addition of the search sources and strategies were provided in Tool 2. (2) Step 2, addition of references to the information provided when the guidelines were formed into an adapted group: *Manual for the Development of Integrated Chinese and Western Medicine Treatment Guidelines* [13]. (3) Step 3, in identifying areas for best practice guideline adaptation and their prioritization, two additional criteria were added: Criterion 1, demand for TCM guidelines based on the regions where they are applied. Criterion 2, the existence of relevant high-quality evidence-based guidelines on TCM. (4) Step 4, ensuring resources needed for guideline adaptation, suggests adding specialized skills in TCM clinical practice, guideline methodology and information retrieval. (5) Step 6, demonstration of switching to TCM diagnosis and treatment of psoriasis vulgaris in the example section (6) Step 7, switching to TCM for psoriasis vulgaris raises PIPOH questions in Tool 6. (7) Step 10, suggesting the utilization of the Chinese medicine guidelines research and evaluation tool (AGREE II for TCM) tool for screening of TCM guidelines. (8) Step 11, it is recommended that the quality of TCM guidelines be evaluated using AGREE II for TCM, and it is recommended that the quality evaluation tool for TCM evidence-based diagnosis be applied to evaluate the quality of the evidence-based diagnostic part of the TCM guidelines [3]. (9) Step 12, when evaluating the timeliness of TCM guidelines, it is recommended that attention should be paid to the time span of evidence in the guidelines and analyze whether ancient evidence is included. (10) Step 13, when conducting and evaluating the summary of guideline content, it is recommended that the summary form be filled out or reviewed by a TCM clinician who specializes in the topics. Revise the sample in Tool 12 into a comparative summary table of clinical recommendations for the blood-heat syndrome, as outlined in *TCM diagnosis and treatment guidelines for psoriasis vulgaris* and *Integrated traditional Chinese and western medicine diagnosis and treatment guidelines for psoriasis vulgaris*. (11) Step 14, when evaluating guideline consistency, it is recommended that it should be conducted by both TCM clinicians and methodologies. (12) Step 15, when evaluating the acceptability or feasibility of the recommendations, it is suggested that a particular consideration for TCM guidelines is the acceptance of local culture and policy for TCM, Chinese herbal, and invasive procedures such as acupuncture; it is recommended that the recommendations be implemented about the *Manual for the development of integrated Chinese and western medicine treatment guidelines* [13]. (13) Step 16, review assessments, add the results of the assessment of the research and evaluation tool for guidelines in AGREE II for TCM and the guideline reporting specification RIGHT TCM extended edition (RIGHT-TCM) mentioned in the previous steps to the review summary information. (14) Step 17, When formulating recommendations, emphasis should be placed on how to translate complex interventions based on “syndrome differentiation and treatment” (such as rheumatic fever accumulation syndrome, blood deficiency and wind-dryness syndrome) into clear recommendations that healthcare practitioners in the target country or region can understand and patients can accept, while fully considering the locally available herbal products and acupuncture services. (15) Step 19, external review, suggests referring to chapter 11 of the *Manual for the development of integrated Chinese and western medicine treatment guidelines* for implementation [13]. (16) Step 20, before the guideline is released, it is recommended that it be formally endorsed by the local TCM administration or TCM societies/associations or alliances before release. (17) Step 24, it is recommended that the final guideline be reviewed using the AGREE II for TCM instrument as well as RIGHT-TCM instrument as a checklist. Also, the examples in Steps 13, and 22 are changed to examples of TCM guidelines.

### Expert consensus

The main work of the expert consensus panel is to reach consensus on the first draft of the ADAPTE Guidelines on Chinese medicine adaptation and expansion. There were 21 members in the expert consensus panel, who are TCM and evidence-based medicine experts from different regions, and they are representative in terms of geography, gender and specialty, with specialties in the fields of TCM, evidence-based medicine, guideline development methodology, clinical pathway research, etc. The composition of the expert group is shown in Supplementary file 1. The gender distribution of the experts was 9 males (accounting for 42.9%) and the gender distribution of experts was 9 males (accounting for 42.9%) and 12 females (accounting for 57.1%). The age of the experts was 26–44 years old, with an average of 34.2 ± 4.0 years, and the number of years of research in related fields was 3–15 years, with an average of 8.3 ± 3.36 years.

Twenty-two experts were invited, 21 questionnaires were recovered, the recovery rate of the questionnaires was 95.5%, the invalid questionnaires were 0, and the positive coefficient of experts was 95.5%. It is generally considered that the expert authority coefficient (≥ 0.70) is an acceptable value [12]. The authority coefficient of experts was 0.81, which indicates that the experts had a high degree of authority over the content of the consensus.

Expert opinion concentration degree observation indicators include arithmetic mean, full score ratio, rank and the meaning of the three indicators are as follows: the arithmetic mean is the average number of evaluation index scores, full score ratio (Ki) refers to the number of experts in the total number of experts who evaluate the content of a certain step as “agree”, and the rank sum (Si) represents the total score of experts on the degree of approval of a step. If the mean score of an item is ≤ 2, or when Ki ≤ 30%, or Si ≤ 44 (3 * 21 * 70%), it means that the content of the step is unreasonable, and should be revised according to the experts' opinions and re-considered.

The results of the first round of expert survey indicate that all steps are highly recognized by experts. The arithmetic mean of all entries is ≥ 2.6, the full score ratio is ≥ 66.7%, and the rank sum is ≥ 56 (Table 1).

**Table 1:** Results of observation indicators on the degree of concentration and harmonization of expert opinions

Evaluation entry	N	Mean	Full score ratio (%)	Rank sum	Coefficient of variation
Step1	21	2.8	76.2	58	16%
Step2	21	2.7	71.4	56	22%
Step3	21	2.7	76.2	57	21%
Step4	21	2.8	81	58	20%
Step5	21	3.0	95.2	62	7%
Step6	21	2.9	90.5	61	10%
Step7	21	2.9	90.5	61	10%
Step8	21	2.9	90.5	61	10%
Step9	21	2.9	90.5	61	10%
Step10	21	2.7	81	57	24%
Step11	21	2.9	90.5	61	10%
Step12	21	2.7	66.7	56	18%
Step13	21	2.9	90.5	61	10%
Step14	21	2.8	81	59	14%
Step15	21	2.9	90.5	61	10%
Step16	21	2.8	76.2	58	16%
Step17	21	2.9	90.5	61	10%
Step18	21	2.9	90.5	61	10%
Step19	21	2.9	90.5	60	13%
Step20	21	2.8	85.7	59	18%
Step21	21	2.9	90.5	60	17%
Step22	21	2.9	90.5	61	10%
Step23	21	3.0	100	63	0
Step24	21	3.0	95.2	62	7%

The degree of fluctuation, or coordination, of the experts’ evaluation of a step was analyzed using the coefficient of variation (CVi). The smaller the CVi, the higher the degree of coordination of the experts' evaluation of a step. Currently, it is generally accepted that a CVi of less than 25% or 30% is considered to have low variability [13, 14]. The survey results indicate that the coefficient of variation of experts’ opinions on each step fluctuates between 0 and 0.24, with a small coefficient of variation and a high degree of consistency. The details are shown in Table 1.

### Formation of the extended version of the ADAPTE TCM CPGs adaptation tool

After the first round of the survey, all steps in the tool list received a high degree of consensus and expert authority from the experts. The arithmetic mean of all steps was ≥ 2.6, the full score ratio was ≥ 66.7%, the rank sum was ≥ 56, and the coefficient of variation was small, fluctuating between 0 and 0.24, with a high degree of consistency. A high mean score means that the experts recognized the importance of the step, and a low coefficient of variation means that the evaluation results of all the steps were highly consistent across experts, and a subsequent second round of the survey could not be conducted.

Some experts suggested linguistic changes to some steps or recommended the addition of certain tools to manipulate the content, with a total of 88 written suggestions from 15 experts for the 24 adaptation steps. After discussion by the project team, it was decided to adopt the experts' opinions to supplement and improve the contents of some steps without affecting the core contents of the entries. Finally, under the guidance of the overall framework of ADAPT, the ADAPTE-TCM was formed with Chinese medicine characteristics.

The structure of ADAPTE and the main content of all steps were adopted in ADAPTE-TCM, including three major phases (preparation, adaptation, and completion), totaling 24 steps. After being modified and supplemented, the step content was applicable to TCM CPGs; when listing examples of operations in each step, try to refer to examples from TCM CPGs; the relevant tools used in each step should also be as applicable as possible to TCM CPGs, such as AGREE II for TCM RIGHT-TCM, etc. After discussion, the working group adopted the opinions of the experts, and made the recommendations for steps 2–4, 6, 8, 14–16, 18–20 and 24, including the structure of ADAPT and the main contents of all the steps.

Finally, based on the modification suggestions from the two reviewers and the survey results from 21 experts, steps 1–4, 6–8, 10–20, 22, and 24 were revised and improved, forming an ADAPTE extension version for TCM CPGs. The comparison between ADAPTE and ADAPTE-TCM is showed in Table 2. ADAPTE-TCM is showed in Supplementary file 2.

**Table 2:** Comparison Table: Original ADAPTE vs. ADAPTE-TCM

Step number	Original ADAPTE component	ADAPTE-TCM	Rationale or justification
Step 1	Tool 1 and Tool 2	Additions to websites for TCM/Integrated Chinese and Western medicine guideline sources in tool 1. and the addition of the search sources and strategies provided in Tool 2	Provided the necessary guideline development resource tools and database resources for the adaptation of TCM CPGs
Step 2	For the remainder of the document, the term 'panel' will refer to the multidisciplinary group convened for the tasks of the adaptation process Members of the organizing committee may also be panel members or may solely act to set the process in place	Additions of references to the information provided when the guideline is formed into an adapted group: *Manual for the Development of Integrated Chinese and Western Medicine Treatment Guidelines*	The overall process of guideline adaptation and the division of labor among the staff are similar to the process of guideline development, and it is more reasonable to set up a guideline adaptation committee by referring to the method of constructing and managing a project team for TCM CPGs, which is commonly implemented
Step 3	There are a number of criteria that can be used to identify and prioritize areas for best practice and guideline adaptation For example, these criteria might include: • The prevalence of the condition • The existence of underuse, overuse, or misuse of interventions • The burden associated with the condition (e.g., a system, financial, or patient burden) • The existence of relevant good-quality evidence-based guidelines	Additional elaboration on criteria 1, 2, 3, 8, for example, Criterion 2, demand for TCM CPGs based on areas of guideline application and Criterion 8, the existence of relevant high-quality evidence-based guidelines on TCM	Clarification of the burden of disease, assessment of the use of interventions, and the region of application of the guidelines, as well as improving the relevance of TCM CPGs
Step 4	The following skills should be represented on the panel: • Clinical knowledge in the topic area—knowledge of the issues related to the application of the guideline in local practice and of the latest research in the topic area • Methodological expertise (e.g., health services researchers)—knowledge of research design and knowledge in critical appraisal and guideline appraisal play a role in educating other panel members on issues related to the systematic and rigorous nature of the process and provides a methods resource • A multidisciplinary group is important if the guideline addresses issues that impact several provider groups. The involvement of a mix of disciplines ensures that issues such as those related to the application of the guideline, to the evidence behind the recommendations, and to the impact on patients will all be considered	Ensuring resources needed for guideline adaptation, suggests adding specialized skills in TCM clinical, guideline methodology and information retrieval. The proposed increase in the number of health economic analysts in the area of methodological expertise is described	Clarify that the panel members should have professionals related to TCM or Integrated Traditional Chinese and Western Medicine, which is more directional; According to the current method of guideline development, add health economic analysts to ensure the implementation of the health economic evaluation of the guideline adaptation p
Step 6	• Throughout the process, each decision taken by the organizing committee and the multidisciplinary panel should be well documented to make the process transparent. A person needs to be identified to manage and communicate this plan to all panel members • Illustration—Set Up Phase	• Additional recommendations that the guideline planner be registered if necessary • Demonstration of switching to TCM diagnosis and treatment of psoriasis vulgaris in the example section	• Registering a medical guideline development plan helps prevent redundant efforts by different teams, optimizing resource allocation, while enabling centralized quality oversight and global collaboration to ensure guideline timeliness and standardized evidence-based medicine advancement • It is more relevant to use the TCM CPGs as an illustrative example
Step 7	Tool 6—PIPOH	Switching to TCM for psoriasis vulgaris raises PIPOH questions in Tool 6	It is more relevant to use the Chinese Medicine Guidelines as an illustrative example
Step 8	A MEDLINE (www.ncbi.nlm.nih.gov/entrez/query.fcgi) search using a standardized search strategy may yield additional guideline	Suggested search of literature databases, supplemented with Chinese databases	More TCM CPGs are published in Chinese databases than in foreign language databases
Step 10,	Tool 9—AGREE Instrument	Suggesting the utilization of the Chinese Medicine Guidelines Research and Evaluation Tool (AGREE II for TCM) tool for screening of TCM guidelines	AGREE II for TCM instrument was developed based on the characteristics of the TCM CPGs and is suitable for the assessment of methodological quality of TCM CPGs
Step 11	The AGREE instrument The appraisal of guidelines research & evaluation (AGREE) Instrument (www.agreetrust.org) provides a framework for assessing the quality of clinical practice guidelines	• It is recommended that the quality of TCM CPGs be evaluated using the Research and Evaluation Tool for TCM CPGs (AGREE II for TCM), and it is recommended that the quality evaluation tool for TCM Evidence-based diagnosis be applied to evaluate the quality of the evidence-based diagnostic part of the TCM CPGs • Addition of Tool 10–Quality evaluation of TCM diagnosis based on syndrome differentiation	AGREE II for TCM instrument was developed based on the characteristics of the TCM CPGs and is suitable for the assessment of methodological quality of TCM CPGs • Syndrome differentiation and treatment are the core of TCM, and TCM syndrome differentiation and diagnosis are the characteristic components of TCM CPGs. Providing quality assessment tools for TCM syndrome differentiation and diagnosis can provide reference for the overall quality assessment of guidelines
Step 12	Assess guideline currency	When evaluating the timeliness of TCM CPGs, it is recommended that attention should be paid to the time span of evidence in the guidelines and analyze whether ancient evidence is included	Chinese medical antiquarian evidence has a significant impact on the formation of guideline recommendations. The inclusion of evidence from ancient books ensures the systematic and holistic nature of evidence in TCM CPGs
Step 13	• Step 13. Assess guideline content • Matrices are tables of recommendations drawn from the guidelines under review, although they also might include recommendations from systematic reviews or health technology assessments. We recommend that a clinician who specializes in the topic produce or review the matrices to ensure that no recommendation has been taken out of context • Tool 12-Sample Recommendations Matrices	• Replace the title of step 13 with the following: Assess the recommendations of guideline • When conducting and evaluating the summary of guideline content, it is recommended that the summary form be filled out or reviewed by a TCM clinician who specializes in the topic • Revise the sample in Tool 12 into a comparative summary table of clinical recommendations for the blood-heat syndrome, as outlined in TCM Diagnosis and Treatment Guidelines for Psoriasis Vulgaris and Integrated Traditional Chinese and Western Medicine Diagnosis and Treatment Guidelines for Psoriasis Vulgaris	• This step focuses on the Mission's assessment of the recommendations, which is a more appropriate name for this step • Completion or review of the matrices by a TCM or Integrative Medicine clinician is more professional • Providing examples of TCM CPGs is more exemplary and instructive
Step 14	The assessment of the consistency of the guideline includes the following three evaluations: • Search strategy and selection of evidence supporting the recommendations • Consistency between the selected evidence and how developers summarize and interpret this evidence • Consistency between the interpretation of the evidence and the recommendations	• The following was added to the guideline's consistency assessment: the correspondence between the quality of evidence and the strength of the recommendation (in particular, whether there is a sufficiently persuasive rationale when low-quality evidence gives a strongly recommended opinion) • When evaluating guideline consistency, it is recommended that it should be conducted by both TCM clinicians and methodologies	• The correspondence between the quality of the evidence and the strength of the recommendation is an important element of consistency assessment and a reflection of the rigor of guideline development • Clinical staff or methodologists with a background in TCM are more specialized in completing the consistency assessment
Step 15	Assessing whether a recommendation is acceptable and/or applicable or not is done by discussing each recommendation in light of the following questions	• When evaluating the acceptability or feasibility of the recommendations, it is suggested that a particular consideration for TCM CPGs is the acceptance of local culture and policy for TCM, Chinese medicine, and invasive procedures such as acupuncture • It is recommended that the applicability of these guidelines be assessed using the following resources: the AGREE-REX instrument, the Guangdong Provincial Local Standard Evaluation Guidelines for Traditional Chinese Medicine Diagnosis and Treatment, and the Manual for Developing Integrated Traditional Chinese and Western Medicine Clinical Practice Guidelines	• The implementation of TCM CPGs has a close correlation with local health policies, and emphasis should be placed on the acceptance of TCM in local health policies • Recommended assessment tools applicable to TCM CPGs for assessment
Step 16	Review assessments	Integrate the evaluation results of all TCM CPGs assessment tools mentioned in the previous steps into the review summary information	Supplement the evaluation results of all assessment tools for TCM CPGs
Step 17	Caution	Focus on how to transform complex interventions based on "syndrome differentiation and treatment" (such as rheumatic fever accumulation syndrome, blood deficiency and wind-dryness syndrome) into clear recommendations that healthcare practitioners in the target country or region can understand and patients can accept, while fully considering the locally available herbal products and acupuncture services	There may be issues of understanding and different service condition limitations regarding Traditional Chinese Medicine interventions in different regions
Step 18	Once the panel has reached a decision on the content of the adapted guideline, a draft document will be produced that should include details on the process followed. A suggested template for the format of the guideline is presented in Tool 16	It is recommended that the source guideline developer be contacted for additional guideline-related information It is suggested that reference can be made to the RIGHT-TCM tool and RIGHT-Ad@pt lists for guideline drafting	Many of the guideline development process documents are not publicly available, and contacting the source guideline developer for more guideline-related information can help in the adaptation process Followed closely the research results of guideline development and adaptation, and supplemented the latest reporting standards applicable to Chinese medicine guidelines or guideline adaptation
Step 19	A structured questionnaire is helpful for this step	It is recommended to refer to DB/T44 2218.6–2019 *General Rules for the Formulation and Revision of Clinical Practice Guidelines for Traditional Chinese Medicine (Combined Traditional Chinese and Western Medicine) External Review* or Chapter 11 of *Manual for the Formulation of Guidelines for Combined Traditional Chinese and Western Medicine Diagnosis and Treatment*	The recommended standards and book had established a set of external review procedures and methods for TCM CPGs that can be directly adopted
Step 20	In order to help with widespread implementation, we recommend that the adapted guideline be formally endorsed by professional body(ies) or organization(s) most closely connected to the guideline topic (e.g., a national college of family physicians might endorse guidelines related to primary care).. The endorsement of a guideline by relevant professional organizations has been shown to enhance the acceptability of a guideline to the organization’s members	Before the guideline is released, it is recommended that it be formally endorsed by the local TCM administration or TCM societies/associations or alliances before release	Taking into account the procedures for publishing local Chinese medicine guidelines in China, it is additionally recommended to apply for accreditation at the local medical management department or Chinese medicine (integrated Chinese and Western medicine) societies/associations or alliances, and considering that the guidelines can be published as standards, it is also recommended to follow the relevant procedures for the establishment of the standards and their publication
Step 22	Illustration—Process of external review of the cervical cancer screening guideline	Replace the illustration with the process of external review of the Chinese medicine diagnosis and treatment of psoriasis vulgaris guideline	The example of changing to TCM CPGs is more exemplary
Step 24	The final product might be reviewed using the AGREE instrument (6) as a checklist to assess how the adapted guideline rates with respect to quality criteria	It is recommended that the final guideline be reviewed using the AGREE for TCM instrument or RIGHT-TCM instrument	AGREE II for TCM instrument and RIGHT-TCM instrument were developed based on the characteristics of the TCM CPGs

### Pilot test plan recommendations

It is recommended to prioritize adapting common disease guidelines that have advantages in Traditional Chinese Medicine diagnosis and treatment, such as eczema and headaches. The basis for selection is that these diseases are relatively easier to gain international recognition and acceptance, which is conducive to conducting stakeholder research in a cross-cultural context. Next, we would take eczema as an example.

Search and select a high-quality TCM CPGs for eczema as the main source guideline. Choose western European countries (such as the UK or Germany) as the target adaptation environment. This can effectively test the ADAPTE-TCM process in handling cultural differences, drug regulation (such as access to TCM decoctions/patented medicines), and differences in ICWM treatment models.

Form a multidisciplinary team consisting of Chinese dermatology experts in TCM, dermatology experts from the target country, general practitioners, methodologists, health policy researchers, and local representatives of patients who have previously used TCM for eczema. The team would strictly follow the 24 steps of ADAPTE-TCM for adaptation.

It is recommended to use a mixed-methods research approach, collecting data from multiple dimensions to assess the ADAPTE-TCM process. The evaluation criteria include quality, feasibility and stakeholder acceptance. Use the internationally recognized AGREE II for TCM tool to evaluate the quality of the finalized pilot guideline. Comparison should also be made with the AGREE II scores of the source guideline or with guidelines in similar fields that have not been systematically adapted to assess the improvement in the quality of the final guideline attributable to the ADAPTE-TCM process. Use a feasibility scale to conduct a questionnaire survey among 30–50 primary healthcare workers (e.g., general practitioners, community nurses) in the target country after the guideline publication. This scale will evaluate aspects such as the clarity of guideline recommendations, compatibility with existing workflows, and accessibility of required resources. The other is qualitative assessment. Conduct semi-structured interviews with some participants and end-users to gain an in-depth understanding of specific barriers (such as the accuracy of terminological translation and clinical applicability of recommendations) and enabling factors encountered during the application of the ADAPTE-TCM process. Stakeholder acceptance contains acceptance by adaptation team and end-user. At the end of the project, conduct a questionnaire survey for all adaptation team members to assess their recognition of the usability, rationale, and relevance of each step in the ADAPTE-TCM process. Through a survey targeting medical practitioners in the target country, measure their willingness to adopt guideline recommendations.

Through the above tests and evaluations, it is expected to obtain preliminary empirical data on the effectiveness of the ADAPTE-TCM process, identify aspects requiring further optimization, and provide a solid and reliable methodological tool for the international dissemination and local application of TCM guidelines.

## Discussion

In this study, we adapted and extended the ADAPTE tool for TCM CPGs. Based on the overall ADAPTE framework, a tool for TCM CPG adaptation was constructed. This study adopted a multidisciplinary and collaborative approach, utilizing action research methods, expert questionnaires, mathematical and statistical methods, and core group discussion qualitative research.

In the first part of the study, the action research method was used. Two researchers from the core working group used the ADAPTE tool to conduct a simulated adaptation of TCM CPGs. The purpose of the simulated adaptation is to understand the applicability of the ADAPTE tool to the TCM CPGs, and then to propose additions or modifications to the TCM CPGs. This approach combines practical action with scientific research, emphasizing the identification and resolution of problems in practical work. During the hands-on process, project team members were able to visualize the strengths and weaknesses of the tool, and thus suggest specific modifications. This feedback based on practical experience is of great significance in guiding the subsequent adaptation work. The expert questionnaire survey was conducted by experts from many regions in the east, west, south, and north of China. All experts were familiar with the field of TCM CPGs research. The experts' authority score was 0.81, indicating that the surveyed experts have a certain degree of authority in the field of TCM CPGs adaptation. The experts made a total of 88 suggestions for modifications to the 24 steps, and the project team, after thorough discussion, integrated the experts' opinions to supplement and improve each step, forming the expanded version of the ADAPTE for TCM CPGs. The non-stop improvement of the above research process ensures the scientificity, rationality and applicability of the tool.

There are three main types of adjustments and adaptations to ADAPTE. The first type is to highlight the characteristics of TCM, such as in step 12, when assessing the timeliness of the guidelines, full consideration should be given to the specificity of TCM, as historical TCM evidence significantly impacts guideline recommendation formulation. In determining the timeliness of the evidence supporting the guideline, the time span of the evidence in the TCM CPGs should be determined based on the overall publication and updating characteristics of TCM CPGs in this field, and the guideline should be reviewed to see if it includes antiquarian evidence and whether the relevant TCM antiquarian evidence has been systematically traced back to the beginning of the documented history of a particular disease. The inclusion of antiquarian evidence ensures the systematic and holistic nature of the evidence in the TCM CPGs; Steps 15 and 17, when evaluating the acceptability or feasibility of the recommendations, special consideration should be given to the local cultural and policy acceptance of TCM, Chinese medicine, and invasive procedures such as acupuncture, and the coverage of TCM-related therapeutic techniques by the local healthcare insurance system. Different parts of the world have different levels of acceptance of TCM due to differences in cultural backgrounds, healthcare systems and legislative processes. Different countries and regions have different laws and regulations on the use of TCM, which have a bearing on whether the TCM CPGs can be promoted and implemented well, and therefore the evaluation of the feasibility of the recommendations should focus on the local situation in this regard. Steps 13 and 14, it is recommended that the relevant procedures be operated by clinical and methodological experts in TCM, and that the accuracy of the evaluation or collection of content be ensured by personnel familiar with the field. Step 20, the original ADAPT tool suggests that the guidelines be adapted to form a draft and then consult the relevant professional bodies for certification. Considering the local characteristics of China, in the extended version, in addition to applying for certification by professional bodies, it is added that the project can be set up and released in the form of standardization projects. In China, the standards are divided into national standards, industry standards, local standards and group standards, and the adapted guidelines can be applied to all levels according to their scope of application. Standard management part of the application project, after the establishment of the project in accordance with the standard-setting procedures and ultimately released as a guide to the release of the standard can effectively improve the acceptance of the implementation of the promotion and wide degree.

The second category is the addition of resources and tools applicable to the adaptation of TCM CPGs. In Step 1, Tool 2 was supplemented with the addition of websites related to the sources of TCM/ICWM CPGs, such as the official websites of TCM-related societies, such as the Chinese Society of Traditional Chinese Medicine and the World Federation of Chinese Medicine Societies, as well as commonly used Chinese journal databases, such as China Knowledge and Wanfang full-text databases. Step 2 establishment of a guideline panel, step 15 evaluation of the feasibility of the recommendations, and Step 19 external review of the guideline, it is recommended to refer to the relevant standards and monographs on the development and revision of TCM/ICWM CPGs, which have fully considered the characteristics of TCM/ICWM CPGs. These process guidelines are comprehensive and scientific, serving as authoritative reference materials for formulating TCM/ICWM CPGs.. Steps 10, 11, and 24 respectively recommend the use of the AGREE II for TCM tool for screening, quality evaluation, and final review of guidelines as a checklist for TCM CPGs. This tool, published in 2023, is an expanded version of AGREE II for TCM CPGs, which retains the scientific and rigorous nature of the AGREE II guideline evaluation tool, while highlighting the characteristics of TCM and the tool retains the scientific and rigorous nature of the AGREE II evaluation tool, highlights the characteristics of TCM, and corrects the deficiencies of AGREE II, which is more suitable for the quality evaluation of TCM CPGs. In step 11, it is recommended to apply the quality evaluation of TCM diagnosis based on syndrome differentiation to evaluate the quality of thediagnostic section of TCM CPGs. The diagnostic section is an important part of TCM/ICWM CPGs, and the rigor of the process of the diagnostic criteria and the accuracy of the criteria will affect the selection of subsequent treatment methods. The rigor of the identification criteria development process and the accuracy of the criteria presentation will affect the subsequent choice of treatment means; therefore, the quality assessment of the identification and diagnosis part of the guideline should be conducted. In step 18 It is recommended to draft an adapted version of the TCM CPGs with reference to RIGHT-TCM, the RIGHT-Ad@pt checklist. In step 24, it is recommended to use the RIGHT-TCM as a checklist for final review of the final guidelines. RIGHT-TCM is a TCM CPGs reporting specification that combines TCM CPGs theory with the RIGHT checklist and aims to provide a clear reporting framework and guidance for TCM CPGs developers. This specification was completed under the guidance of the RIGHT working group, and at the same time combines the characteristics of TCM to fully ensure the standardization, rationality, and comprehensiveness of TCM CPGs reporting. The RIGHT-Ad@pt is a RIGHT working group guideline for the adaptation of guidelines for medical practice. The reporting checklist developed by the RIGHT working group takes into full consideration the characteristics of adapted guidelines and is suitable for guidance on how to draft adapted guidelines.

The third category is an addition to the original content (non-Chinese medicine). In Step 4, the category of health economics analysts has been added to the requirement of personnel with professional knowledge of opposing law. This is because health economics analysts can help the guideline development team to make an evidence-based economic assessment, which ensures that the treatment options recommended by the guidelines are not only clinically effective, but also economically feasible. In step 6, registration of guideline protocols, if necessary, is recommended if there is a relevant local platform for registration of guideline protocols, which helps to ensure guideline quality, enhance transparency, and avoid duplication of efforts. In Step 16, all assessment results used in the guideline adaptation process were integrated, including guideline consistency assessment and feasibility assessment of recommendations, making it more comprehensive than the original version.

Under the guidance of the ADAPTE framework, ADAPTE-TCM tool does not expand the overall framework structure and the adaptation process, but only supplements or modifies the content applicable to TCM because of the original program. It also supplements the relevant tools applicable to TCM CPGs, or supplements the content of the original version (not in TCM) to make it more complete. It retains the scientific and rigorous nature of ADAPTE, an internationally recognized guideline adaptation tool, while highlighting the characteristics of TCM and correcting the deficiencies of ADAPTE. The application of this tool to the adaptation of TCM diagnostic and treatment guidelines can better ensure the methodological quality of the adapted guidelines for TCM. The ADAPTE-TCM is not only suitable for the domestic context, but also provides a foundation for international application for the modular and multi-step characteristics. We recommend that when applying this tool in specific countries or regions in the future, a truly representative local adaptation team should be established, and the evaluation points of each step should be adapted strictly according to the aforementioned dimensions. In this way, it is possible to maintain the scientific rigor of the guidelines while ensuring that they are truly implemented in the diverse policy, cultural, and practice environments outside China, thereby promoting the high-quality and standardized service of TCM for global public health.

## Supplementary Information

Supplementary Material 1.

Supplementary Material 2.

Supplementary Material 3.

## Data Availability

Not applicable.

## Abbreviations

ADAPTE: Adaptation of Guidelines
TCM: Traditional Chinese Medicine
CPGs: Clinical practice guidelines
TCM CPGs: traditional Chinese medicine clinical practice guidelines
COVID-19: Coronavirus Disease 2019
