# Supplementary material for: Adapting the ADAPTE framework for Traditional Chinese Medicine clinical practice guidelines: a methodological study
Source: Chin Med. 2026 Jan 20;21:42. doi: 10.1186/s13020-026-01323-1 (PMC12817658; doi:10.1186/s13020-026-01323-1)
Supplement: Supplementary file 1 — Supplementary Material 1. [file 13020_2026_1323_MOESM1_ESM.docx]

**Composition of the expert group**

| **No.** | **Name** | **Gender** | **Country/region** | **Specialty** | **Title** |
| --- | --- | --- | --- | --- | --- |
|  | Shan liu | female | Zhejiang | Clinical Research Methodology | deputy chief physician |
|  | Luan Zhang | female | Guangdong | TCM Neurology | Resident doctor |
|  | Manjie Zeng | male | Guizhou | Clinical Research Methodology | Assistant Researcher |
|  | Huimin Wang | female | Guangdong | TCM Neurology | Resident doctor |
|  | Long Ge | male | Gansu | Clinical Research Methodology | Professor |
|  | Jianxiong Cai | male | Guangdong | Clinical Research Methodology | Assistant Researcher |
|  | Liang Yao | male | Canada | Evidence-Based Medicine | Assistant Researcher |
|  | Lihong Yang | female | Guangdong | Evidence-Based Medicine | Assistant Researcher |
|  | Qi Wang | female | Canada | Evidence-Based Medicine | Research Intern |
|  | Runsheng Xie | male | Guangdong | Standardization of Chinese Medicine | Assistant Researcher |
|  | Sha Yao | female | Guangdong | Chinese Medicine Bibliography | Resident doctor |
|  | Shaonan Liu | male | Guangdong | Evidence-Based Medicine | Associate Research Fellow |
|  | Wenjia Chen | male | Guangdong | Standardization of Chinese Medicine | Research Intern |
|  | Wenjie Xu | female | Beijing | TCM Dermatology | Associate Research Fellow |
|  | Yangyang Wang | female | Guangdong | Standardization of Chinese Medicine | Assistant researcher |
|  | Yun Xia | female | Hainan | Chinese Medicine Bibliography | Resident doctor |
|  | Xue Feng | female | Beijing | Standardization of Chinese Medicine | Assistant Researcher |
|  | Liyan Jiang | female | Fujian | Clinical Research Methodology | Assistant Researcher |
|  | Baoyong Lai | male | Beijing | Clinical Research Methodology | Assistant Researcher |
|  | Xiaoqin Wang | female | Canada | Clinical Research Methodology | Assistant Researcher |
|  | Guozheng Zhao | male | Beijing | Clinical Research Methodology | Assistant Researcher |
