# Supplementary material for: Adapting the ADAPTE framework for Traditional Chinese Medicine clinical practice guidelines: a methodological study
Source: Chin Med. 2026 Jan 20;21:42. doi: 10.1186/s13020-026-01323-1 (PMC12817658; doi:10.1186/s13020-026-01323-1)
Supplement: Supplementary file 2 — Supplementary Material 2. [file 13020_2026_1323_MOESM2_ESM.docx]

Adaptation for TCM:Procedures and methods for the adaptation of TCM clinical practice guidelines

**Table of Contents**

SUMMARY OF THE ADAPTE PROCESS 1

Introduction 2

**PHASE ONE – SET-UP**. 5

1.1 Preparation Module. 5

[Step 1. Check whether adaptation is feasible 5](#_Toc29584)

[Step 2. Establish an organizing committee 5](#_Toc32154)

[Step 3. Select a guideline topic 5](#_Toc27704)

[Step 4. Identify necessary resources and skills 6](#_Toc10460)

[Step 5. Complete tasks for the set-up phase 8](#_Toc3433)

[Step 6. Write adaptation plan 9](#_Toc13442)

**PHASE TWO – ADAPTATION**. 13

2.1 Scope and Purpose Module 13

[Step 7. Determine the health questions 11](#_Toc5229)

2.2 Search and Screen Module. 15

[Step 8. Search for guidelines and other relevant documents 13](#_Toc15033)

[Step 9. Screen retrieved guidelines 14](#_Toc25754)

[Step 10. Reduce a large number of retrieved guidelines 15](#_Toc990)

2.3 Assessment Module 19

[Step 11. Assess guideline quality 17](#_Toc13773)

[Step 12. Assess guideline currency 19](#_Toc32125)

[Step 13. Assess guideline content 20](#_Toc10213)

[Step 14. Assess guideline consistency 23](#_Toc5751)

[Step 15. Assess acceptability and applicability of the recommendations 24](#_Toc27341)

2.4 Decision and Selection Module.. 28

[Step 16. Review assessments 26](#_Toc26647)

[Step 17. Select between guidelines and recommendations to create an adapted guideline 27](#_Toc9723)

2.5 Customization Module. 32

[Step 18. Prepare draft adapted guideline 30](#_Toc568)

**PHASE THREE – FINALIZATION**. 34

3.1 External Review and Acknowledgement Module. 34

[Step 19. External review - target audience of the guideline 32](#_Toc22129)

[Step 20. Consult with endorsement bodies 33](#_Toc24295)

[Step 21. Consult with source guideline developers 33](#_Toc13902)

[Step 22. Acknowledge source documents 33](#_Toc7470)

3.2 Aftercare Planning Module .. 37

[Step 23. Plan for aftercare of the adapted guideline 35](#_Toc8961)

3.3 Final Production Module .. 39

[Step 24. Produce final guidance document 37](#_Toc7458)

[Glossary 38](#_Toc6946)

[References 42](#_Toc28268)

[Tool 1: Guideline Development and Implementation Resources 44](#_Toc5470)

[Tool 2: Search Sources and Strategies 46](#_Toc25605)

[Tool 3: Sample Declaration of Conflict of Interest 50](#_Toc31687)

[Tool 4: Consensus Process Resources 54](#_Toc32462)

[Tool 5: Example of Work Plan 55](#_Toc22150)

[Tool 6: PIPOH 58](#_Toc11754)

[Tool 7: Table for Summarizing Guideline Characteristics 61](#_Toc18756)

[Tool 8: Table for Summarizing Guideline Content 62](#_Toc6929)

[Tool 9: AGREE II for Instrument 63](#_Toc24779)

[Tool 10: Quality evaluation of TCM diagnosis based on syndrome differentiation 76](#_Toc10792)

[Tool 11: Sample Currency Survey of Guideline Developers 77](#_Toc23206)

[Tool 12: Sample Recommendation Matrix 78](#_Toc20190)

[Tool 13: Evaluation Sheet – Search and Selection of Evidence 85](#_Toc7884)

[Tool 14: Evaluation Sheet – Scientific Validity of Guidelines (Consistency between Evidence, Its Interpretation and Recommendations) 87](#_Toc12340)

[Tool 15: Evaluation sheet – Acceptability/Applicability 89](#_Toc9112)

[Tool 16: Checklist of Adapted Guideline Content 90](#_Toc4356)

[Tool 17: Checklist of the RIGHT Extension Statement for traditional Chinese medicine (RIGHT-TCM) 91](#_Toc30371)

[Tool 18: Sample External Review Surveys 93](#_Toc28172)

[Tool 19: Table for Reporting on Results of Update Process 97](#_Toc14644)

# SUMMARY OF THE ADAPTE PROCESS

PHASES TASKS ASSOCIATED MODULES


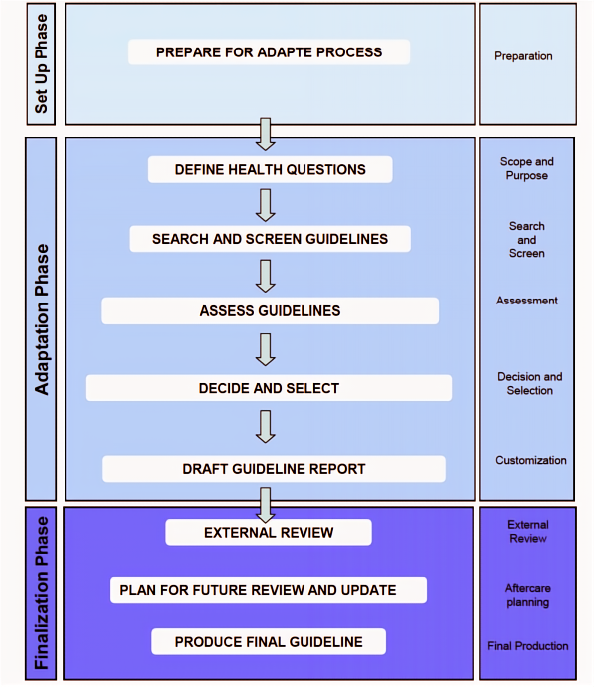


**Introduction**

The development and updating of high-quality [practice guidelines r](#bookmark125)equire substantial resources. Most organisations are under pressure to produce more guidelines in a shorter time with increasingly limited resources. While the key methods for guideline development have converged over the years, a large number of organisations worldwide do produce guidelines on the same topic. In order to take advantage of existing guidelines and reduce this duplication of effort, guideline adaptation has been proposed as an option for guideline development (1,2).

However, the [cultural a](#bookmark126)nd organizational differences between and within countries can lead to legitimate variations in [recommendations,](#bookmark127) even when the evidence base is the same. This means that guidelines produced in one setting may not necessarily be appropriate for another, without careful consideration and/or contextualization. The ADAPTE Collaboration has developed a systematic approach to aid in the adaptation of guidelines and has produced this resource toolkit for that purpose. In order to better utilize the existing high-quality TCM guidelines and to form adapted guidelines that are applicable to different countries, regions, administrative regions or different versions, ADAPTE for TCM has been developed based on the ADAPTE toolkit.

**Definition of guideline adaptation**

The ADAPTE Collaboration defines [guideline adaptation](#bookmark128)as the systematic approach to considering the use and/or modification of (a) guideline(s) produced in one cultural and organizational setting for application in a different context. Adaptation can be used as an alternative to *de novo* guideline development – where guidelines currently exist or for customizing (an) existing guideline(s) to suit the local context.

**Aim of guideline adaptation**

The overall objective of adaptation is to take advantage of existing [guidelines i](#bookmark129)n order to enhance the efficient production and use of high-quality adapted guidelines. The adaptation process described in this resource toolkit has been designed to ensure that the final [recommendations](#bookmark130) address specific [health questions r](#bookmark131)elevant to the context of use and address the needs, priorities, legislation, policies, and resources in the target setting, without undermining the validity of the resulting recommendations.

The adaptation process is based on the following core principles

• Respect for the [evidence-based principles o](#bookmark132)f guideline development

• Reliable and consistent methods to ensure the quality of the adapted guideline

• Participative approach, involving all key stakeh[olders, to f](#bookmark133)oster acceptance and ownership

of the adapted guideline

• Explicit consideration of context during adaptation to ensure relevance for local practice

• Transparent reporting to promote confidence in the recommendations of the adapted guideline

• Flexible format to accommodate specific needs and circumstances

• Accountability to the primary guideline sources Application Scenarios for Adaptation of Chinese Medicine Clinical Guidelines

• Applied to different countries, such as those where TCM is legalized, adapting to local medical practices and regulations by combining the experiences of different countries

• Applied to different provinces in China, adapted to the local differences, epidemiological characteristics and distribution of medical resources in their respective provinces.

• Adaptation of guidelines developed by national societies for use at the grassroots level, where medical resources have their own particularities, and moderate adaptation of the guidelines is made in the light of the actual situation in order to ensure the feasibility and effectiveness of implementation.

**Outline of adaptation process**

The adaptation process consists of three main phases (Set-up Phase, Adaptation Phase, and Finalization Phase), each with a set of modules. Each module includes several steps, products and deliverables, skills and organizational requirements, and tools.

| **SET-UP PHASE** | **ADAPTATION PHASE** | **FINALIZATION PHASE** |
| --- | --- | --- |
| Preparation Module | Scope and Purpose Module Search and Screen Module Assessment Module  Decision and Selection Module Customization Module | External Review and Acknowledgment Module Aftercare Planning Module Final Production Module |

**Set-up Phase:** Outlines the necessary tasks to be completed prior to beginning the adaptation process (e.g., identifying necessary skills and resources) with a first step of determining whether adaptation is feasible. Readers familiar with guideline development will already have experience with these tasks.

**Adaptation Phase**: Assists users in moving from selecting a topic to identifying specific health questions; searching for and retrieving guidelines; assessing the consistency of the evidence and the guideline quality, currency, content, and applicability; decision making around adaptation; and preparing the draft adapted guideline.

**Final Phase**: Guides the user through the process of obtaining feedback on the document from stakeholders who will be impacted by the guideline, consulting with the source developers of guidelines used in the adaptation process, establishing a process for the review and updating of the adapted guideline, and creating a final document.

**Purpose of this resource toolkit**

This resource toolkit provides a practical guide to the adaptation of [practice guidelines.](#bookmark134) The explicit approach described in the resource toolkit is intended to be useful to guideline users and implementers such as local healthcare authorities and organizations, guideline development organizations, and international healthcare organizations. The methods aim to suit the needs of abroad range of stakeholders (from novices to those experienced with guideline development and groups with lesser or greater resources).

This resource toolkit is not a guide for developing *de novo* guidelines and does not provide details on guideline[dissemination](#bookmark135)and[implementation.](#bookmark136) Several resource toolkits on these aspects are freely available via the Internet from institutions such as the National Institute for Health and Clinical Excellence (NICE), the Scottish Intercollegiate Guidelines Network (SIGN), the National Health and Medical Research Council (NHMRC), the New Zealand Guideline Group (NZGG) and the Chinese Medicine Association (see [Tool 1 –](#bookmark137) Guideline Development and Implementation Resources).


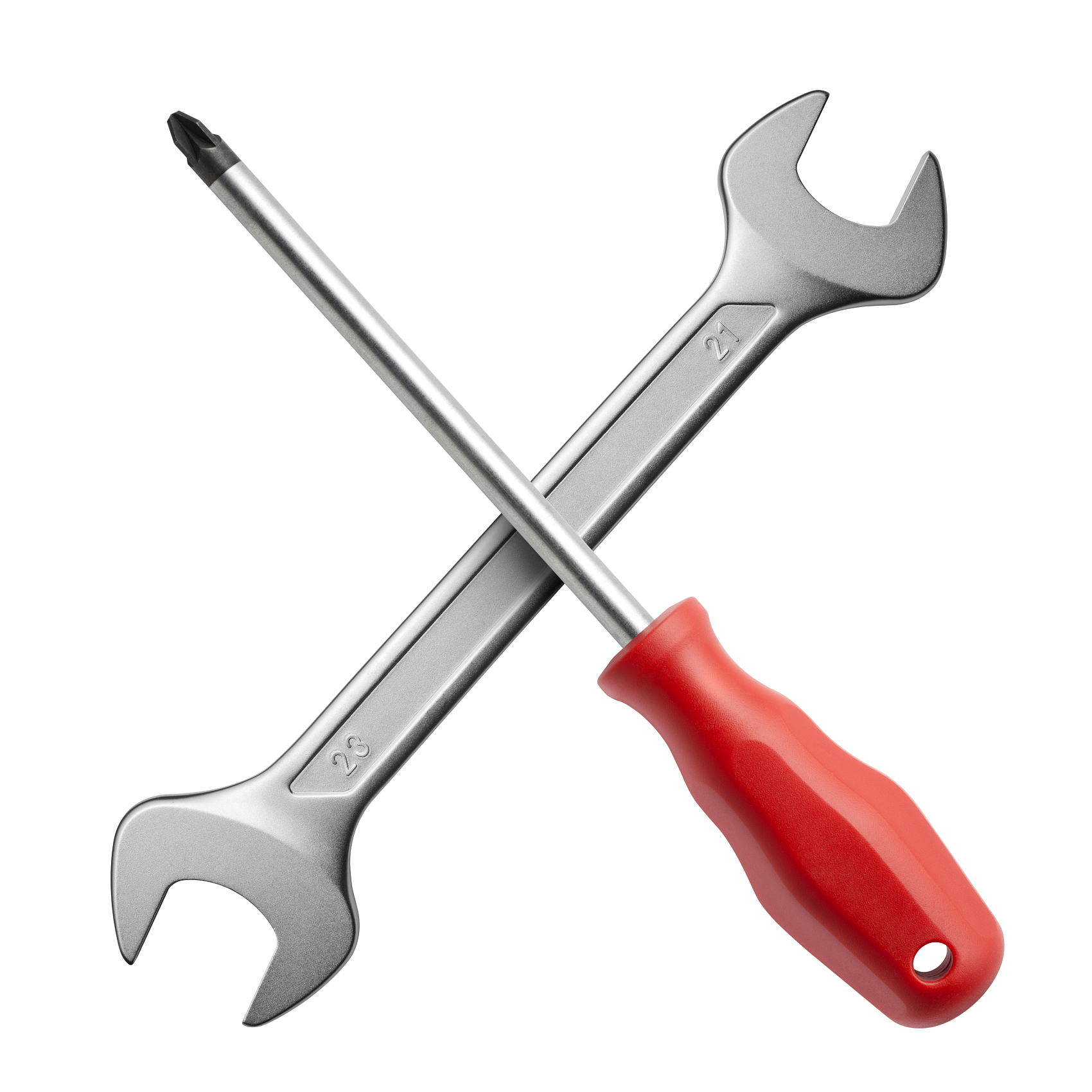


[**Tool 1**](#bookmark138)– **Guideline Development and Implementation Resources**

**How to use this resource toolkit**

The adaptation process described in this resource toolkit has multiple applications. For example, a group maybe interested in selecting one specific guideline for adaptation to the local context. Others may want to identify all high-quality guidelines that respond best to the health questions and healthcare situations of their context and then customize a guideline that meets their needs. In addition, this adaptation process can be applied to guidelines for health promotion, screening, diagnosis, treatment, follow-up, or other interventions in any disease area.

The process is designed to be flexible, depending on the application. Not all modules maybe relevant to the users’ needs. For example, those wishing to adapt a single guideline will not need to perform a systematic search for all guidelines related to the health question(s) (Adaptation Phase – Search and Screen Module). For those users experienced in guideline development, some of this information will be familiar and maybe redundant. However, we suggest that all users read the complete resource toolkit to have a sense of the process from beginning to end.

The Set-up Phase outlines the necessary tasks to be completed prior to beginning the adaptation process (e.g., identifying necessary skills and resources). Readers familiar with guideline development will already have experience with these tasks.

| **Steps** | **Pruducts/**  **Deliverables** | **Skills and Organizational Requirements** | **Tools** |
| --- | --- | --- | --- |
| 1.Check whether adaptation is feasible  2.Establish an organizing committee  3. Select a topic  4. Identify skills and resources needed  5. Complete set-up tasks  6. Write protocol | •Orgnizing committee established  • Topic identified  • Panel(expert groups, working groups) selected  •Protocol completed | Clinical expertise  Methodological expertise  Managerial and  administrative skills | [Tool1](#bookmark139)–Guideline Development and Implementation Resources  [Tool2](#bookmark140)–Search Sources and Strategies  [Tool 3](#bookmark141)– Sample Declaration of Conflict of Interest  [Tool4](#bookmark142)–Consensus Process Resources  [Tool5](#bookmark143)– Work Plan Example |

**Step 1. Check whether adaptation is feasible**

Begin by identifying the topic of the guideline, even if there are existing guidelines for a specific topic, we suggest checking whether any other guidelines have been produced or are currently being developed on the selected topic by searching the Websites of guideline clearinghouses and specialty organisations (see [Tool 2](#bookmark144)– Search Sources and Strategies). In some situations, the decision maybe to adapt a specific guideline rather than searching for a larger number of potential source guidelines. If no guidelines related to the topic area exist, a decision will need to be made about whether a guideline should be created *de novo*—for those organizations with the resources to develop guidelines.


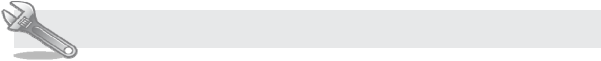


[**Tool 2**](#bookmark145) **search sourees and strategies**

**Step 2. Establish an organizing committee**

An organizing committee should oversee the adaptation process. In the Set-Up Phase, the committee responsibilities will include determining the project scope, organizational and governance structures (e.g., working group or multidisciplinary panel members), terms of reference, and development of an adaptation plan. The composition of the organizing committee can be implemented with reference to chapter 4 of the Manual of Integrated Chinese and Western Medicine Guidelines (3) and DB44 / T 2218.3- -2019 of the Clinical Practice Guidelines of Traditional Chinese Medicine (Integrated Chinese and Western Medicine) Clinical Practice Guide Construction and Management (4) edited by Lu Chuanjian and others. It is composed of expert group, working group and external audit group.

**Step 3. Select a guideline topic**

In some cases, the need for a guideline on a particular[topic w](#bookmark146)ill already have been identified. In other cases, a group may need to select a topic. There area number of criteria that can be used to identify and prioritize areas for best practice and guideline adaptation (2). For example, these criteria might include:

• Burden of disease (high burden on patients, social health care due to high rates of disability, mortality, morbidity caused by disease)

• Demand for TCM guidelines based on areas of guideline application

• Use of interventions (effectiveness, legitimacy, feasibility, cost)

• Burden associated with disease (high disability, mortality, morbidity due to disease, high burden on patients, social health care)

• Concerns about diversity in clinical practice and the availability of baseline data for current practice

• Cost-effectiveness analyses associated with different clinical practice scenarios in regions where the guidelines are being applied.

• Potential for the guideline to effectively influence clinical practice

• Potential to improve quality of care and/or patient outcomes (e.g., survival or quality of life)

• Existence of relevant high-quality evidence-based guidelines on TCM (an initial determination can be made based on Step 11). (Please refer to Tool 2 for searching)

**Step 4. Identify necessary resources and skills**

In addition to ensuring that there are existing guidelines to support adaptation, there need to be sufficient resources to complete the process, resources that include the following:

• Commitment by the panel members to at least one face-to-face meeting and to conference calls

• Commitment by the panel members, outside of meetings, to review all documents

• Coverage of meeting costs

• Possible honorariums for panel members to cover the time spent appraising guidelines

• Availability of project management personnel and administrative support for guideline collection, storage, documentation; and meeting coordination

• Coverage of the costs of implementing the guideline (if relevant)

The credibility of the guideline quality appraisal process rests, in large part, on the credibility of the assessment team members of the panel (5). Who is involved and the skills that they bring to the panel are important. The group should include individuals from among key [stakeholders a](#bookmark147)ffected by the guideline.

The following skills should be represented on the panel:

• Clinical knowledge in the topic area—knowledge of the issues related to the application of the guideline in local practice as well as TCM (integrated Chinese and Western medicine) diagnosis and treatment in the subject areas and related sources, including relevant brief descriptions of ancient Chinese medicine texts to the latest research results in modern times; knowledge related to clinical pharmacology (Chinese pharmacy)

• Personal experience with the topic area (e.g., experience gained from living with the disease, having undergone the intervention, or caring for someone with the disease)—to ensure that issues related to patient/consumer needs are discussed and that salient outcomes such as quality of life are considered

• Policy/administrative expertise—to identify the impact of the guideline on an organization and to anticipate resource requirements resulting from implementing the guideline

• Methodological expertise (e.g., health services researchers, Health economic analysts, etc.)—knowledge of research design and knowledge in critical appraisal and guideline appraisal play a role in. Health economics-related professionals help guideline development teams make evidence-based economic assessments by analyzing the costs and outcomes of different treatment options. Ensure that the treatment options recommended by the guidelines are not only clinically effective but also economically feasible educating other panel members on issues related to the systematic and rigorous nature of the process and provides a methods resource • Information retrieval expertise—knowledge of databases and literature searching

• Managerial skills—to manage the timelines of the project, setup meetings and conference calls, and ensure that all documents are circulated to the panel

• [Implementation expertise—knowledge of implementation i](#bookmark148)ssues, including how to develop a plan for putting the guideline into practice and spearhead the implementation

• Facilitation skills—to help the panel function effectively, ensure all panel members are given opportunities to contribute, and help the panel achieve its aims

Multidisciplinary teams (e.g., TCM clinical, guideline methodology, literature information retrieval, clinical pharmacy (Chinese pharmacy), health cost analysis related, etc.) are important if the guideline addresses issues that affect multiple provider groups. The involvement of multiple disciplines ensures that issues related to guideline application, evidence support behind recommendations, and impact on patients can be fully considered (1,5).

**Step 5. Complete tasks for the set-up phase**

By the end of this phase, the following items need to be completed or considered:

• Terms of reference: Such terms should be drawn up by either the organizing committee or the panel and could include the scope of the work to be completed, how the membership is constituted, time commitment required and how often the panel should meet. The terms of reference need to be shared with all panel members so that they understand and agree to their involvement in the process.

• Declaration of conflict of interest: ADAPTE encourages all panel members to complete and sign a declaration of conflict of interest. The panel should be aware of the potential bias or vested interests/conflicts of interest of any member who might have been involved in the development of one of the guidelines considered for the adaptation process. Decisions will need to be made as to whether such potential conflicts create a concern or not, and, if they do, how to deal with that concern.

[**Tool 3**](#bookmark149)– **Sample Declaration of Conflict of Interest**


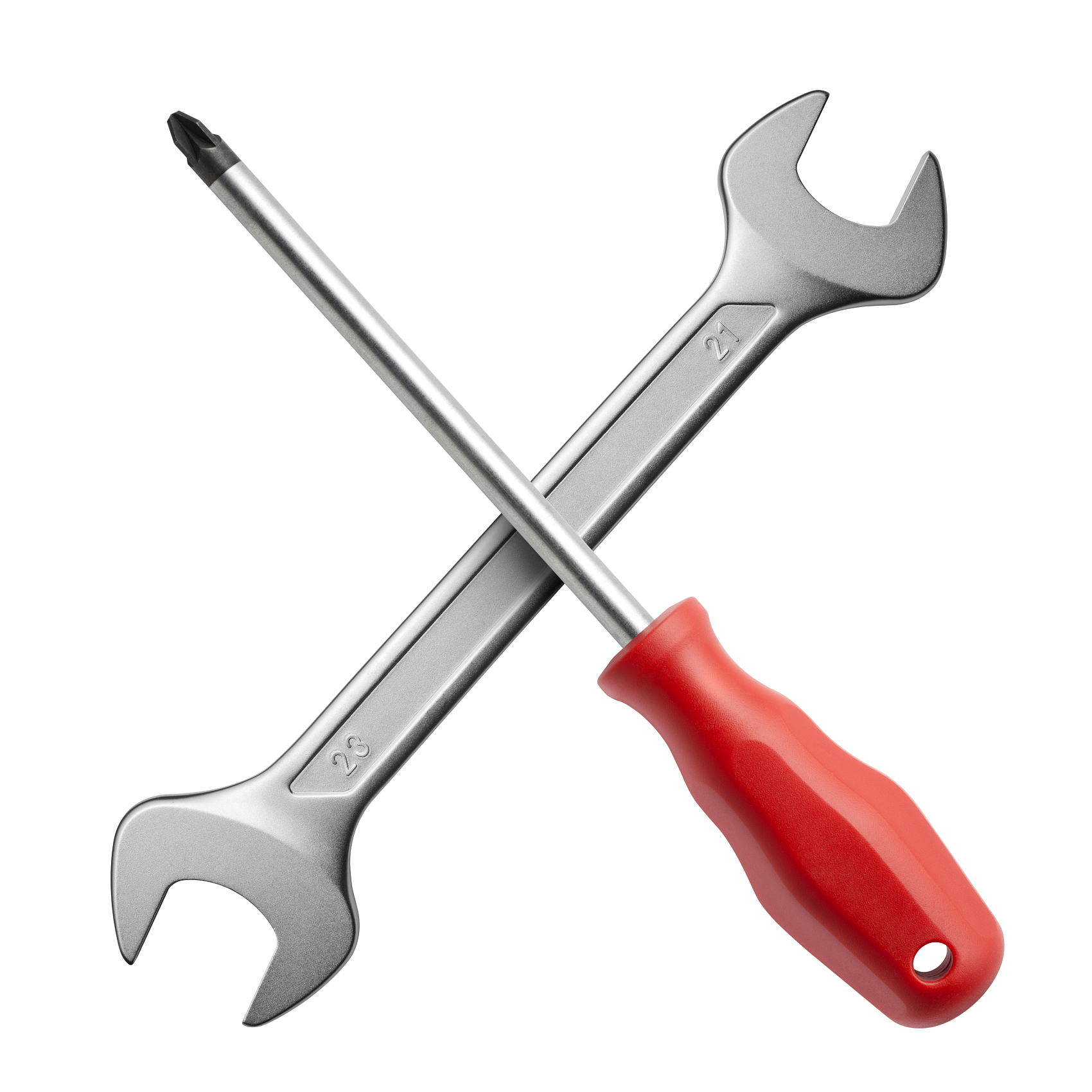


• Consensus process: A decision should be made by the organizing committee or panel as to how the panel will manage decisions (e.g., through either a formal or informal consensus process) and how this process will be reported in the final document.

[**Tool 4**](#bookmark150)– **Resources on Consensus Processes**


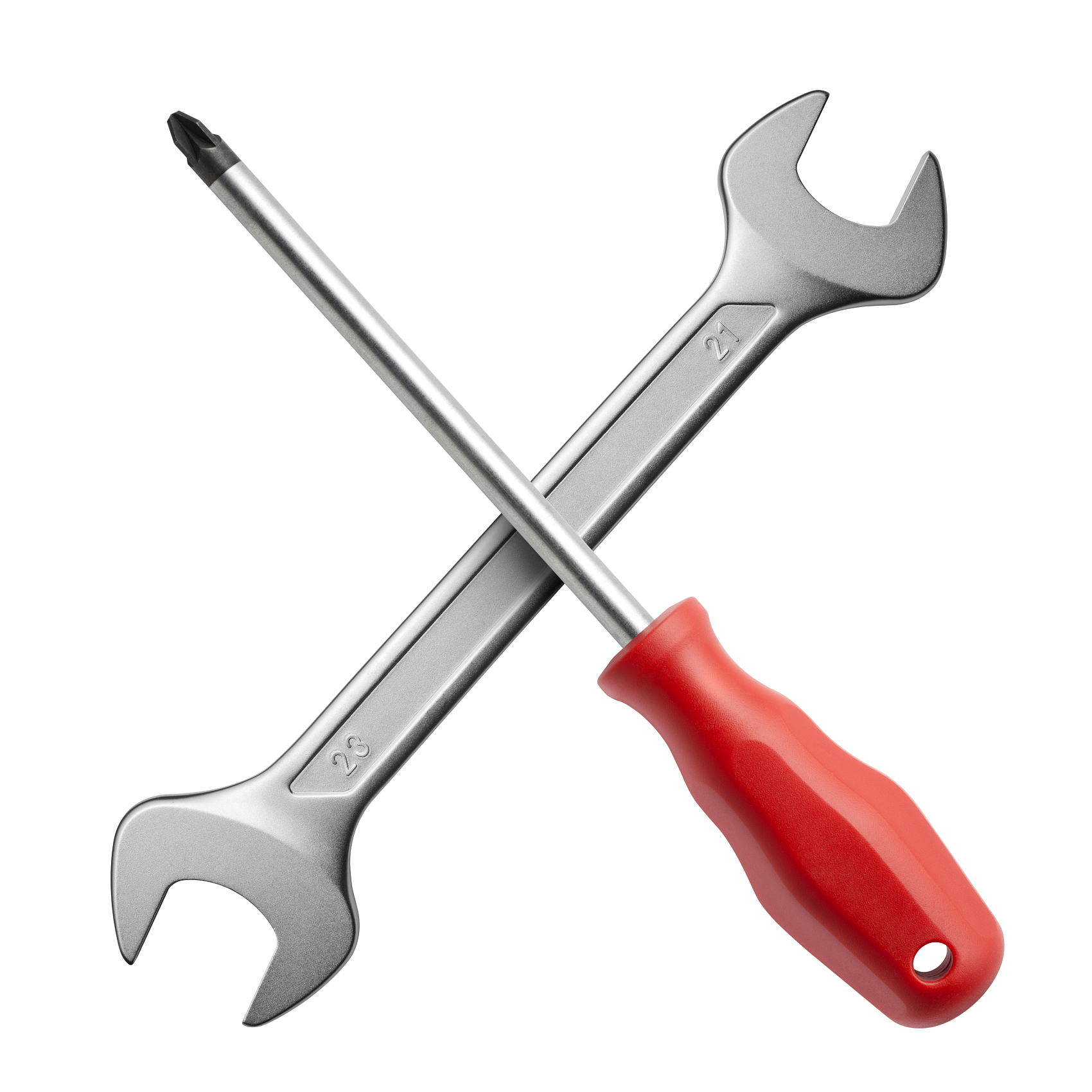


• Potential endorsement bodies: The committee should decide whether it would be helpful to have someone or some organization endorse the adapted guideline. If so, they should consider involving a representative of the endorsement body (e.g., hospital administration, professional body, or home care authority) in the process as a member of the panel or as part of the external review process of the draft guideline.

• Guideline authorship: A decision should be made as to who will be responsible for writing the draft adapted guideline and the final report and about the principles of authorship.The order of authorship needs to be determined (e.g., name of the member responsible for writing the guideline, name of the chair, and name of the group). Group authorship could also be considered.

• [Dissemination a](#bookmark151)nd [Implementation S](#bookmark152)trategies: Potential publications should be considered, for example, a publication on the organization’s Website and/or a manuscript submitted to a journal for publication. The eventual implementation of the adapted guideline should be considered throughout the adaptation process, for example, the context of implementation should be taken into account when reviewing possible recommendations. Tool 1 provides a list of available resources that provide good strategies for implementation.

[**Tool 1**](#bookmark153)– **Guideline Development and Implementation Resources**


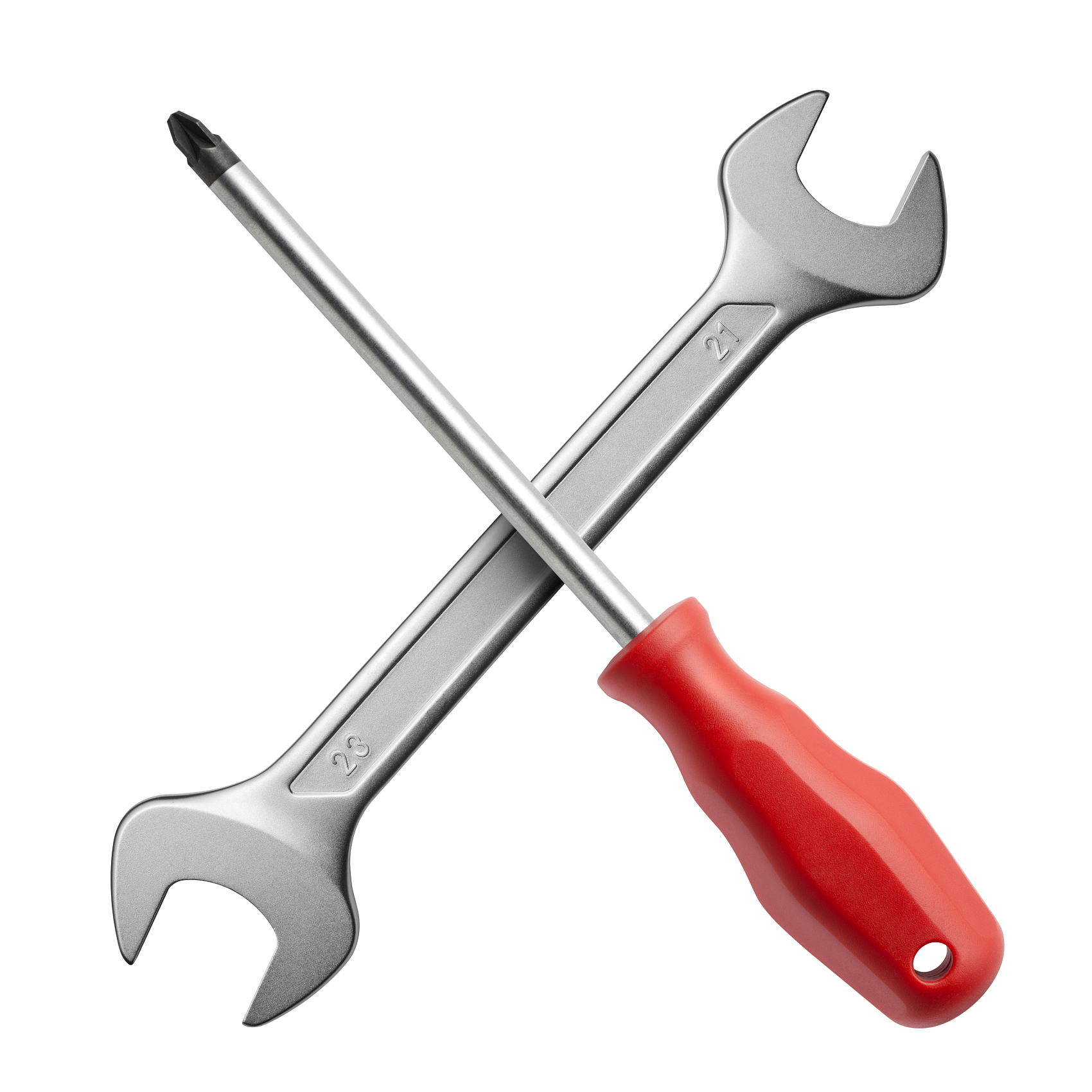


**Step 6. Write adaptation plan**

At the completion of the preliminary phase, we recommend that the organizing committee and the panel agree about a plan that outlines the adaptation process to be followed. The formalized plan might include the following headings:

• Introduction

• Topic area

• Panel members, credentials, and declarations of conflicts of interest

• Panel Terms of Reference

• Modules to be followed

• Timeline for completion of the adaptation process and committed target datefor completion, including meeting schedule

• Funding source(s)

Throughout the process, each decision taken by the organizing committee and the multidisciplinary panel should be well documented to make the process transparent. A person needs to be identified to manage and communicate this plan to all panel members. It is recommended that the guideline planner be registered if necessary.

| 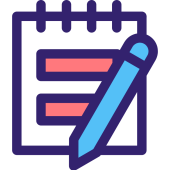 | **Illustration – Set Up Phase** |
| --- | --- |
| Traditional Chinese medicine treatment of psoriasis vulgaris was selected as a topic for adaptation by one country or region. The main reason for choosing this theme is that psoriasis is a common chronic inflammatory skin disease with high incidence and easy to recur, and its pathogenesis has not been clarified, but modern research has confirmed that psoriasis is a genetically related, immune-mediated chronic inflammatory disease induced by multiple factors, and that the application of immunosuppressants and immune-targeted biologics can, to a certain extent, effectively control the occurrence and persistence of psoriasis. However, the long-term efficacy of the treatments to date is still unsatisfactory. TCM evidence-based treatment can achieve better clinical efficacy and longer remission period, and the combination of TCM and Western medicine in the treatment of this disease has greater advantages and broad prospects. Existing systematic evaluation suggests that the overall efficacy can be effectively improved by grasping the timing of TCM treatment, Western medicine treatment and their respective characteristics and strengths. An organizing committee was formed to carry out the adaptation process; an expert in the field of the specialty was identified to chair the meeting. Adaptation rather than rewriting was chosen because the organizing committee had been made aware of a number of reliable TCM/integrated Chinese and Western medicine treatment guidelines for psoriasis vulgaris written by recognized guideline developers that were currently being used by practitioners. The committee decided to retrieve as many guidelines as possible rather than adapting only one.  The expertise and skills required for the expert group were identified and included the following: three TCM dermatologists, a general practitioner, a nurse with experience in psoriasis chronic disease management, a patient representative, a methodologist, and representatives from professional organizations (one from a national or provincial TCM dermatology society and one national or provincial organization focused on TCM guideline development). The organizing committee also formed a working group that would be responsible for collecting and retrieving guidelines, calculating guideline quality scores, assessing guideline timeliness, compiling a summary of recommendations, providing feedback on all assessment data and sending draft guidelines to an external review panel for review and consultation, and contacting potential organizing committee members by letter and follow-up phone calls.  Letters contacting potential organizing committee members outline their tasks on the organizing committee and the total time commitment required (Terms of Reference). A small honorarium is available for participation in this work. Members' meeting expenses (airfare and accommodation) are covered by the Organizing Committee. After agreeing to participate, each member of the organizing committee signed a conflict of interest statement - no conflicts were found.  The organizing committee prepared a short protocol outlining the process to be followed by the organizing committee, which included the background and rationale for the adaptation, the topic area, the expert group members, the consensus process to be followed, the modules to be followed, and the source of funding. An example work plan with timeline is provided in *T*[*ool 5 – W*](#bookmark154)*ork Plan Example*.  [**Tool 5**](#bookmark155)– **Example of Work Plan –**  **Cervical Cancer Screening Guidelines Panel** 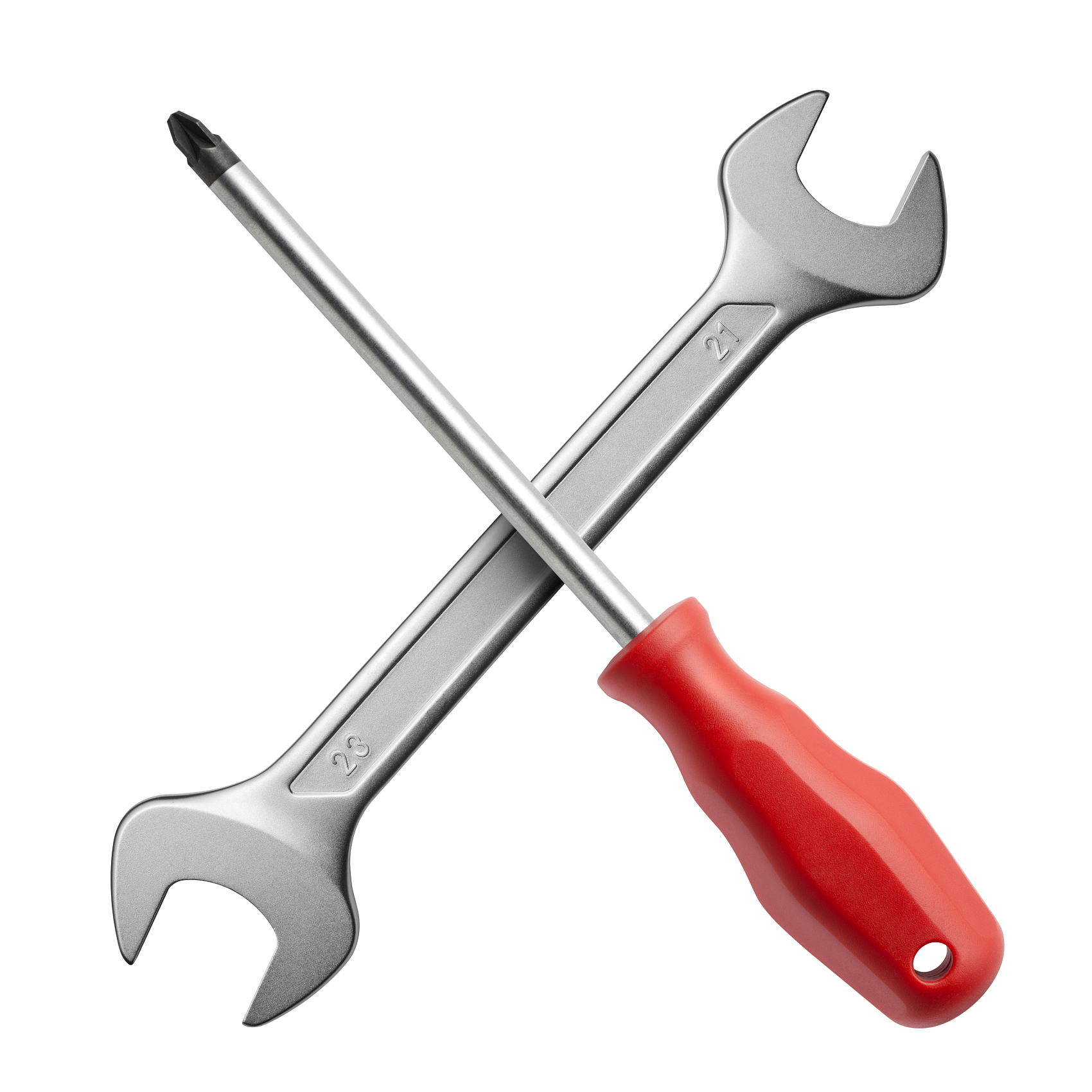 | |

**ADAPATION PHASE**

**2.1 Scope and Purpose Model**

The Adaptation Phase assists users through the process of selecting a topic to identifying specific

health questions, searching for and retrieving guidelines, assessing the guideline quality, currency, content, consistency and applicability, decision making around adaptation, and preparing the draft adapted guideline.

| **Steps** | **Pruducts/**  **Deliverables** | **Skills and Organizational Requirements** | **Tools** |
| --- | --- | --- | --- |
| 7. Determine the health questions | • List of health questions to be included and those that are to be specifically excluded in the projected guideline | Clinical Specialist in Chinese Medicine/Chinese and Western Medicine, Methodological expertise | Tool 6 – PIPOH |

**Step 7. Determine the health questions**

Once abroad [topic a](#bookmark156)rea is identified, it is very important to clarify the specific purpose and parameters of the chosen guideline topic by developing a series of structured key [questions (](#bookmark157)6). The definition of a set of clear and focused health questions is an important consideration for successfully completing the adaptation process and will ensure that the final adapted guideline is applicable in the users’ context. Conversely, some questions can and should be specifically excluded from the project.

The use of the following five items (PIPOH) will help to define the health questions and coverall relevant aspects:

• The **P**opulation concerned and characteristics of disease or condition

• The **I**ntervention(s) (or diagnostic test, etc.) of interest

• The **P**rofessionals to whom the guideline will be targeted

• The expected **O**utcomes including patient outcomes (e.g., improved disease free survival, improved quality of life); system outcomes (e.g., decrease in practice variation); and/or public health outcomes (e.g., a decrease in cervical cancer incidence)

• The **H**ealthcare setting and context in which the guideline is to be implemented

Existing guidelines identified in the preliminary phase may help in defining the health questions. A quick survey of the guideline content may reveal additional health questions.

[**Tool 6**](#bookmark158)– **PIPOH**


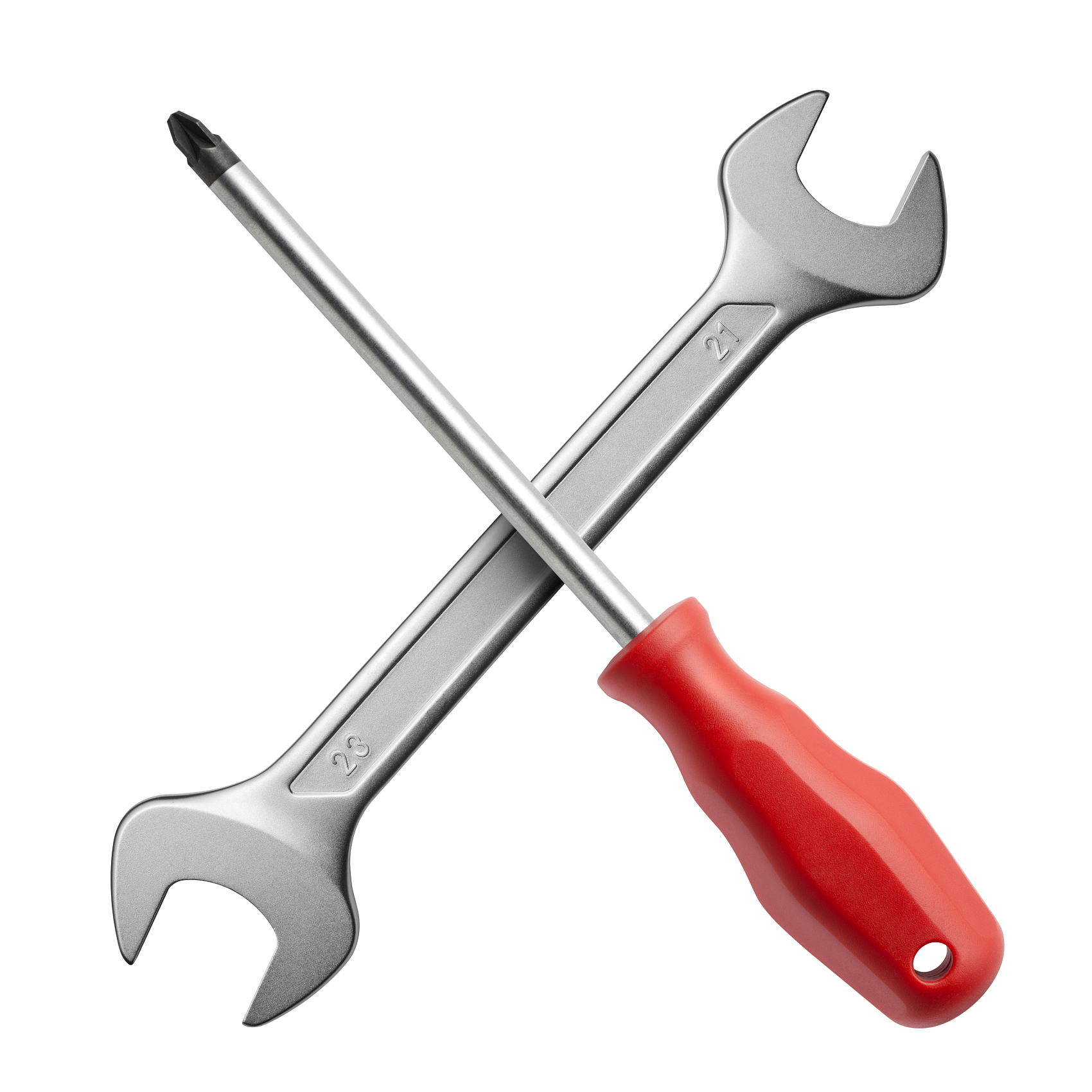


| 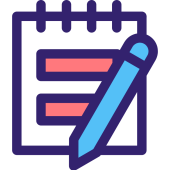  **Illustration – Determining the health question using the**  **PIPOH instrument**  The organizing committee used the PIPOH tool to help define their health questions.  **P**opulation: They decided to make TCM treatment recommendations only for patients with psoriasis vulgaris (e.g., excluding arthritic, pustular, and erythrodermic psoriasis vulgaris).  **I**ntervention: The interventions selected were traditional Chinese medicine (TCM) therapies (including herbal medicine, acupuncture, roasting, tuina, etc.). More specifically, the organizing committee decided that the guideline search would not be limited to any particular TCM therapy.  **P**rofessionals: Typically, psoriasis diagnosis and treatment is one of the healthcare interventions mainly performed by TCM practitioners, integrative TCM clinicians or TCM nurse practitioners. Therefore, the adapted guidelines will be designed with these target groups in mind.  **O**utcomes and outcome measures: Ideally, the guideline will encourage Chinese medicine practitioners, integrated Chinese and Western medicine practitioners to follow the relevant diagnostic and therapeutic requirements in the guideline for TCM or integrated Chinese and Western medicine treatment. There are great variations in practice across the country and at all levels of hospitals, and the popularity of Chinese medicine is low in some areas, especially in grassroots communities. TCM treatment should be able to improve patients' quality of life, reduce disease recurrence rates and reasonably reduce costs.  **H**ealthcare setting and context: The organizing committee hopes that the guidelines will be applicable to primary care.  Through using the PIPOH, the organizing committee decided on the following clinical question:  For patients with psoriasis vulgaris attending primary care settings, is it appropriate to use TCM internal treatment (syndrome differentiation, traditional Chinese patent medicines and simple preparations, single herb extract of Chinese medicine), TCM external treatment (drug external treatment (drug external treatment, filiform needle therapy, ear acupuncture therapy, fire needle therapy, moxibustion therapy, blood pricking and cupping therapy, cupping therapy, acupoint catgut embedding therapy)? |
| --- |

**ADAPATION PHASE**

**2.2 Search and Screen Module**

| **Steps** | **Pruducts/**  **Deliverables** | **Skills and Organizational Requirements** | **Tools** |
| --- | --- | --- | --- |
| 8. Search for guidelines and other relevant documentation   1. Screen retrieved guidelines   10. Reduce total number of guidelines if there are more than can be dealt with by the panel | • Set of potential  [source guidelines](#bookmark160)  • List of excluded guidelines | Search–Clinical and methodological expertise in Chinese medicine, information retrieval skills  Screen–Clinical and methodological expertise in TCM | [Tool 2](#bookmark161)– Search Sources and Strategies  [Tool 7](#bookmark162)– Example Table for Recording the Guideline Characteristics  [Tool 8](#bookmark163)– Example Table for Recording the  Clinical Content of Guidelines  [Tool 9](#bookmark164)– AGREE II for TCM scoring Forms and Scoring Methods |

If the panel decides to identify all guidelines related to a topic area, a systematic search needs to be conducted. An initial screening of those guidelines found by the search will eliminate those that are not relevant based on predefined inclusion/exclusion criteria. These decisions need to be documented.

**Step 8. Search for guidelines and other relevant documents**

Based on the key[question(s) d](#bookmark166)efined in the Scope and Purpose Module, a search strategy can be developed and added to the project documentation. Inclusion and exclusion criteria, for example, the year of development, language, and guideline developer group, should be determined a priori (6). The information should guide the search, and an information scientist can be a useful resource to help with designing the strategy. A reasonably comprehensive search for guidelines should be undertaken to identify the most relevant guidelines to consider for adaptation. In some situations, the decision maybe to adapt a specific guideline rather than searching for a larger number of potential [source guidelines.](#bookmark167) This decision, as well as the reasons for it, should be clearly stated in the guideline report.

Since guidelines may not be published in journals, and not indexed in bibliographic databases, the search should start in guideline clearinghouses such as the US National Guideline Clearinghouse (www.guideline.gov) and the Guidelines International Network [(www.g-i-n.net/)](http://www.g-i-n.net/)) or in country-specific databases (https://www.ttbz.org.cn/Home/Standard). In addition, the Web sites of organisations developing guidelines and of relevant specialty societies, literature databases (including China Knowledge, Medline, Cochrane Library, etc.) should be consulted.

[**Tool 2**](#bookmark168)  **search sources and strategies**


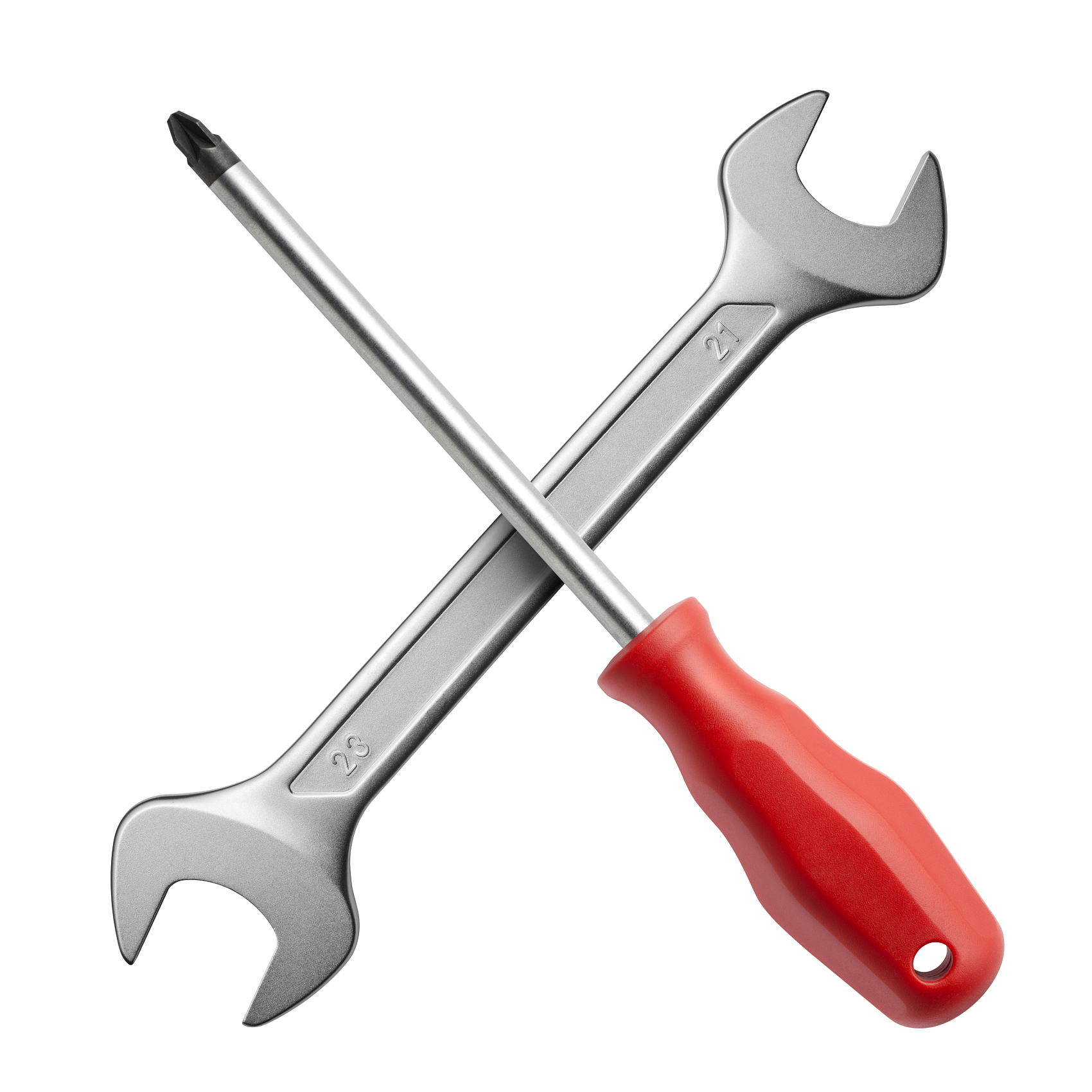


In addition to the guideline websites, complementary searches can be conducted through databases (including China Knowledge, Medline, Cochrane Library, etc.). Databases search such as MEDLINE [(www.ncbi.nlm.nih.gov/entrez/query.fcgi)](http://www.ncbi.nlm.nih.gov/entrez/query.fcgi)) search using a standardised search strategy may yield additional guidelines. Terms to be used include guideline [Publication Type] OR practice guideline [Publication Type] OR recommendation*[Title] OR standard*[Title] OR guideline*[Title], in combination with terms related to the clinical topic.

Internet search engines such as Google, AltaVista, and Yahoo can also be used to locate guidelines. As with other searches, the inclusion and exclusion criteria for the Internet search should be well defined. A recent study has revealed that guidelines posted on the Internet can be of equal or higher quality than guidelines published in the periodical literature (7).

We recommend summarising the following characteristics of the retrieved guidelines in a table:

• Developing organisation/authors

• Date of publication, posting, and release

• Country/language of publication

• Date of posting and/or release

• Dates of the search used by the source guideline developers

Note: A good-quality older guideline could be a good base on which to develop a new guideline. The notion of ‘up-to-date’ may vary with the clinical or health area; in some areas, best available data are regularly modified, whereas in other areas, new data are rarer [see [Assessment Module](#bookmark169) –[Guideline Currency]](#bookmark170).

[**Tool 7** –](#bookmark171) **Example Table for Recording the**

**Characteristics of Guidelines**


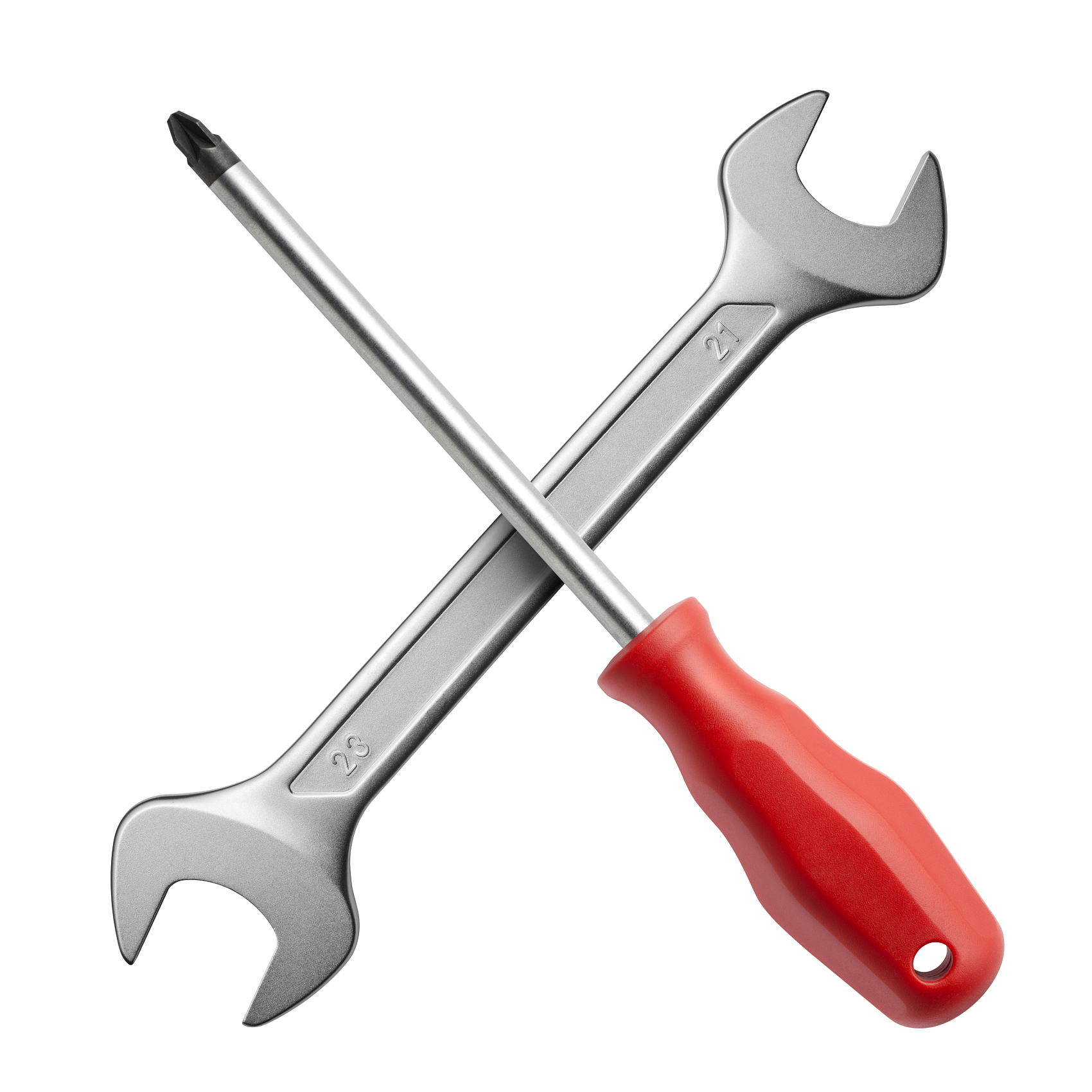


As well as guidelines, an additional search should be conducted to identify any other relevant documents such as recent systematic reviews or health technology assessments reports published since the preparation of the retrieved guidelines. This documentation might be used to confirm whether an update of the evidence is necessary and/or to fill in gaps not covered by retrieved guidelines.

**Step 9. Screen retrieved guidelines**

The objective of this step is to select guidelines for further appraisal. A preliminary assessment of the health questions covered by the retrieved guidelines should be carried out to eliminate those that are clearly not relevant to the defined key questions. Other criteria such as the guideline publication date should be decided upon in advance by the panel in order to screen out

guidelines.

[**Tool 8** –](#bookmark172) **Example Table for Recording the Clinical**

**Content of Guidelines**


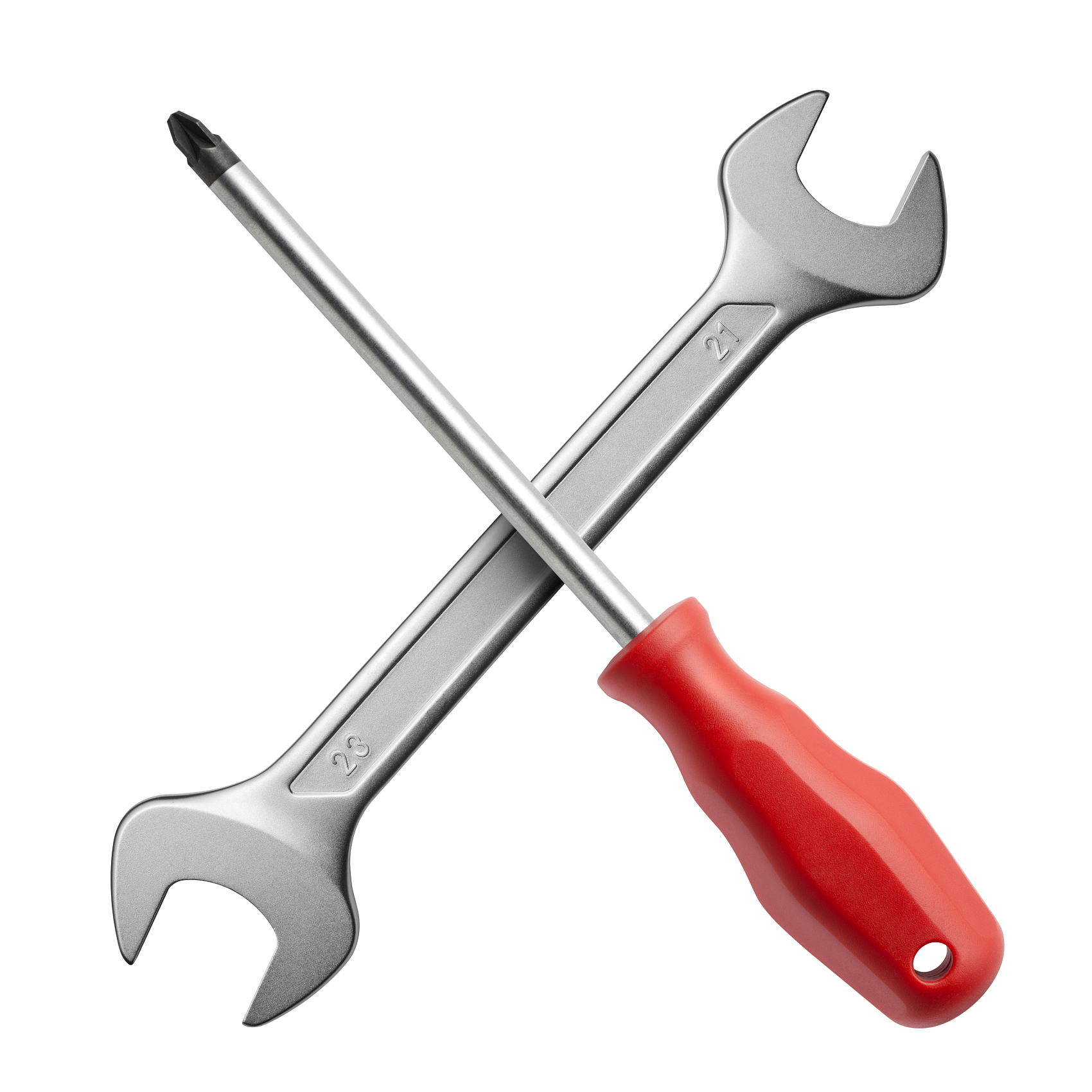


In the case where existing guidelines do not coverall the required topic components, the panel will need to make decisions about modifying the scope of their topic, changing their questions to correspond with[source guideline q](#bookmark173)uestions, modifying the list of health questions, or looking for systematic reviews, health technology assessments reports, or current research articles that would enable them to write their own recommendations for those areas where no recommendations exist. If some denovo work is required, users may find development manuals such as those produced by the National Institute for Health and Clinical Excellence (NICE), the Scottish Intercollegiate Guidelines Network (SIGN), the National Health and Medical Research Council (NHMRC), and the Canadian Medical Association (CMA) helpful.

For each guideline found, the decision to include or exclude should be recorded, along with the reason(s) for any exclusions.

**Step 10. Reduce a large number of retrieved guidelines**

If a large number of potentially relevant guidelines are found during the search, the expert groups and working groups must decide whether or not to reduce the number of guidelines, given the potential time and work burden of the appraisal process. Depending on the guideline, the appraisal process might take approximately one and a half hours per guideline, a substantial time commitment if a large number of guidelines must be reviewed (7). If the panel decides to reduce the number of guidelines to be assessed, the criteria for exclusion at this stage must be made explicit.

One way to reduce the number of guidelines for final approval is to use the rigour dimension of the AGREE II for TCM instrument (see[Assessment Module 2.3 – Assess guideline quality)](#bookmark174) (9).

[**Tool 9**](#bookmark175)–**AGREE II for TCM Scoring Forms**

**and Scoring Methods**


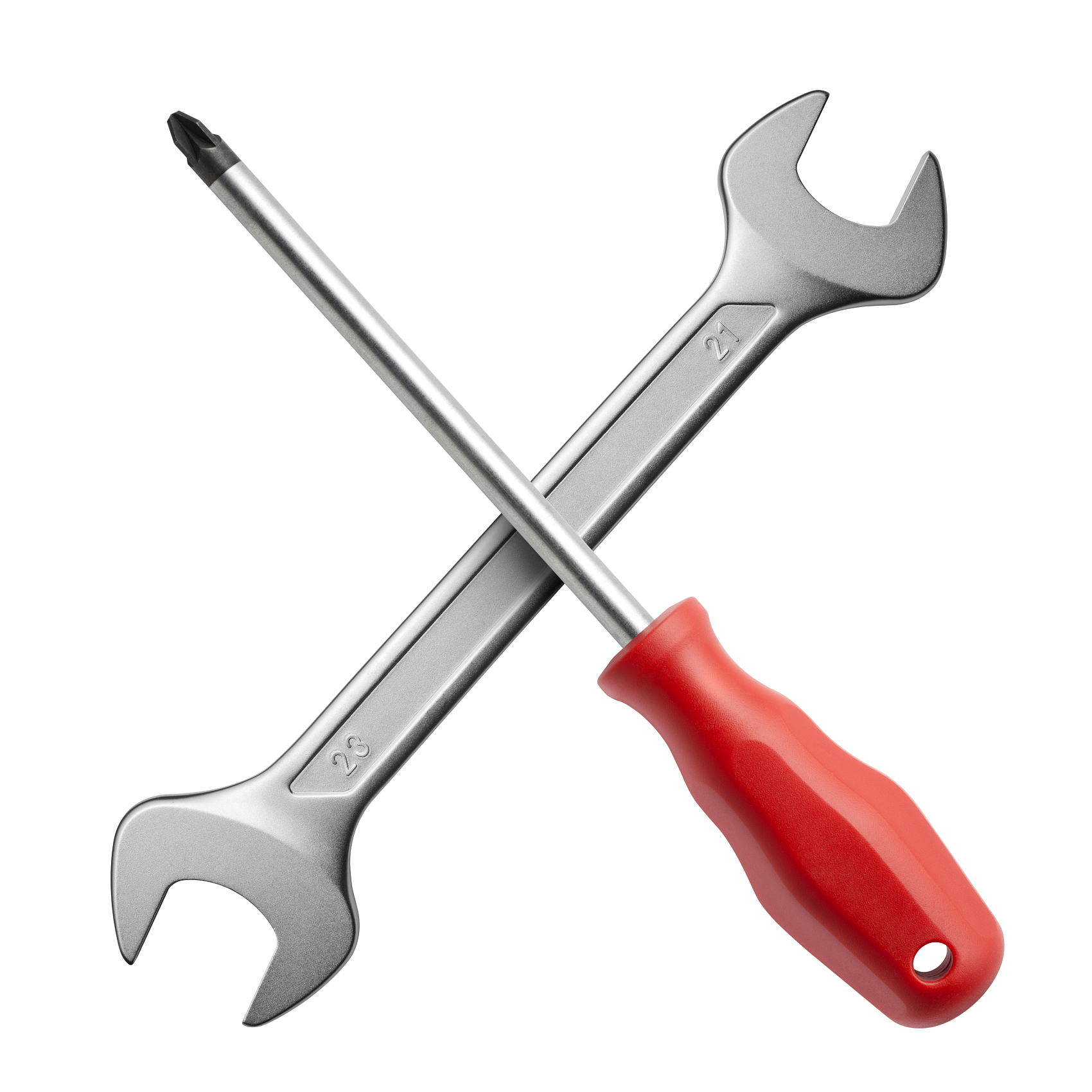


Although the AGREE II for TCM instrument does not provide thresholds for acceptable or unacceptable guidelines based on quality,a comparison of rigour scores across guidelines can provide the working groups with information to guide the selection process. For example, the working groups could decide on a cut-off point or rank the guidelines, once they see how the guidelines score on rigour (e.g., they may decide that any guideline scoring above 50% on the rigour dimension will be retained). Other options might be to keep all guidelines that score above the median score or all that score above the 60th percentile (5). It should be noted, however, that a poor score might not be sufficient in itself to eliminate a guideline at this stage.

The overall assessment item gives a general indication of whether or not the appraisers consider the guideline to be worth a more detailed assessment. For example, if all the appraisers state that they ‘would not recommend’ a particular guideline, that guideline could be eliminated from further consideration once the reasons for their decision have been discussed.

The expert groups may also decide to retain guidelines, based on other merits (e.g., excellent format or the presence of health questions not addressed in the higher quality guidelines). In addition, any member should be allowed to ask the expert groups to reinclude an eliminated guideline at anytime if a good case can be made for its reintroduction (9).

| 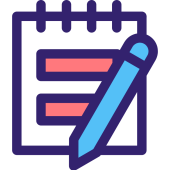 | **Illustration – Using the AGREE instrument to reduce a number of guidelines** |
| --- | --- |
| The guideline search found18cervical cancer TCM guidelines, which the expert groups and working groups felt were too many for the whole panel to review. Four appraisers who were part of the resource team completed the rigour dimension of the AGREE instrument for all 18guidelines. upon review, the chair and methodologist decided to keep all guidelines with an average rigour score greater than 40%for appraisal. They also decided to keep three guidelines that scored poorly on the rigour dimension, as they were guidelines created for the panel’s health care context and were all well known to panel members. The guidelines kept by the panel are represented by the dark bars on the graph.  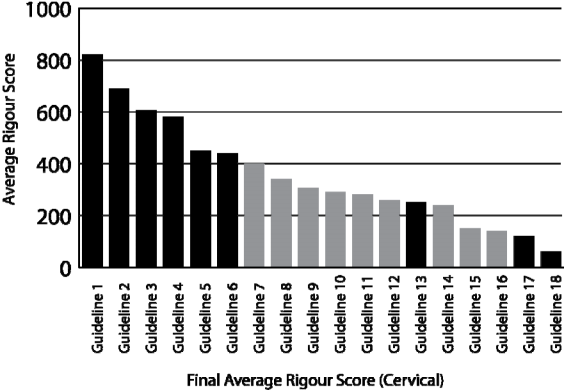 | |

| **Steps** | **Pruducts/**  **Deliverables** | **Skills and Organizational Requirements** | **Tools** |
| --- | --- | --- | --- |
| 11.Assess guideline quality  12. Assess guideline currency  13. Assess guideline content  14. Assess guideline consistency  (search and selection of studies, links between evidence and recommendations)  15. Assess acceptability/applicability of the recommendations | • AGREE scores  •Summary of currency evaluation  •Recommendations matrices  •Summary of search and selection  evaluation  •Summary of consistency between evidence, interpretations, and resulting recommendations  •Evaluation of applicability/ acceptability | • AGREE scores  • Summary of currency evaluation  •Recommendations matrices  • Summary of search and selection evaluation  • Summary of consistency between evidence, interpretations, and resulting recommendations  • Evaluation of applicability/ acceptability | [Tool 9 -](#bookmark177) AGREE II for TCM Instrument  [Tool 10](#bookmark178)– Quality evaluation of TCM diagnosis based on syndrome differentiation  [Tool 11 –](#bookmark179) Sample Currency Survey  [Tool 12](#bookmark180)– Sample  Recommendations Matrix [Tool 13](#bookmark181)– Table of Criteria for Assessing the Quality of Study Search and Selection  [Tool 14](#bookmark182)– Table for Recording Evaluations of Consistency between Evidence, Its Interpretations, and Recommendations  [Tool 15](#bookmark183)– Worksheet –Acceptability/Applicability |

The assessment of selected guidelines can take a multidimensional approach—an evaluation of the[quality,](#bookmark184) [currency,](#bookmark185) [content,](#bookmark186) [consistency,](#bookmark187) and [acceptability](#bookmark188)[/applicability o](#bookmark189)f the guideline recommendations. The evaluation of these different aspects will provide the basis for making an informed and transparent decision about which[source guidelines](#bookmark190) are relevant and for identifying which recommendations can be adapted. There is no evidence related to any of the assessments to support or refute thresholds standards. The panel needs to decide which assessments to prioritize or what they might accept as thresholds. The choice of assessments willbe based on decisions informed by elements such as the context, the health questions, the available evidence, and the resources of the group. Panels can be flexible in deciding which assessments will be undertaken and the order in which they will be implemented; however, the order decided upon by the panel should be outlined in the final document. Each of the assessments is described below.

**Step 11. Assess guideline quality**

**The AGREE II for TCM instrument**

*AGREE II for TCM: Tailoring it to Evaluating the Methodological Quality of TCM Clinical Practice Guidelines* (9) provides a framework for assessing the quality of TCM clinical practice guidelines. The 23 items in the AGREE II for TCM Instrument assess the methods used for developing the guideline and the quality of the reporting. An overall assessment item allows appraisers to make a judgement on the quality of the guideline as a whole, as to whether they would ‘strongly recommend,’ ‘recommend with alterations,’ ‘would not recommend,’ or are ‘unsure’ about recommending the guideline. The instrument does not assess the clinical content of the recommendations. The instructions in the introduction of the instrument should be read carefully before starting the appraisal.

[**Tool 9**](#bookmark175)–**AGREE II for TCM Scoring Forms**

**and Scoring Methods**


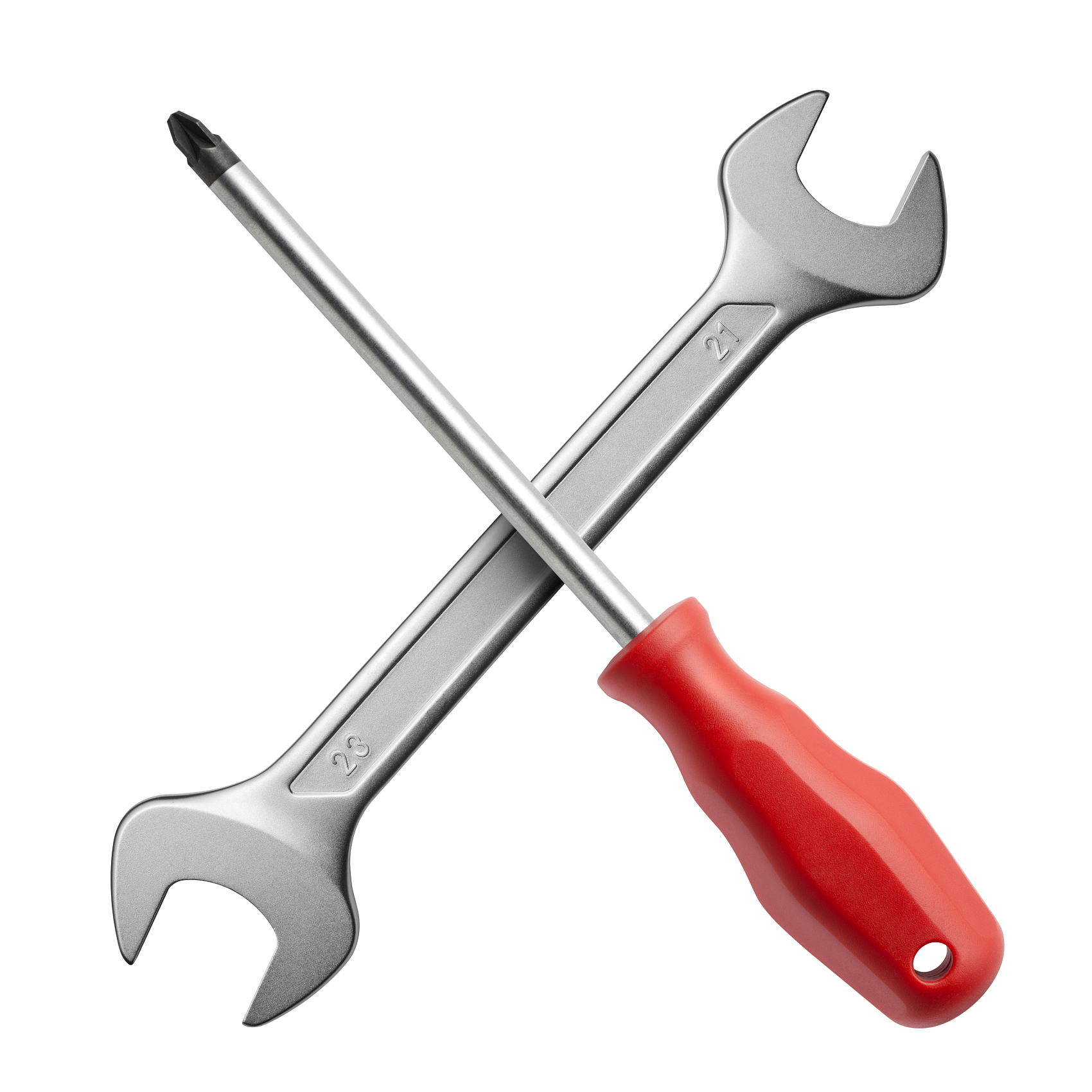


***Guideline Appraisal Training: Practice Set***

If the working group are unfamiliar with the AGREE II for TCM instrument, we recommend using one of the guidelines as a training exercise. The members would individually score the training guideline and would then have a short meeting to discuss any questions about the scoring, the dimensions, and soon. The AGREE II for TCM instrument uses a seven-point scale. Where users differ more than two point on any item, there should be a discussion to clarify discrepancies such as differing interpretations of the evaluation criteria or of the guidelines, different values, and soon. Often, the case arises where one member was unable to find a description in the guideline of the item in question and another member is able to point out the location in the text. The training exercise provides members with practice in using the instrument itself and also some indication of how guidelines might be organized.

***Main appraisal***

Each panel member should receive the AGREE II for TCM instrument, a copy of the selected guidelines, and any supporting material related to the guidelines.

If possible, there are benefits to having all members of the panel appraise the guidelines to be discussed (2), including the following:

• The appraisal gives all members an in-depth understanding of the content of each guideline and, therefore, generates a more informed discussion.

• It has an educative value as panel members will gain greater awareness of various aspects of guideline structure and content, including what constitutes a good quality document.

• A review of the quality scores can identify where there is a lack of agreement on scoring specific items and will become part of the consensus discussion.

• Overall quality scores from all members can increase reliability when ranking the guidelines.

It maybe impractical from a resource or time perspective to have all working group members rate all of the guidelines.With respect to improving the reliability of the AGREE II for TCM instrument, each guideline should be appraised by at least two and preferably four appraisers.

[**Tool 10**](#bookmark192)– **Quality evaluation of TCM diagnosis based**

**on syndrome differentiation**


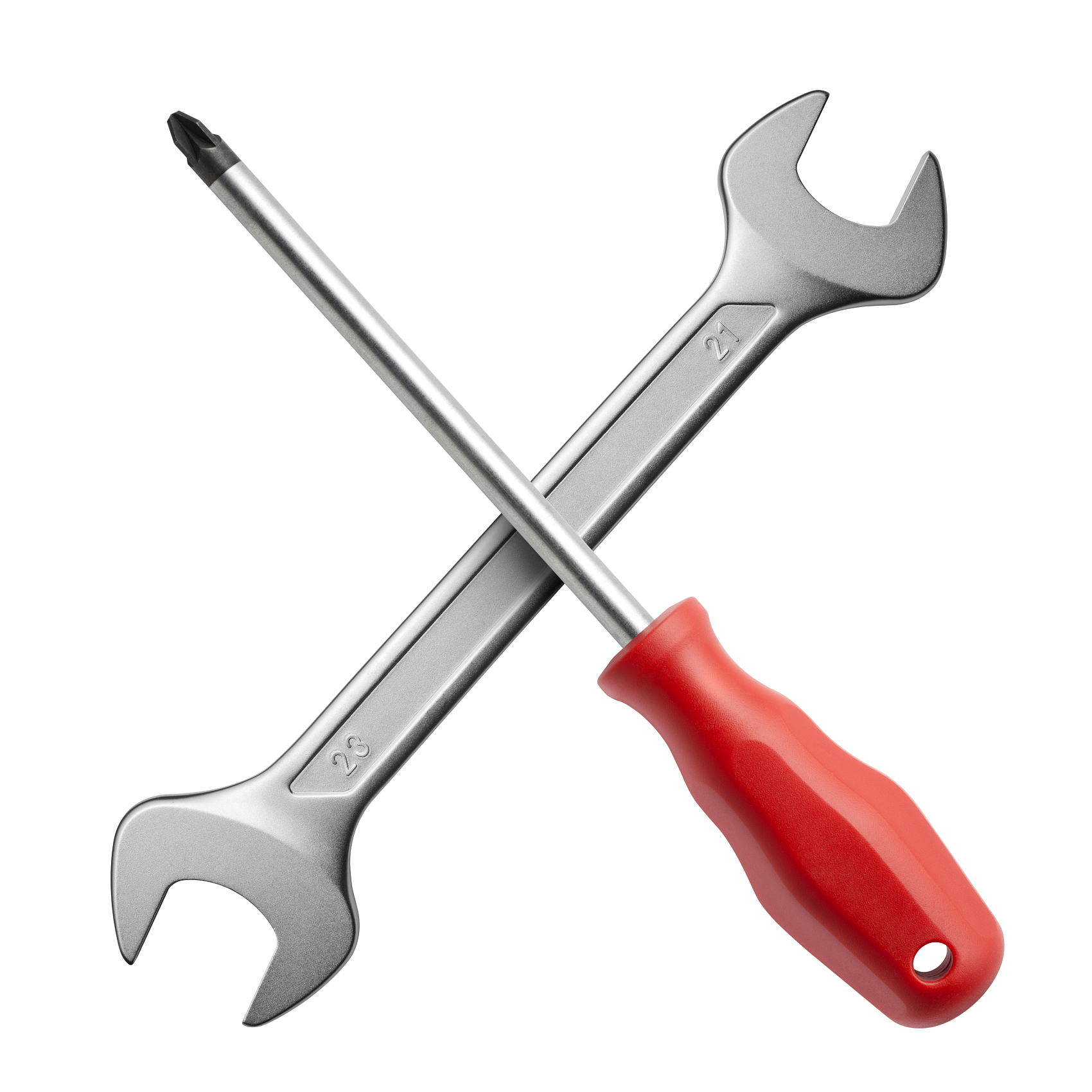


***How the scores can be used***

The AGREE scores provide a sense of the quality of some aspects of the guideline and how well they were reported. They can be used as one element in the decision-making process around whether or not to adapt a specific guideline. These scores are helpful in decision making, particularly the domain “*Rigour of Development*,” for instance, if the panel has decided to consider only rigorously developed guidelines. The panel might also be interested in considering guidelines with other merits such as an ideal format or the inclusion of recommendations highly relevant to their local condition and that other guidelines do not include. A poor AGREE score may not be sufficient in itself for eliminating a guideline.

The raw AGREE scores can be used to show rater agreement and disagreement on the various items of the AGREE instrument. All scores of 1 or 2 (strongly disagree or disagree) can be highlighted in one colour or texture, and all scores of 6 or 7 (agree or strongly agree) can be highlighted in another. AGREE items that have equal amounts of each colour and/or texture would be areas for discussion as that situation means that one half of the panel differs from should beheld to clarify the source of the differences. As well, [intraclass correlations (](#bookmark193)ICC) (10) could be calculated to give a numerical value of appraiser variability.

The graphical representation of how guidelines compare on the various AGREE dimensions provides a simple and clear measure of comparison. Large differences in the scores for the same dimension across different guidelines can act as a discussion point.

|  | **Illustration – Graphical representation of the AGREE**  **domain clarity and presentation scores for cervical cancer screening guidelines** | |
| --- | --- | --- |
| **90**  **80**  **70**  **60**  **50**  **40**  **30**  **20**  **10**  **0** |  | \| 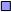 **Clarity and**  **Presentation** \| \| --- \| |
|  | CPG 1 CPG 2 CPG 3 CPG 4 CPG 5 CPG 6 |  |

**Step 12. Assess guideline currency**

Research on the validity of practice guidelines has shown that the evidence supporting guidelines in fields that are rapidly evolving maybe outdated in as little as three years, depending on the research activity in the field (11,12). As a result, it is important to assess whether the guidelines are adequately current for the adaptation process. The publication date of the guideline, or the dates/period covered by the literature, should be reviewed to ascertain whether the most current evidence has been included (6) Some developers publish this information in the guideline itself or on their Websites, for example, the Cancer Care Ontario (www.cancercare.on.ca) and the Scottish Intercollegiate Guidelines Network (www.sign.ac.uk).

Because the evidence of ancient Chinese medicine books has a significant impact on the formation of recommended guidelines, when determining the timeliness requirements, the time span of evidence in traditional Chinese medicine guidelines should be determined based on the overall release and update characteristics of the guidelines in this field. The review of whether the guidelines include ancient book evidence and whether they systematically trace relevant ancient Chinese medicine book evidence since the record of a certain disease ensures the systematicity and integrity of the evidence in traditional Chinese medicine guidelines.

If you suspect that a guideline is out of date, there are following updating options:

• Consult with an expert well versed in the field and conduct a rapid review of the literature.

• Contact the guideline developer for further information on currency. A short survey of the guideline developers can ascertain whether there is a more recent version of the guideline, whether the developer intends to update the guideline in the future, and whether the developer is aware of any new evidence that might affect the guideline recommendations (9).

• Perform a literature search of Websites most likely to provide up-to-date information, in particular, systematic reviews, and contact experts in the field regarding the state of

knowledge in a content area.

• Verify whether alerts on an intervention have been released by a monitoring agency such as the State Drug Administration (SDA) .

If the[source guidelines o](#bookmark194)r guidelines are of good quality but the literature is not up-to-date, the literature or evidence must be updated.

If the panel learns that a guideline developer is aware of new evidence that could affect the recommendations or that the developer will be changing a guideline’s [recommendations](#bookmark195) substantially, based on new evidence, the panel will need to make some decisions about whether or not to use the guideline in the adaptation process, and will need to document these decisions. A number of the recommendations might possibly be unaffected by the new evidence and portions of the guideline could be retained for adaptation. The panel, however, will need to decide whether to update any recommendations affected by the new evidence, write them *de novo*, or wait for the release of the updated guideline.

[**Tool 11 –**](#bookmark196) **Sample Currency Survey**


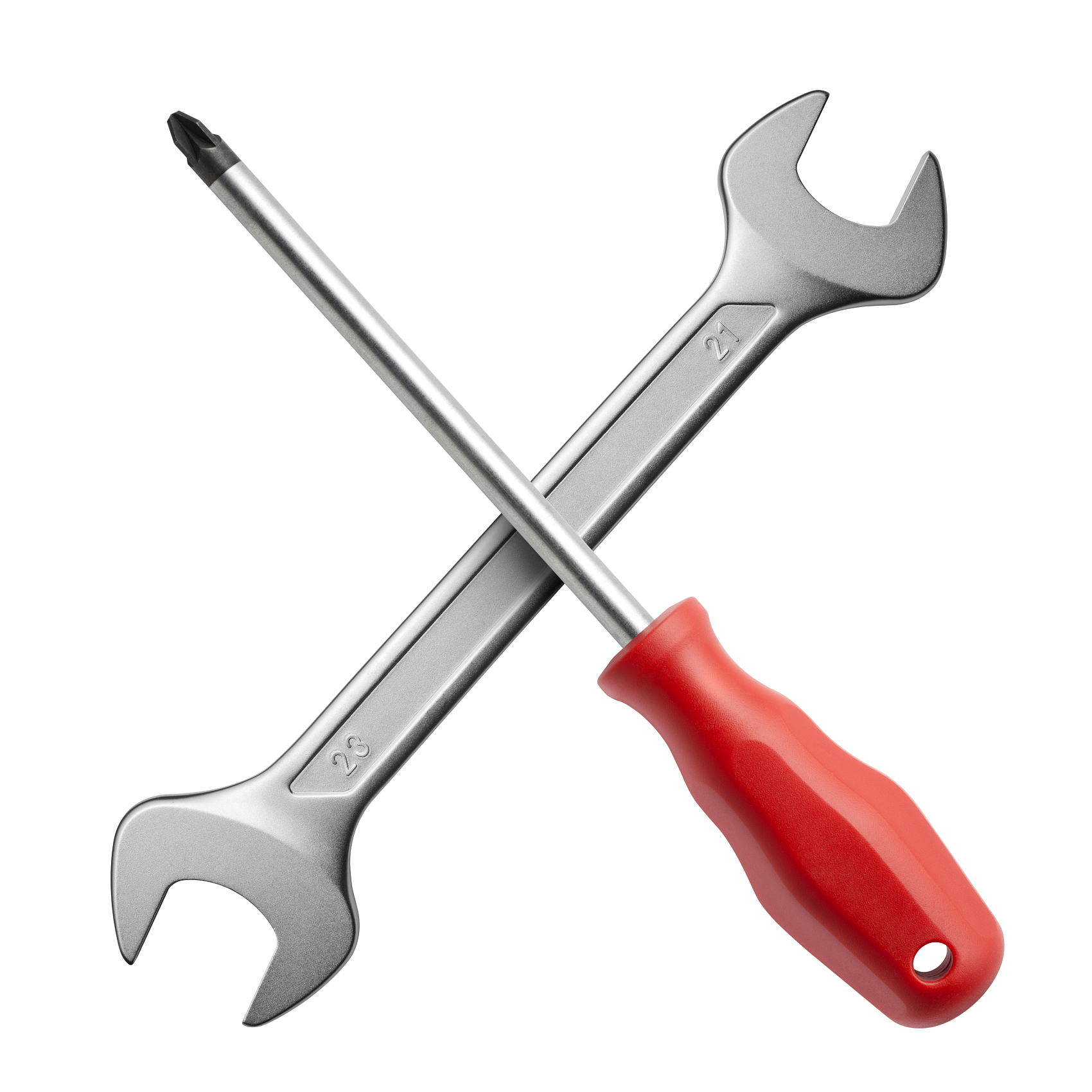


**Step 13. Assess the recommendations of guideline**

[Matrices are tables of recommendations d](#bookmark197)rawn from the guidelines under review, although they also might include recommendations from systematic reviews or health technology assessments. We recommend that a TCM or Integrative Medicine clinician who specializes in the topic produce or review the matrices to ensure that no recommendation has been taken out of context. Matrices are most useful in those applications where more than one[source guideline i](#bookmark198)sunder consideration (1,2).

| 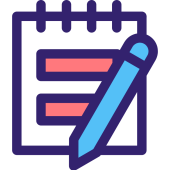 | **Illustration – Results of the currency survey** |
| --- | --- |
| Seven TCM/Integrated Traditional Chinese Medicine (ITCM) guidelines for psoriasis vulgaris, distributed in the following years: 1994, 2009, 2010, 2012, 2013, 2014, and 2023. The Adaptation Organizing Committee believes that although the 1994 guidelines have not been updated for nearly two decades, they are industry standards with high authority and can be retained for further consideration, taking into account the specificity of Chinese medicine and the fact that ancient texts are an important source of evidence in the development process. | |

The matrices can be used by the panel for decision making in a number of ways:

• Where similar recommendations from various guidelines are grouped together, recommendations can be easily compared to see whether they are similar ordifferent, and if different, how they differ.

• The matrices help the group identify all recommendations with strong evidence.

• The matrices help the panel compare wording of recommendations.

• The matrices can provide a basis for a discussion about the clinical relevance of each recommendation.

The recommendations matrices can be presented in two different formats, 1) recommendations grouped by guideline and 2) recommendations grouped by similarity (e.g., all the recommendations on TCM external treatment methods are grouped together).

***Create recommendations matrices***

The matrices list the recommendations down the left column and the name of the [source](#bookmark199) [guideline a](#bookmark200)cross the top. Guidelines could be ordered across the top by date, for example, the most recent in the first column, the second most recent in the second, and so on. They may also be ordered by quality scores on the AGREE II for TCM instrument, based on particular dimensions. For example, the guideline rating highest on the rigour dimension might be listed first (along with its date) and so on. Other information provided could be how each guideline rated on the overall assessment of the AGREE II for TCM instrument (e.g., how many rated the guideline as ‘strongly recommend,’ how many as ‘recommend with modifications,’ how many as ‘would not recommend,’ and how many as ‘unsure’) (1,2).

The levels of evidence associated with the recommendations can be placed within each cell. The difficulty with using levels of evidence is that there is no common classification system, and thus, one must either devise some broad generic system and reclassify each level from the source guideline or provide a guide as to each developer’s definitions of their levels of evidence. Another difficulty is that some developers do not attach levels of evidence to their recommendations. However, if the panel has already completed the assessments related to guideline consistency (Tools 13 and 14), then they might reclassify the levels of evidence for each recommendation, using their own system. Instead of using the levels of evidence, the actual type of study data supporting the recommendation could be listed (e.g., six randomized controlled trials or expert opinion).

Another option would be to put in the evaluations of consistency (as described in the section “Assess guideline consistency” below) associated with each recommendation. If electronic matrices are created,a hyperlink could take the reader to a summary of the evidence.

[**Tool 1**](#bookmark201)**2** - **Sample Recommendations Matrices**


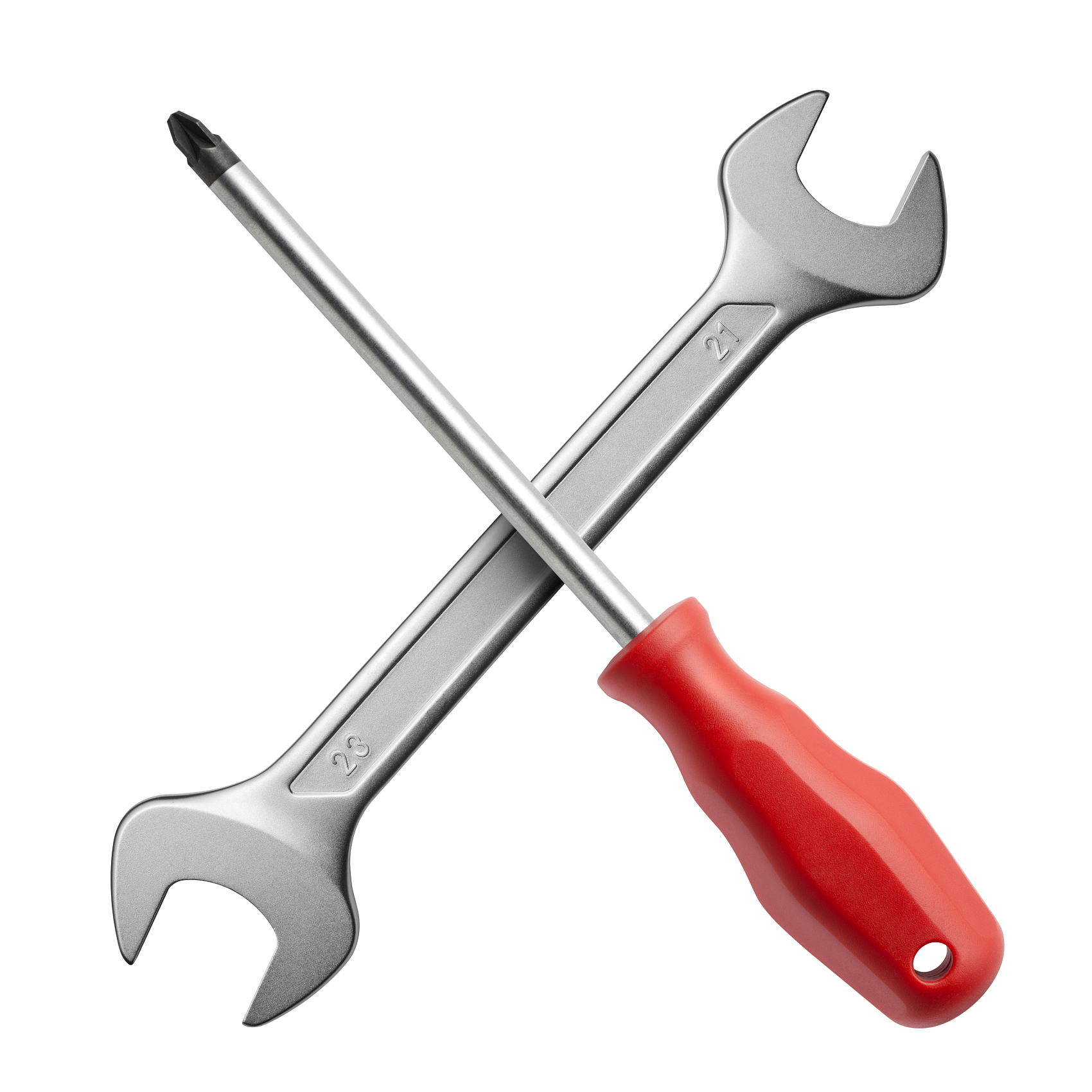


| 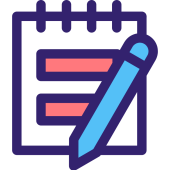**Illustration – A portion of the recommendation matrix for recommendations for blood heat syndrome in TCM diagnosis and treatment of psoriasis vulgaris and integrated traditional Chinese and western medicine diagnosis and treatment**  (Guidelines arranged from left to right by date. Rigour scores, overall quality assessment ratings, and levels of evidence included.) | | | | | | | |
| --- | --- | --- | --- | --- | --- | --- | --- |
| **Recommendation on the treatment of blood heat syndrome – Grouped by**  **Recommendation** | **Guideline 2**  **Guideline 1**  **2003** | **Guideline 2**  **2008999**  **2003** | **Guideline 2**  **Guideline 3**  **2002** | **Guideline 4**  **199812** | **Guideline 5**  **1995** | **Guideline 6**  **1993** |  |
| **AGREE II for TCM Rigour scores** | 67.62 | 80.95 | 70.39 | 42.68 | 54.78 | 32.22 |  |
| **Overall quality assessment** | Strongly  recommend  (6 raters) | Strongly  recommend  (7 raters) | Recommend  with  Alterations  (5 raters) | Not  recommend  (2 raters) | Recommend  with  alterations  (4 raters)  Recommend | Not recommend  (2rater) |  |
| **Diagnostic criteria for blood heat syndrome** | Conclusions derived from literature studies | Conclusions derived from expert consensus | Conclusions derived from literature studies, expert consensus |  |  |  |  |
| **TCM diagnosis and treatment prescription** | Rhinoceros and rehmannia decoction (Evidence Level II) |  | Cold blood detoxification decoction (Evidence Level II) |  |  |  |  |
| **Prepared prescription (Chinese medicine)** |  |  |  |  |  |  |  |
| *** Levels of evidence listed by the guideline developers were reclassified into a system for comparison within the matrix.** | | | | | | | |

**Step 14. Assess guideline consistency**

The assessment of the consistency of the guideline includes the following four evaluations:

• Search strategy and selection of evidence supporting the [recommendations](#bookmark202)

• Consistency between the selected evidence and how developers summarize and interpret this evidence

• Consistency between the interpretation of the evidence and the recommendations

• Consistency between the quality of the evidence and the strength of the recommendation (in particular, whether there are sufficiently persuasive reasons when low-quality evidence gives a strong recommendation).

This process requires the involvement of clinical staff or methodologists with a background in TCM knowledge.

In performing these evaluations, the panel will need to review the [source guidelines t](#bookmark203)horoughly. The evaluations will help identify any recommendations in the source guidelines that do not follow directly from the evidence; panel members can then determine whether they will eliminate those recommendations from further consideration.

The evaluations are time consuming, require a thorough review of each source guideline by individuals with methodological and TCM clinical expertise, and may require the gathering of original evidence supporting the interpretations and recommendations in the guideline. However, they provide appraisers with a sense of confidence that the source guideline was developed rigorously, and that there is consistency between the evidence, its interpretation, and the recommendations.

***Evaluate search strategy and selection of evidence***

The type and quality of the evidence on which recommendations are based can vary, depending on the exact health question addressed and when and how the search for evidence was

performed. The period covered by the search and the use of inclusion/exclusion criteria such as language can often explain this variation. An evaluation of the source guideline’s search strategy and the selection of evidence used to support the recommendations will determine whether the guideline developers systematically searched for and selected relevant evidence and systematically extracted relevant data. The evaluation should include assessing the relevance and exhaustiveness of the databases searched, the search strategies used (e.g., keywords, dates, and languages), the methods and criteria used to select the references, and how many references were identified, included and excluded.

[**Tool 13 –**](#bookmark204) **Table of Criteria for Assessing the**

**Quality of Study Search and Selection**


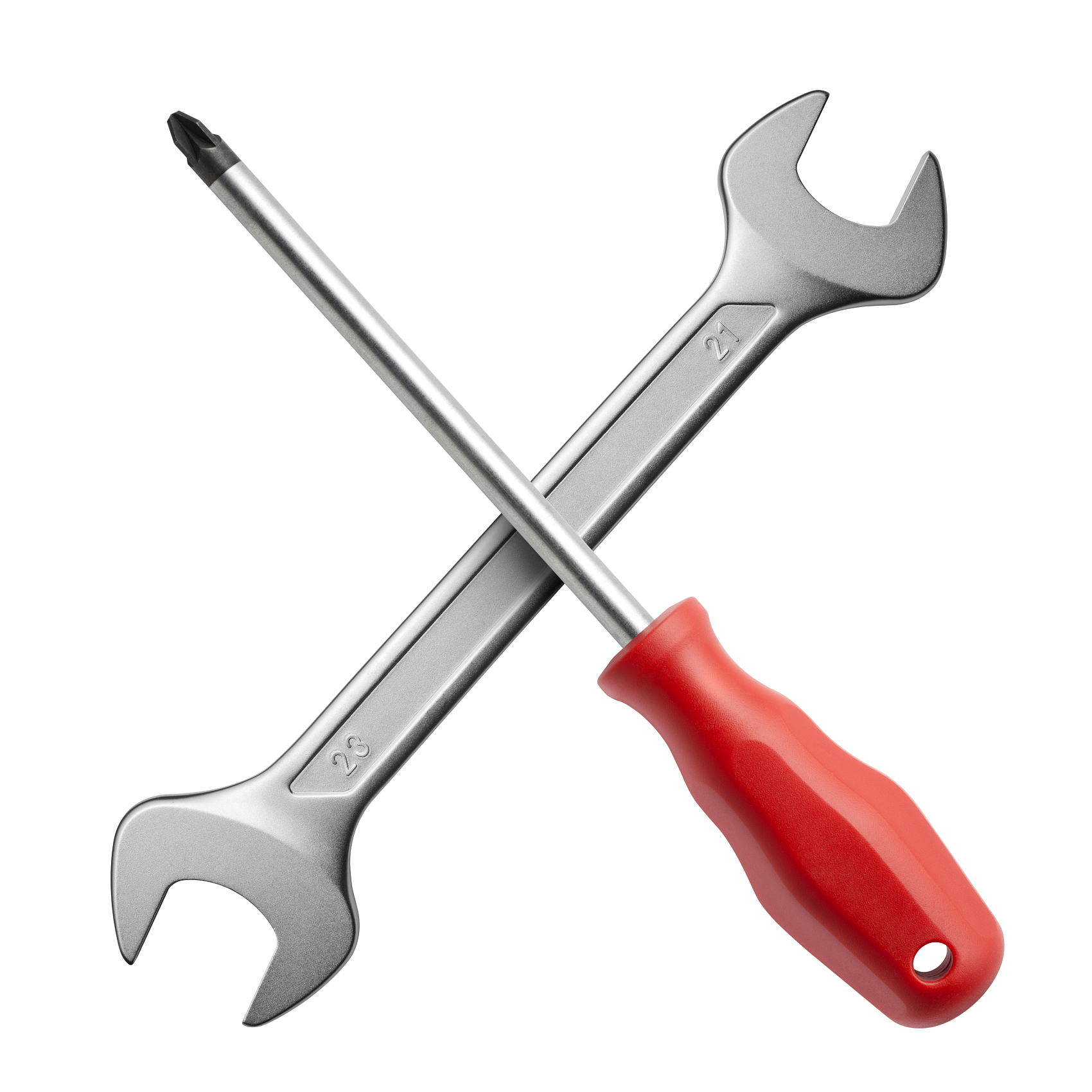


***Evaluate consistency between selected evidence, its interpretation, and resulting recommendations***

An evidence-based guideline consists of three main components, the evidence generated via the systematic review on which the source guideline is based, the interpretation of that evidence within the healthcare context and the developers’ experience, and the guideline recommendations that take into account the local situation and values (13). An evaluation of the consistency between these three components examines the quantity and quality of the selected evidence as well as the consistency of results and determines whether the interpretation of the evidence flows from the selected evidence and whether the recommendations are also consistent with the selected evidence. This evaluation will be facilitated by having access to the [evidence](#bookmark205) [tables.](#bookmark206) If these are not included in the published guideline, we recommend that the developers of the source guideline(s) be contacted. With respect to the recommendations, in the case where evidence is weak or non-existent, the basis for the resulting recommendation should be explicitly indicated in the [source guideline (](#bookmark207)e.g., based on expert consensus by the guideline development panel).

There area number of questions to be considered in conducting this evaluation:

• Are the consistency and clinical relevance of primary study results reported or discussed?

• Is the clinical and methodological heterogeneity of studies reported or discussed?

• Were the recommendations supported by the conclusions of the critical appraisal of the studies? If not, are there other reasons explicitly presented?

• Is the method for indicating the level of evidence adequately described?

• Is this method used correctly,i.e. is the level of evidence attributed to the recommendation justified?

• Were the patients and interventions in the studies analysed judged to be sufficiently comparable to those targeted by the recommendations?

• Has the balance between risks and benefits been correctly taken into consideration?

• Was a formal process used to define the recommendations?

[**Tool 14 –**](#bookmark208) **Table for Recording Evaluations of Consistency between**

**Evidence, Its Interpretation and Recommendations**


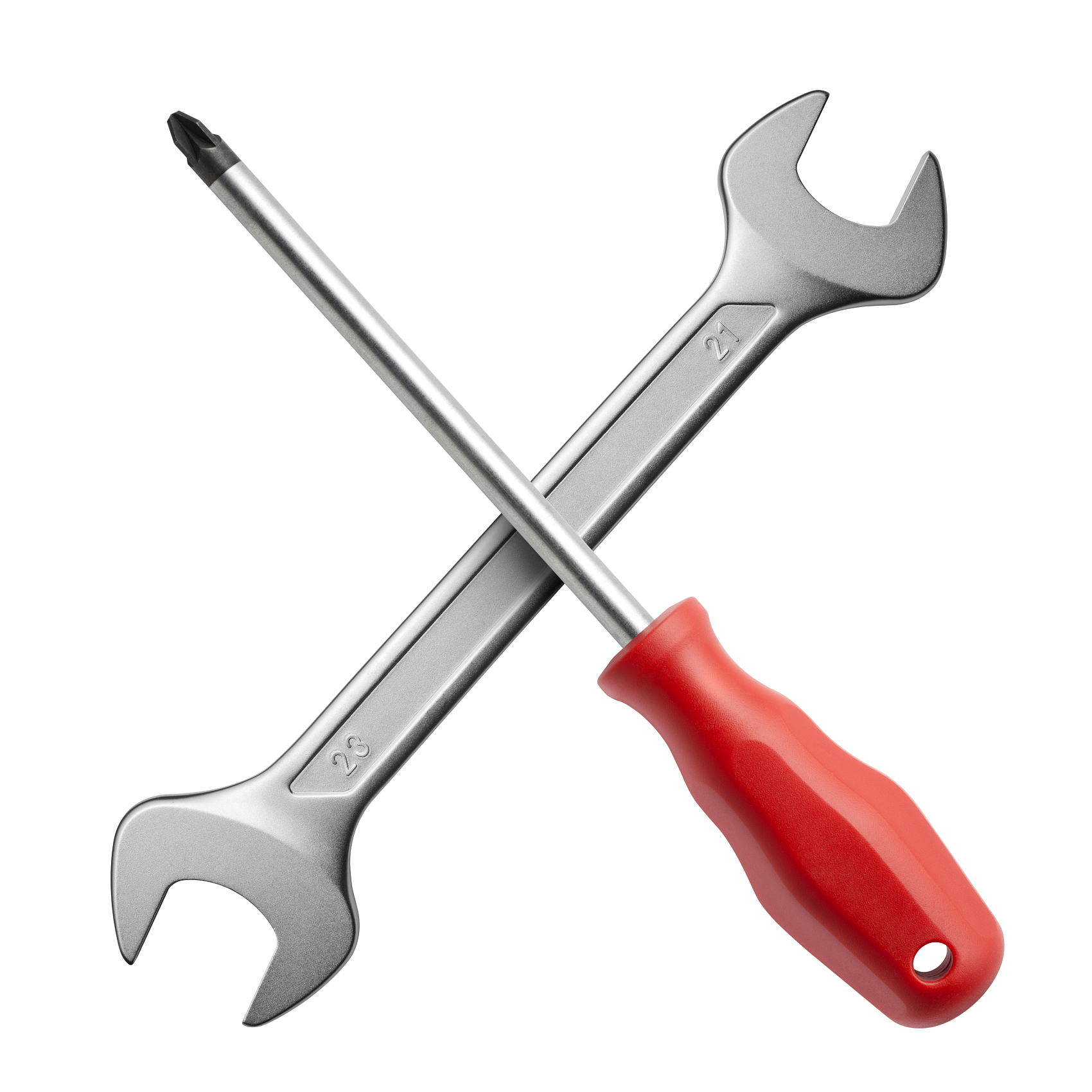


**Step 15. Assess acceptability and applicability of the recommendations**

There area number of terms that can be used to describe whether a recommendation will be used in practice. [Acceptability,](#bookmark209) feasibility, implementability, and [applicability a](#bookmark210)ll have slightly different meanings but in essence describe 1) whether the recommendation should put it into practice (acceptability) and 2) whether an organization or group is able to put the recommendation into practice (applicability).

The applicability of a guideline’s[recommendations i](#bookmark211)n the target context and the degree to which a guideline will need adaptation depends on the differences in the cultural and organizational context, including the availability of health services, expertise, and resources and the organization of health services, as well as population characteristics, beliefs, and value judgments. These context variables are particularly important when adapting guidelines for culturally sensitive interventions or technological innovations. Special considerations for the TCM guidelines are the local acceptance of TCM and Chinese medicine, as well as invasive operations such as acupuncture; and the coverage of TCM-related treatment techniques by the local health care insurance system.

It is recommended to refer to AGREE-REX instrument (14), the contents of Chapter 14 of the *Manual for the Development of Integrated Chinese and Western Medicine Treatment Guidelines* (4) edited by Lu Chuanjian et al. It was used to assess the applicability of a recommendation.

Assessing whether a recommendation is acceptable and/or applicable or not is done by discussing each recommendation in light of the following questions:

• Does the population described for eligibility match the population to which the recommendation is targeted in the local setting (acceptable)?

• Does the intervention meet patient views and preferences in the context of use (acceptable)?

• Are the intervention and/or equipment available in the context of use (applicable)?

• Is the necessary expertise (knowledge and skills) available in the context of use (applicable)?

• Are there any constraints, organisational barriers, legislation, policies, and/or resources in the healthcare setting of use that would impede the [implementation o](#bookmark212)f the recommendation (applicable)?

• Is the recommendation compatible with the culture and values in the setting where it is to be used (acceptable and applicable)?

• Does the benefit to be gained from implementing this recommendation make it worth implementing (acceptable)?

These questions can be proposed to the panel by the chair as each recommendation is being considered. Another way to address these questions is through an assessment form. Panel members might be asked to answer these questions at the same time as they are appraising the guidelines using the AGREE instrument. Results could then be fed back to the panel at the beginning of the meeting.

[**Tool 15**](#bookmark213)  **– Worksheet – Acceptability/applicability**


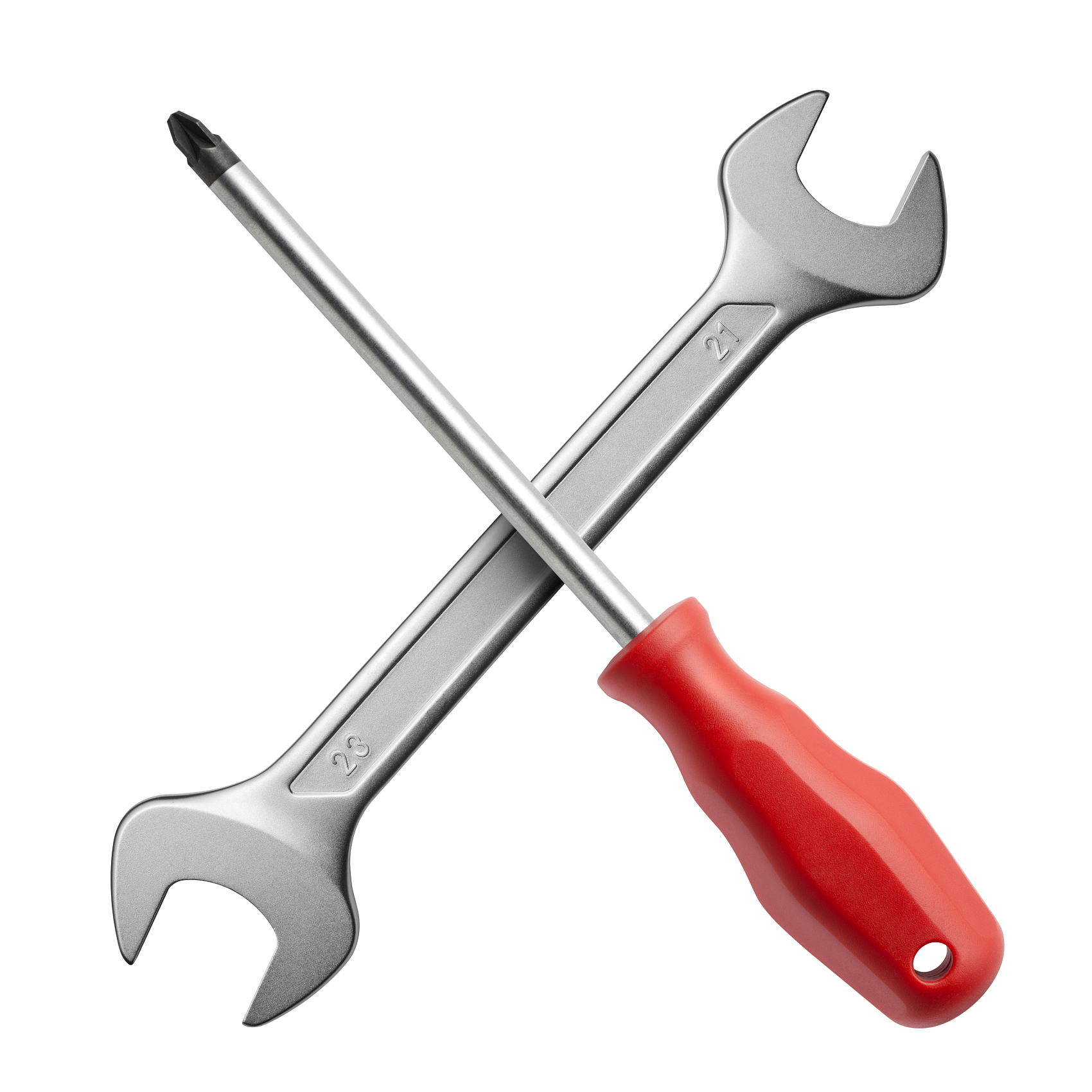


| **Steps** | **Pruducts/**  **Deliverables** | **Skills and Organizational Requirements** | **Tools** |
| --- | --- | --- | --- |
| 16.Review assessments to aid in decision making  17. Select between guidelines and recommendations to create an adapted guideline | • Decision made on the content of the final document | TCM clinical expertise  Methodological expertise  Facilitation skills (Chair) | See Table for a list of all resources available to  the panel |

**Step 16. Review assessments**

The results of the assessment module provide an explicit basis for informed and transparent decision making around the selection and modifications of [source guidelines.](#bookmark214) At the panel meeting, members will be presented with a number of documents that summarize the results of the assessment module (see Table). Some of the assessments relate to the consistency of the source evidence with the interpretations and the recommendations, some relate to the guideline as a whole, and some relate to the recommendations.

**Table. Available assessments and their possible use by the panel**

| **Assessments Related to Quality** | **Possible Use** |
| --- | --- |
| Overall AGREE II for TCM assessment | Can be used as a starting point for elimination of those guidelines that most members “would not recommend.” |
| Raw AGREE II for TCM scores | Used to assess rater agreement and ensure that the panelists’ scores are reliable. Can be used to show where there are major differences among panel  members on various items of the dimensions of the AGREE instrument. Can be used to promote  consensus by highlighting areas of disagreement in perceptions of the guideline. |
| Summary AGREE II for TCM dimension graphs | Can be used to show how one guideline rates on  each of the six AGREE domains or how all of the guidelines compare on each of the various AGREE domains. |
| Quality evaluation of TCM diagnosis based on syndrome differentiation [(Tool 10)](#bookmark215) | Can be used to evaluate the reliability of diagnostic criteria for traditional Chinese medicine syndromes. |
| Results of the currency assessment [(Tool 11)](#bookmark215) | Can be used to eliminate any guidelines that are out of date or that will soon undergo a major revision.  Can also be used to define where updates are needed. |
| **Assessments Related to Quality** | **Possible Use** |
| Recommendations matrices [(Tool 12)](#bookmark216) | Can be used to easily compare recommendations from all of the potential guidelines with respect to content and wording and level of evidence, if  included. |
| Supporting material (e.g., systematic reviews, health technology assessments, articles) | Can be used to provide more information on certain topic areas, to fill in gaps not covered by  recommendations, to update recommendations, or to confirm the accuracy of evidence supporting the recommendations. |
| Results of the evaluation of the search selection of evidence [(Tool13)](#bookmark217) | Provides an indication for each guideline of the  comprehensiveness of the search strategy and the  evidence selected. |
| Results of the evaluation of consistency between evidence and its interpretation and between the interpretation and recommendations [(Tool 14)](#bookmark218) | Provides an indication of whether there are  inconsistencies with the guideline developers’  interpretation of the evidence and its translation  into recommendations within a guideline or  between guidelines. |
| Results of the applicability evaluation [(Tool 15)](#bookmark219) | Can be used to decide if the recommendations are applicable, can be implemented in the user’s  context, and are worth implementing |

**Step 17. Select between guidelines and recommendations to create an** **adapted guideline**

The expert group should assist the working group in following the consensus process which they had previously decided upon. The steps followed incoming to group consensus, or not reaching any consensus, **must** be recorded. The expert group and the working group and/or panel need to pay careful attention to any new evidence brought to the panel during the discussion to determine if any of the [recommendations](#bookmark220) are affected by this evidence. Any modifications to the recommendations must be carefully documented and the evidence supporting the modification provided, along with supporting references. This is a meeting best held face-to-face. Good facilitation skills are needed by the chair to ensure that all members have an opportunity to present their views.

Decision making and selection occurs around the following five options:

**1) REJECT the whole guideline:** After reviewing all of the assessments, the expert group decides to reject the complete guideline. The decision should be based on how the panel weighs the assessments (e.g., poor AGREE II for TCM scores, guideline is out-of-date, or the recommendations do not apply to the panel’s context).

**2) ACCEPT a whole guideline and all of its recommendations**: After reviewing all of the assessments, the panel accepts the guideline as is.

**3) ACCEPT the evidence summary of the guideline:** After reviewing all of the assessments, the panel decides to accept the description of the evidence (or parts of it) but to reject the interpretation of the evidence and the recommendations.

**4) ACCEPT specific recommendations:** After reviewing the recommendations from the guideline or guidelines, the panel decides which recommendations to accept and which to reject (e.g., those recommendations needing major modification would be

rejected), which maybe from one or more guidelines.

**5) MODIFY specific recommendations:** After reviewing the recommendations

from the guideline or guidelines, the panel decides which are acceptable but need to be modified (e.g., new data maybe added to the original recommendation or the wording might be changed to better reflect the panel’s context).

**Caution: When formulating recommendations, emphasis should be placed on how to translate complex interventions based on "syndrome differentiation and treatment" (such as rheumatic fever accumulation syndrome, blood deficiency and wind-dryness syndrome) into clear recommendations that healthcare practitioners in the target country or region can understand and patients can accept, while fully considering the locally available herbal products and acupuncture services.**

**Care must always betaken when modifying existing guidelines and/or recommendations not to change the recommendations to such an extent that they are no longer in keeping with the evidence upon which they should be based.**

Based on the above decisions, the panel can create an adapted guideline acceptable for their context that addresses all of their health questions.

| 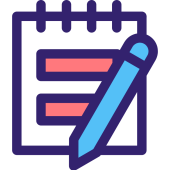  **Illustration – Decision making process followed by the cervical cancer screening panel**   \| **Process** \| **Action** \| \| --- \| --- \| \| 1. Panel decides to begin by seeing if they can eliminate guidelines that members would not recommend. Reviewed overall assessment scores – ‘strongly recommend’ category. Began with those that had ‘0’ in strongly recommend category. Those who did recommend ‘with alterations’ were asked to discuss their decisions. \| Guideline 5 and 7 are eliminated – not screening guidelines \| \| 2. Continue to use overall assessment scores to look at next poorest scoring guideline – ‘1’ strongly recommend, ‘2’ recommend with alterations, ‘3’ would not recommend, ‘4’ unsure. Asked member who ‘strongly recommended’ to discuss decision. \| Guideline 4 is eliminated - outdated \| \| 3. Review information from the currency survey \| Panel notes also that Guideline 3 is outdated – removed from further consideration \| \| 4. Begin discussion of top three choices (*based on their AGREE scores).* \| Temporarily put aside Guideline 6 as group doesn’t have enough information about  developer and conflict of interest \| \| 5. Decide to look at the individual recommendations of the top three guidelines. Discuss Guideline 1. \| Accept all five recommendations of Guideline 1 after discussion. \| \| 6. Discuss Guideline 2. \| Panel decides that they cannot agree with annual screening, did not find rationale for why 70 years was selected as a stopping age for  screening. \| \| 7. Go on to discuss Guideline 6. Importance of guideline needing to address practice reality is discussed. \| Panel feels Guideline 6 is too lengthy for busy family physicians and merely repackages recommendations from other developers. Also concern that they are sponsored by US State health plans. \| \| 8. Panel decides to go through Guideline 1 to see if can accept in entirety. \| Consensus to accept as is and group provides rationale. \| \| 1. Decides to look at Guideline 2 and Guideline 6 to see if they can be accepted in entirety as well. \| Decide that cannot accept either as is. \| \| 1. Discussion on target population. \| Group decides that source guidelines only cover average-risk population. Decides to ‘park’ high- risk population – need more information and/or comprehensive list of relevant guidelines. \| \| 11. Consensus achieved. \| Panel agrees to accept Guideline 1 in its current form. \| |
| --- | --- | --- | --- | --- | --- | --- | --- | --- | --- | --- | --- | --- | --- | --- | --- | --- | --- | --- | --- | --- | --- | --- | --- | --- |

| **Steps** | **Products/**  **Deliverables** | **Skills and**  **Organizational Requirements** | **Tools** |
| --- | --- | --- | --- |
| 18.Prepare a document that respects the needs of the end users and provides a detailed transparent explanation of the process | • Draft guideline document | Knowledge of clinical practice and local Context  Editorial skills  Design skills | [Tool 1](#bookmark221)6– Checklist of Adapted Guideline Content  [Tool 1](#bookmark221)7– Checklist of the RIGHT Extension Statement for traditional Chinese medicine (RIGHT-TCM) |

**Step 18. Prepare draft adapted guideline**

Once the panel has reached a decision on the content of the adapted guideline, the source guideline developer should be contacted to obtain more information on the guidelines and and a draft document will be produced that should include details on the process followed. A suggested template for the format of the guideline is presented in [Tool 16.](#bookmark222) Alternatively, an adapted version of the clinical practice guidelines for TCM can be drafted with reference to the RIGHT TCM Extended Version (RIGHT-TCM) (15) and RIGHT-Ad@pt (16) lists.


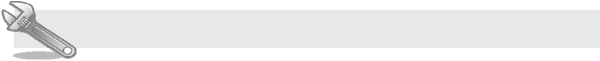


[**Tool 16**](#bookmark223)**– Checklist of Adapted Guideline Content**

The template includes the following sections:

1. Overview material:

• structured abstract that includes the guideline’s release date and print and electronic sources

• name and institutional affiliation of adaptation panel

2. Introduction and background

3. Scope and purpose

4. Target audience of the guideline

5. Target population

6. Health questions

7. Recommendations:

• risks and benefits associated with the recommendations

• specific circumstances under which to perform recommendations

• strength of recommendations based on stated recommendation grading criteria (if used)

8. Supporting evidence and information for the recommendations:

• panel rationale behind the recommendations

• presentation of additional evidence and/or the results of the updating process

• how and why existing recommendations were modified

9. External Review and Consultation Process (to be discussed in next section)

• who was asked to review the guideline

• what process was followed

• discussion of feedback and what was incorporated into the final document

10. Plan for scheduled review and update (to be discussed in next section)

11. Algorithm or summary document

12. Implementation considerations

13. Glossary (for unfamiliar terms)

14. References of all material used in creating the guideline

15. Acknowledgment of source guideline developers and permission granted (where necessary)

16. List of panel members and their credentials, declaration of conflicts of interest

17. List of funding source(s)

18. Appendix describing adaptation process:

• guideline search and retrieval including the list of guidelines identified and whether they were included or excluded and why

• guideline assessment including which assessments were undertaken and in which order, and a summary of results for each assessment (including AGREE II for TCM domain scores)

• decision process followed by panel

• results and decisions of each evaluation

[**Tool 1**](#bookmark223)**7– Checklist of the RIGHT Extension Statement for**

**traditional Chinese medicine (RIGHT-TCM)**


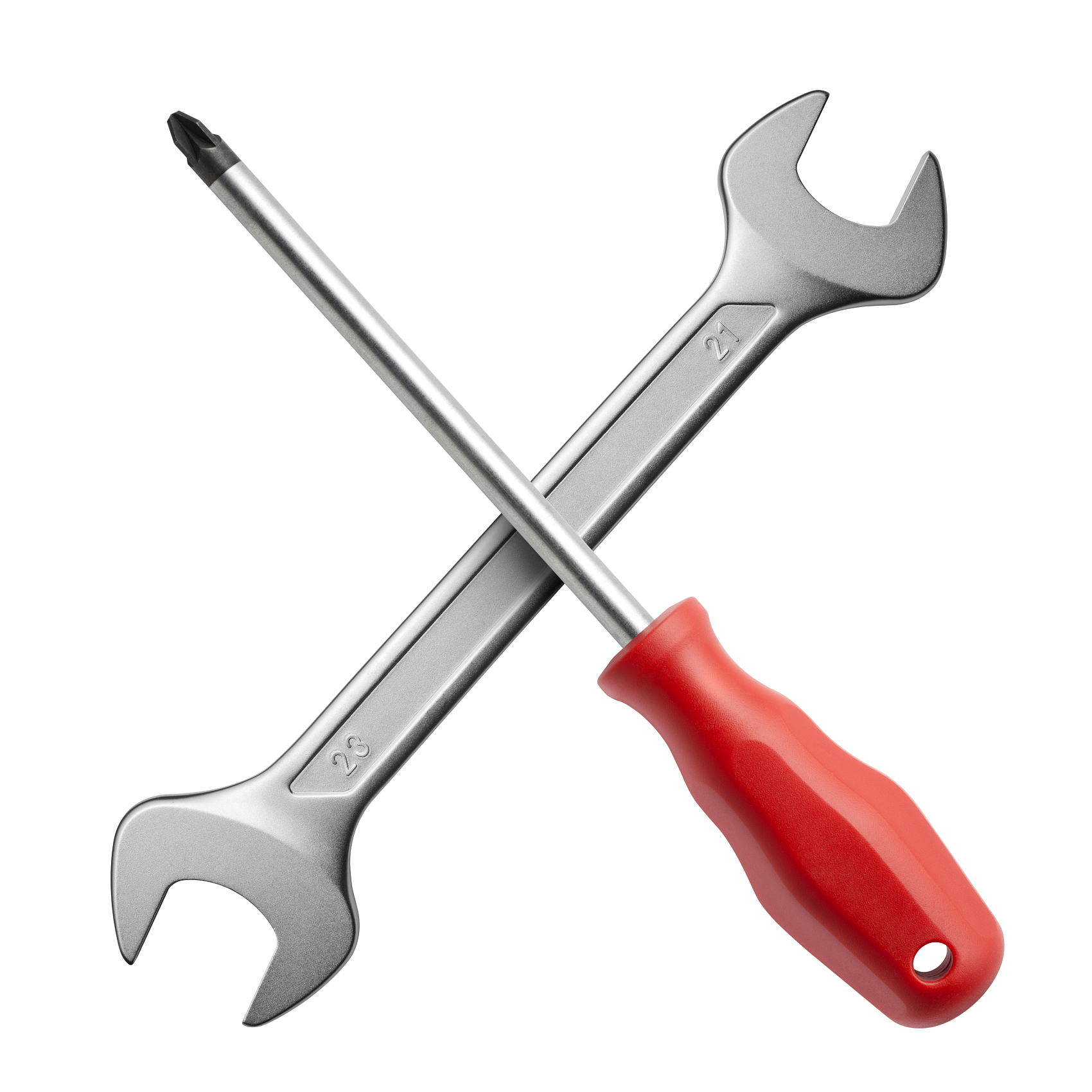


Two key and common defining elements of the guideline format, regardless of the model used, should be the transparency and explicitness of the process (i.e., sufficient detail so that the methodology could be reproduced and potential adopters are confident that the process used to adapt the guideline was rigorous and thorough) and the appropriate referencing and acknowledgement of intellectual credits to the source documents.

The Finalization Phase guides the user through the process of obtaining feedback on the document from stakeholders impacted by the guideline, consulting with the developers of source guidelines used in the adaptation process, establishing a process for the review and updating of the adapted guideline, and creating a final document.

| **Steps** | **Pruducts/**  **Deliverables** | **Skills and Organizational Requirements** | **Tools** |
| --- | --- | --- | --- |
| 19.External review by target users  20.Consult with relevant endorsement bodies  21.Consult with developers of  source guidelines  22. Acknowledge source documents | • Feedback from external review incorporated into guideline  • Approval by endorsing body(ies)  • Feedback from source guideline developers incorporated into guideline | Managerial and administrative skills | 18–Samples of External Review Survey |

**Step 19. External review - target audience of the guideline**

Once the panel has decided on the adaptation of their guideline, the next step is to send the adapted guideline to those who will be affected by its uptake (i.e., the users, including any practitioners who would use the guideline in practice like healthcare workers, medical researchers or any patient affected by the guideline).

Users also include, for example, policymakers, decision makers, organization representatives, and managers. Different questions might need to be asked of each group. The external review should ask questions about whether the users approve of the draft guideline, what its strengths and weaknesses are, and what requires modification. In addition, users might be asked questions around their confidence in the adaptation process, whether they would use the guideline in their practice, and how it would impactor change their current practice or routines. Users, administrators, and managers might be asked about the acceptability of the guideline for the organization and about the resource implications.

The external review can be performed by referring to or Chapter 11 of *the manual for the development of integrated Chinese and western medicine clinical guidelines*, edited by Lu Chuanjian et al (4), or by referring to the structured questionnaire of Tool 18, which is helpful for this step (17).

[**Tool 1**](#bookmark225)**8– Samples of External Review Surveys**


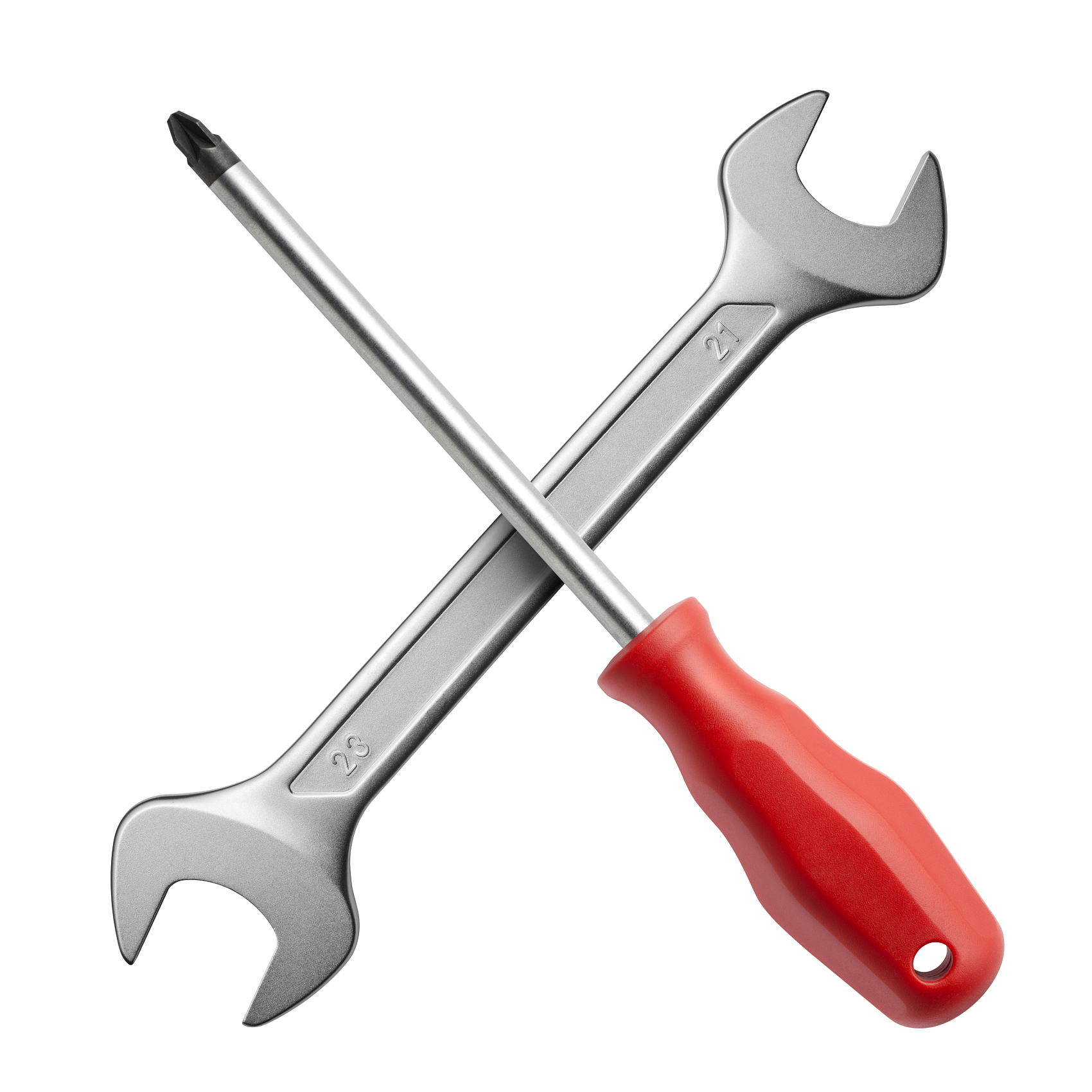


The purpose of this external review is to (1,2):

• Foster ownership and commitment of intended users toward the guideline

• Ensure that those most likely to use the guidelines will have the opportunity to review the guideline and provide feedback. This will help identify any areas not covered by the guideline, ensure that the recommendations are clear and applicable, and give an idea of the potential acceptance by the relevant uptake group.

• Allow managers and policy makers to consider the resources and other impacts of the guidelines and begin preparing for implementation

• Act as the first dissemination of the adapted guideline

The external review should ask questions about whether the reviewers approve of the draft guideline, what its strengths and weaknesses are, and what requires modification.

Electronic media can be used to collect any comments. All feedback received should be documented and discussed by panel and any changes made to the adapted guideline should be described. If the panel decides not to modify the guideline, regardless of the feedback received, this should also be documented, as well as the reasons for this decision.

**Step 20. Consult with endorsement bodies**

In order to help with widespread implementation, we recommend that the adapted guideline be formally endorsed or published by professional body(ies) or organization(s) most closely connected to the guideline topic (2)(e.g., Local medical administration or Chinese medicine (integrated Chinese and Western medicine) societies/associations or unions). The endorsement of a guideline by relevant professional organizations has been shown to enhance the acceptability of a guideline to the organization’s members (12). Endorsement can be a simple recognition by the organization of the relevance of the guideline to its members or a more formal process to implement the adapted guideline as policy within the organization. For example, a hospital endorsing a guideline to be implemented in one of its departments might commit resources to support the guideline, including any additional staff training that might be needed and soon. An organization with a nationally distributed membership might, among various dissemination options, provide the guideline as are source to its members or post it on its Website.

There is a set of specialized standard items and procedures for guideline development and issuance, which can be carried out according to the procedures of different standard-issuing departments in different regions.

**Step 21. Consult with source guideline developers**

The draft guideline maybe sent for feedback to any guideline developers whose recommendations have been used in the draft guideline, particularly in the case where changes have been made to the original recommendations.

**Step 22. Acknowledge source documents**

All documents used in the creation of the draft guideline should be referenced in the final document. The panel will need to determine whether they need to seek permission to use any guideline or guideline recommendation used in the adapted guideline. Requirements to seek permission should be available as part of the guideline document under a copyright clause. Information on sources, required permissions, and agreements should be kept in the project documentation.


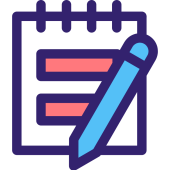


|  | **Illustration – Process of external review of the Chinese medicine diagnosis and treatment of psoriasis vulgaris guideline** |
| --- | --- |
| The draft guideline of Chinese medicine diagnosis and treatment of psoriasis vulgaris was sent to the local Chinese medicine societies and some grassroots community Chinese medicine practitioners for external review. In selecting the sample for review, the organizing committee attempted to select practitioners from across the county and working in both urban and rural practices. Practitioners were sent the draft guideline along with a short survey of questions about, for example, the practitioner’s confidence in the process, the applicability of the guideline to the practitioner’s patients and practice context, and whether the practitioner would use the guideline in practice. Practitioners were asked to provide feedback on the guideline itself, and in particular, the recommendations and the panel’s rationale for the recommendations. Feedback from practitioners was summarized and presented in a separate section of the guideline document labeled External Review. A response to the feedback by the panel was included.  The places where the feedback was used to alter the draft guideline were clearly indicated.The organizing committee decided to send a copy of the adapted guideline to the source developer for feedback (after completing their assessments, the panel decided that they would endorse one guideline without modification).  Prior to beginning the adaptation process, it was initially agreed to invite a local physician who is a standing member of the provincial TCM society's dermatology committee to participate in the process as a member of the expert panel, while an application for guideline publication was made at the local TCM society. Once the adapted guideline was finalized, it was submitted to the society for review and formal approval. The local TCM society published the guidelines, and the local TCM society's dermatology committee presented the guidelines at its annual meeting. | |

| **Steps** | **Pruducts/**  **Deliverables** | **Skills and Organizational Requirements** | **Tools** |
| --- | --- | --- | --- |
| 23. Plan for aftercare of the adapted  guideline Consult with relevant  endorsement bodies | • Plan for review and updates | Clinical expertise  Methodological expertise  Information retrieval skills | [Tool 19 –](#bookmark226) Report on the updating process |

**Step 23. Plan for aftercare of the adapted guideline**

Guideline updating requires a two-stage process, identifying new evidence and determining whether that new evidence warrants an update (11,12). New evidence might be identified through a focused literature review and/or through consultation with experts. Whether new evidence requires a guideline update depends on how extensively it impacts on the guideline’s recommendations (e.g., resource changes, outcome changes, technology changes, changes in existing benefits and harms, or changes in values related to outcomes). The extent of the update will depend on the results of the review, either to:

• discontinue use of the guideline;

• discontinue/withdraw some of the recommendations but not the entire guideline;

• redo the systematic review; or

• rewrite only those recommendations needing an update as long as the validity of the guideline is not compromised.

A review date should be decided upon, along with a process for dealing with reviewing the adapted guideline. Decisions about which review date to choose might be based upon when the [source guidelines f](#bookmark227)rom which recommendations were selected are updated or expire, or a choice of a set period (e.g., there is some evidence that guidelines might be outdated in as little as three to four years after their release (11). If the evidence in the adapted guideline has not been updated earlier on in the ADAPTE process (e.g., if the panel does not have the resources to do so), the challenge inherent in an adapted guideline made up of recommendations from a number of source guidelines is that each of the source guidelines may become outdated at different times.

The panel needs to decide who will undertake the initial search for new evidence at the scheduled review date. Depending on the extent of the update needed, the designated individual(s) will need to make decisions on what expertise and resources would be required and whether the process is feasible.

Depending on the extent of change, the updated guideline should be sent to a group of experts, stakeholders, and policymakers for external review. Feedback on the updated guideline should be incorporated in the final document.

[**Tool 19 –**](#bookmark228) **Report on Results of Updating Process**


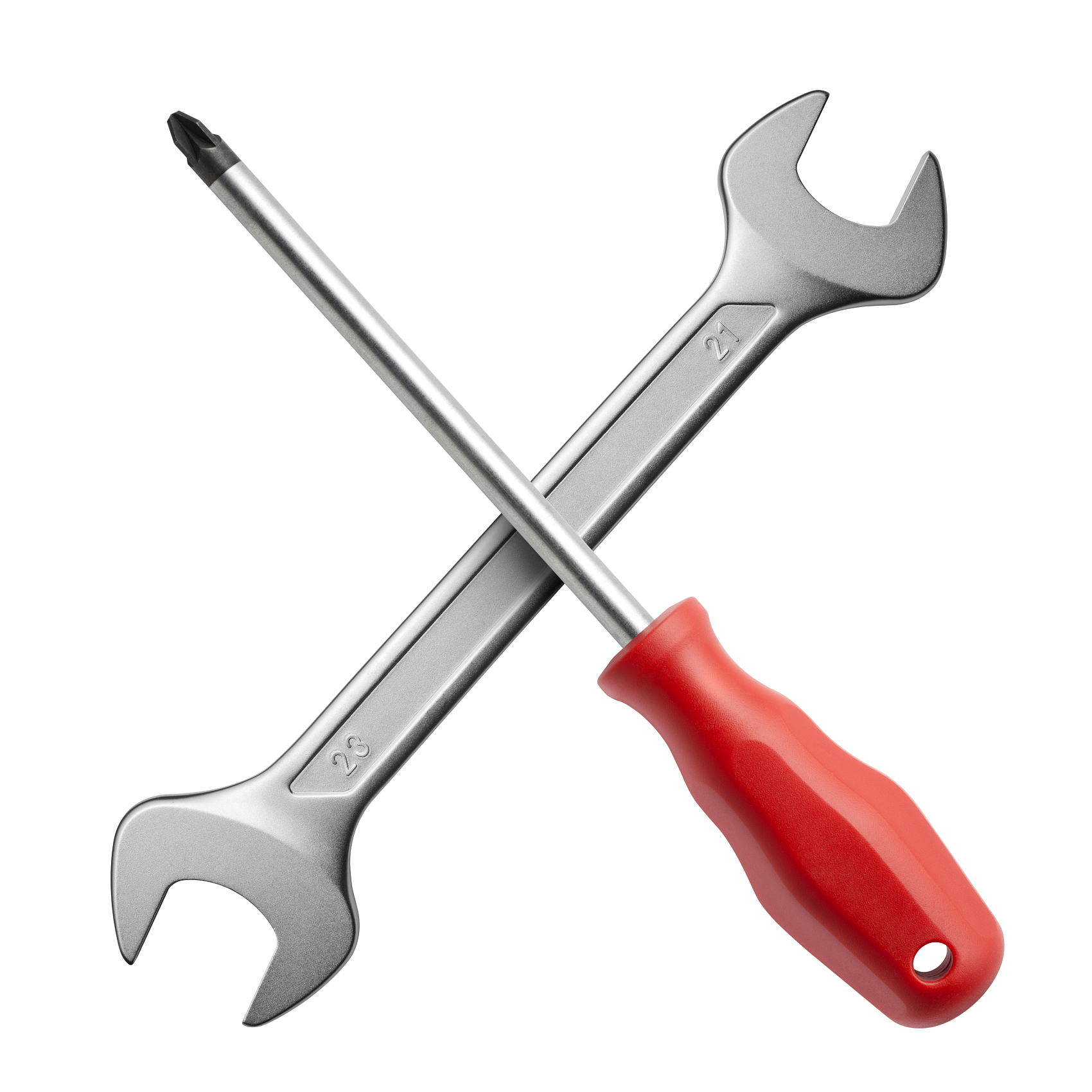


| 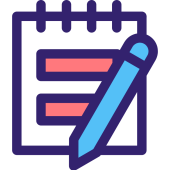**Illustration – Development of an updating plan** |
| --- |
| The chair of the organizing committee offered to take overall responsibility for deciding when a review and update of the adapted guideline might be necessary. He asked that those members of the panel with the relevant expertise assist with the actual work of update and review when the time comes.  As the adapted guideline is only based on one guideline, the panel decided that the chair  should keep in touch with the source guideline developers and monitor when they propose to review the evidence behind the source guideline and/or make substantive changes. The chair asked the resource team to monitor publication of new systematic reviews or health technology assessments reports particularly those related to changes in technology.  A plan for review was written up and put into the final adapted guideline. |

| **Steps** | **Pruducts/Deliverables** | **Skills and Organizational Requirements** | **Tools** |
| --- | --- | --- | --- |
| 24. Produce high quality | •Final guideline document  •Summary document and tools for application,e.g., patient information material | Editorial skills Design skills | [Tool 19 –](#bookmark226) Report on the updating process |

**Step 24. Produce final guidance document**

Implementation plans and customizing the adapted guideline are part of the adaptation process that occurs, or should occur, at the local level. At this level, the clinical implications and organizational and cultural context are fully understood, and the adapted guideline can be customized appropriately to take into account these considerations.

A final guideline product that is short, clear and unambiguous has been shown to make new guidelines more acceptable to physicians (aspects that are also applicable to adapted guidelines) (1,21). Algorithms or care pathways, checklists, and patient information material are desirable. How a document is formatted may modify the way a message is conveyed. The adapted guideline needs to be formatted for its intended group. While the implementation of research findings should be considered in producing the final document (e.g., recommending physician and patient reminder systems in those clinical areas where they have been shown to be effective), there are also a number of implementation resources available to assist in ensuring that the guideline is used in practice (see [Tool 1)](#bookmark229).

The final product might be reviewed using the AGREE II for TCM instrument (9) or RIGHT TCM extended version as a checklist to assess how the adapted guideline rates with respect to quality criteria.


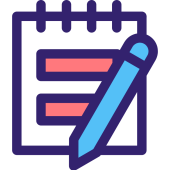


|  | **Illustration – Format of the final** | **guideline** | **document** |
| --- | --- | --- | --- |
| The final version of the adapted guideline was formatted to take into account the  preferences of family physicians. A one-page summary of the recommendations prefaced the main document. As it has been shown that reminders targeted at both the practitioner and the patient improve screening rates, the panel decided to produce a patient brochure that echoed the recommendations of the adapted guideline. The patient brochure was  translated into the languages of those populations in that locale that are traditionally underscreened,e.g., immigrant populations. | | | |

**Glossary**

**Acceptability**

Acceptability is defined as the extent to which the users are likely to adopt (see the term adoption below) a recommendation, based on internal qualities such as clarity,

comprehensiveness, and logical reasoning and on external factors such as the burden imposed on the process and system of care, patient and providers attitudes and beliefs, and patients needs, expectations, and preferences.

*Adapted from: Shiffman R, Dixon J, Brandt C, Essaihi A, Hsiao A, Michel G, et al. The GuideLine*

*Implementability Appraisal (GLIA): development of an instrument to identify obstacles to guideline development. BMC Med Inform Decis Mak. 2005;5:23.*

**Adaptation**

Adaptation of guidelines is the systematic approach to considering the use and/or

modification of (a) guideline(s) produced in one cultural and organizational setting for application in adifferent context. Adaptation can be used as an alternative to *de novo* guideline development or for customizing (an) existing guideline(s) to suit the local

context.

**Adoption**

Adoption of a guideline is the acceptance of a guideline as a whole after the assessment of

its quality, currency, and content. When healthcare providers (or other users of

recommendations) adopt a guideline, they feel committed to change their practices in accordance with the recommendations of the guideline.

*Adapted from: Davis DA and Taylor-Vaisey A. Translating guidelines into practice. A systematic*

*review of theoretic concepts, practical experience and research evidence in the adoption of clinical practice guidelines. Can Med AssocJ. 1997;157:408-16.*

**Applicability**

Applicability is defined as the extent to which the users are able to put a recommendation into practice, based on internal qualities such as a clearly defined eligible patient

population that matches the population to which the intervention is targeted in the local setting and external factors such as the availability of the necessary knowledge, skills,

provider time, staff, equipment, and other resources.

Applicability is sometimes taken as a synonym for feasibility:

• Feasibility of the acquisition of necessary skills and knowledge

• Feasibility of the necessary increase in provider time, staff, equipment, and soon.

Adapted from: Shiffman R, Dixon J, Brandt C, Essaihi A, Hsiao A, Michel G, et al. The GuideLine Implementability Appraisal (GLIA): development of an instrument to identify obstacles to guideline development. BMC Med Inform Decis Mak. 2005;5:23.

**Culture**

Culture represents the norms and values of a specific group, community, or population.

**Diffusion**

Diffusion is a passive means of transferring knowledge; it is not directed towards a target audience. An example of diffusion is the publication of articles in medical journals.

*Lomas J. Diffusion, dissemination and implementation: who should do what? In: Warren K,*

*Mosteller F editors., Annals of the New York Academy of Sciences: Doing more good than harm: the evaluation of healthcare interventions. Vol. 703. New York: New York Academy of Sciences; 1993.*

**Dissemination**

Dissemination is more active than diffusion in that it targets a specific audience and

involves tailoring the information for that audience. Examples of dissemination strategies include targeted mailings, presentations, and press conferences.

*Lomas J. Diffusion, dissemination and implementation: who should do what? In: Warren K,*

*Mosteller F editors., Annals of the New York Academy of Sciences: Doing more good than harm: the evaluation of healthcare interventions. Vol. 703. New York: New York Academy of Sciences; 1993.*

**Evidence-based principles**

Evidence-based medicine has been defined as "the conscientious, explicit, and judicious use of current best evidence in making decisions about the care of individual patients.

The practice of evidence based medicine means integrating individual clinical expertise with the best available external clinical evidence from systematic research."

*SackettDL, Rosenberg WM, Gray JA, Haynes RB, Richardson WS. "Evidence Based Medicine: What It Is and What It Isn't," BMJ 1996;312:71-2.*

**Evidence tables**

Evidence tables are summaries of the most salient information from studies identified in the systematic review. The elements of evidence tables are dependent on the types of

information in studies related to a particular topic but might include information such as the article reference, the study type (e.g., randomized controlled trial or cohort), the

number of patients and their characteristics, and the intervention, comparison arms, outcome measures, and effect sizes.

Scottish Intercollegiate Guidelines Network. SIGN 50: Aguideline developer’shandbook. 2001 [updated 2004 May]. Available from:[*www.sign.ac.uk/guidelines/fulltext/50/index.html*](http://www.sign.ac.uk/guidelines/fulltext/50/index.html)

**Guideline or Practice guideline**

“Systematically developed statements about specific health problems, intended to assist practitioners and patients in making decisions about appropriate healthcare.”

*Adapted from: Field MJ, LohrKN Editors; Committee on Clinical Practice Guidelines, Institute of Medicine. Guidelines for clinical practice:from development to use. Washington (DC):*

*National Academy Press; 1992.*

**Guideline consistency**

Agreement between the evidence and the recommendations, based on the:

• comprehensiveness of the study search and selection process,

• coherence between the results of the studies and their interpretation bythe guideline authors, and

• transparency between this interpretation and the recommendations.

**Guideline content**

In this document, guideline content refers to the recommendations in the source guidelines.

**Guideline currency**

A guideline maybe considered up to date “when [no] new information on interventions, outcomes, and performance justifies updating [it]. ”

*Shekelle P, Eccles MP, Grimshaw JM, Woolf SH. When should guidelines be updated? BMJ. 2001;323:155-7.*

**Guideline quality**

“By quality of clinical practice guidelines we mean the confidence that the potential biases of guideline development have been addressed adequately and that the

recommendations are both internally and externally valid, and are feasible for practice. This process involves taking into account the benefits, harms and costs of the

recommendations, as well as the practical issues attached to them. Therefore, the

assessment [of quality] includes judgements about the methods used for developing the guidelines, the content of the final recommendations, and the factors linked to their

uptake.”

*The AGREE Collaboration. Appraisal of Guidelines for Research and Evaluation (AGREE) Instrument.2001 Sep. Available from:*[*www.agreetrust.org*](http://www.agreetrust.org/)

**Guideline topic**

In this document, the topic refers to the theme of the guideline, as described in the

guideline title, for a targeted population (disease and patients) and intervention. The purpose, the audience, and the setting intended for the guideline, although not

necessarily explicitly stated in the title, are also part of the topic. A guideline on a given topic may contain more than one[health question.](#bookmark152)

**Health question**

The health question is a precisely described health issue (e.g., clinical, professional

practice or public health) relating to the topic of the guideline. A recommendation (and supporting evidence) is developed for each question. A guideline may include one or

more questions.

**Implementation**

“Implementation includes methods to promote the uptake of research findings into

routine healthcare in both clinical and policy contexts and hence to improve the quality

and effectiveness of healthcare. It includes the study of influences on healthcare professional and organisational behaviour.”

*Adapted from : Implementation Science.*[*www.implementationscience.com/info/about/.*](http://www.implementationscience.com/info/about/)

**Intraclass correlations**

Intraclass correlations provide a measurement of the extent to which two or more raters agree when rating the same set of things. The intraclass correlation is a reliability index and is typically a ratio of the variance of interest over the sum of the variance of interest plus error.

*Shrout P, FleissJ. Intraclass correlations: uses in assessingrater reliability. Psychol Bull. 1979;86(2):420-8.*

**Recommendation**

“Any statement that promote or advocate a particular course of action inclinical care.” Burgers JS. Quality of clinical practice guidelines [thesis]. Nijmegen: UMC St. Radboud; 2002.

**Stakeholder**

“A stakeholder is an individual, group and/or organization with a vested interest in your decision to implement a guideline. Stakeholders include individuals or groups who will be directly or indirectly affected by the implementation of a guideline.”

*Registered Nurses Association of Ontario). Toolkit: implementation of clinical practice guidelines. Toronto, Canada: Registered Nurses Association of Ontario; 2002.*

**Source Guideline**

In this document, source guidelines refer to those guidelines selected to undergo

assessments of quality, currency, content, consistency, and acceptability/applicability and upon which an adapted guideline maybe based.

**References**

1. Graham ID, Harrison MB, Brouwers M, Davies BL, Dunn S. Facilitating the use of evidence in practice: evaluating and adapting clinical practice guidelines for local use by health care organizations. J Obstet Gynecol Neonatal Nurs. 2002;31:599-611.

2. Graham ID, Harrison MB, Brouwers M. Evaluating and adapting practice guidelines for local use: a conceptual framework. In: Pickering S, Thompson J, editors. Clinical governance in practice. London: Harcourt, 2003: 213-229.

3. Guangdong Provincial Standardization Technical Committee of Traditional Chinese Medicine. DB44/T 2218.3-2019 General rules for the preparation and revision of clinical practice guidelines in traditional Chinese medicine (combined traditional Chinese and western medicine) Guideline project team construction and management [S]. Guangzhou, 2019.

4. Lu Chuanjian, Yang Kehu. Manual for the development of integrated Chinese and Western medicine diagnosis and treatment guidelines. Beijing: People's Health Publishing House, 2016.

5. Fink A, Kosecoff J, Chassin M, Brook R. Consensus methods: characteristics and guidelines for use. Am J Public Health. 1984;74:979-83.

6. Graham ID, Harrison MB. EBN users' guide: evaluation and adaptation of clinical practice guidelines. Evid Based Nurs. 2005;8:68-72.

7. Fervers B, Burgers JS, Haugh MC, Latreille J, Mlika-Cabanne N, Paquet L, et al. Adaptation of clinical guidelines: literature review and proposition for a framework and procedure. Int J Qual Health Care. 2006;18:167-76.

8. AGREE Collaboration. Appraisal of guidelines for research & evaluation (AGREE) instrument [monograph on the Internet]. 2001 Sep. Available from: http://www.agreetrust.org/docs/AGREE_Instrument_English.pdf

9.Xie X, Wang Y, Li H. AGREE II for TCM: Tailored to evaluate methodological quality of TCM clinical practice guidelines. Front Pharmacol. 2023 Jan 12;13:1057920. doi: 10.3389/fphar.2022.1057920.

10. Shrout P, Fleiss J. Intraclass correlations: uses in assessing rater reliability. Psychol Bull. 1979;86:420-8.

11. Shekelle PG, Ortiz E, Rhodes S, Morton SC, Eccles MP, Grimshaw JM, et al. Validity of the Agency for Healthcare Research and Quality clinical practice guidelines: how quickly do guidelines become outdated? JAMA. 2001;286:1461-7.

12. Shekelle P, Eccles MP, Grimshaw JM, Woolf SH. When should clinical guidelines be updated? BMJ. 2001;323:155-7.

13. Browman G, Zitzelsberger L, Boscaino A. Harnessing evidence to optimize cancer control: a pan-Canadian approach. Oncol Exch. 2005;4:22-5.

14.AGREE-REX Research Team (2019). The Appraisal of Guidelines Research & Evaluation—Recommendation Excellence (AGREE-REX) [EB/OL]. Retrieved July 23, 2024, from <https://www.agreetrust.org/wp-content/uploads/2019/04/AGREE-REX-2019.pdf>.

15. Xie R, Xia Y, Chen Y, Li H, Shang H, Kuang X, Xia L, Guo Y. The RIGHT Extension Statement for Traditional Chinese Medicine: Development, Recommendations, and Explanation. Pharmacol Res. 2020 Oct;160:105178. doi: 10.1016/j.phrs.2020.105178.

16. Song Y, Alonso-Coello P, Ballesteros M, et al. A Reporting Tool for Adapted Guidelines in Health Care: The RIGHT-Ad@pt Checklist[J]. Annals of Internal Medicine, 2022, 175(5):710-719. https://doi.org/10.7326/M21-4352

17. Brouwers M, Graham ID, Hanna SE, Cameron DA, Browman GP. Clinicians’ assessments of practice guidelines in oncology: the CAPGO survey. Int J Technol Assess Health Care. 2004;20:421-46

18. Grol R, Dalhuijsen J, Thomas S, Veld C, Rutten G, Mokkink H. Attributes of clinical guidelines that influence use of guidelines in general practice: observational study. BMJ. 1998;317:858-61.

**Tool 1: Guideline Development and Implementation Resources**

**URL Resources/References**

**Organization Name**

| National Health and  Medical Research Council (Australia) | http://www.nhmrc.gov.au | Clinical Practice Guideline Development Series Manual - 6 Toolkits |
| --- | --- | --- |
| Scottish Intercollegiate Guidelines Network | http://www.sign.ac.uk | SIGN guideline development manual: SIGN 50 |
| National Institute for Health and Clinical Excellence (UK) | http://www.nice.org.uk | “Using Guidelines” - Implementation section How we work - Developing NICE Clinical Guidelines |
| French National Authority for Health (HAS) | http://has-sante.fr | Clinical practice recommendations - Methodological basis for implementation in France Effectiveness of methods for implementing medical recommendations |
| Grading of Recommendations, Assessment, Development and Evaluation (GRADE) | http://www.gradeworkinggroup.org/ | See GRADE website |
| New Zealand Guideline Group | http://www.nzgg.org.nz | Evidence resources section has resources on developing guidance, evaluating guidance and tools |
| Joanna Briggs Institute (JBI) | http://joannabriggs.edu.au/pubs/ | FAME system for assigning levels of evidence to findings in JBI systematic reviews |
| Registered Nurses Association of Ontario | http://www.rnao.org | 2002Registered Nurses Association of Ontario. Toolkit: implementation of clinical practice guidelines.  Toronto, Canada: Registered Nurses Association of Ontario; 2002. |
| NHS Centre for Reviews and  Dissemination (UK) | http://www.york.ac.uk/inst/crd/ | NHS Centre for Reviews and Dissemination. Getting evidence into practice. Eff Health Care 1999；5 (1):1-16. |
| DSI Institut for Sundhedsvaesen (Denmark) | http://www.dsi.dk | Thorsen T, Makela M. editors Changing professional practice: theory and practice of clinical guidelines implementation. DSI rapport 99.05. Copenhagen, Denmark: Danish Institute for Health Services Research and Development； 1999. |
| Veterans Health Administration (USA) | http://WWW1.va.gov/health/ | Veterans Health Administration. Putting clinical practice guidelines to work in the Department of Veterans Affairs: A guide for action. |
| Yale University School of Medicine (USA) | http://www.biomedcentral.COM/147 2-6947/5/23 | Shiffman R, Dixon J, Brandt C, Essaihi A, Hsiao A, Michel G, et al. The GuideLine Implementability Appraisal (GLIA): development of an instrument to identify obstacles to guideline development. BMC  Med Inform Decis Mak. 2005；5:23. |
| World Health Organization | https://www.who.int/zh | WHO Guidelines Development Manual |
| World Federation of Chinese Medicine / Guangdong Provincial Administration of Market Supervision | http://183.62.15.51:8010/ | Handbook for the development of integrated traditional Chinese and Western medicine diagnosis and treatment guidelines |
| China Association of Chinese Medicine | https://www.ttbz.org.cn/StandardManage/Detail/57526/ | T/CACM 1336-2020 Development and Application Specification of Clinical Diagnostic Criteria for Traditional Chinese Medicine Based on Disease Syndrome Combination |
| China Association of Chinese Medicine | https://www.ttbz.org.cn/StandardManage/Detail/37042/  https://www.ttbz.org.cn/StandardManage/Detail/37045/  https://www.ttbz.org.cn/StandardManage/Detail/37046/ | T/CACM 1335.1-3-2020 Standardized Operating Procedures for Integrated Evidence Study of Traditional Chinese Medicine |
| China Association of Chinese Medicine | https://www.ttbz.org.cn/StandardManage/Detail/34381/ | Expert Consensus Technical Specifications in the Revision of T/CACM 1049-2017 Clinical Practice Guidelines for Traditional Chinese Medicine |
| China Association of Chinese Medicine | https://www.ttbz.org.cn/StandardManage/Detail/29053/ | T/CACM 1032-2017 Technical Process and Standards for the Development of Evidence Based Clinical Practice Guidelines for Traditional Chinese Medicine |

**Tool 2: Search Sources and Strategies**

**Sources for existing guidelines**

Guideline sources include both print publications and Web sites such as those for guideline clearinghouses and known developers as well as electronic databases, the reference lists in retrieved guidelines (hand searches), and panel members’ recommendations.

The table below only lists guidelines, standards websites and systematic reviews, and health technology assessment websites (the list is not exhaustive); journal literature databases are more widely used and are not listed. Retrieved references can be saved directly into reference software. The search strategy used (e.g., list of sources and terms) and the original location and/or source of the guide should be documented.

Retrieved references can be saved directly into reference software. The search strategy used (e.g., list of sources and terms) and the original locations and/or sources of the guidelines should all

be documented.

1. **Guideline clearinghouses and sources for systematic reviews and health technology assessments** (list is not exhaustive)

| **Guideline Internet Sites** | **URL** |
| --- | --- |
| National Guidelines Clearinghouse (NGC) | <http://www.guideline.gov/> |
| Guidelines International Network (GIN) | <http://www.g-i-n.net/> |
| Ontario Guidelines Advisory Committee (GAC) Recommended Clinical Practice Guidelines | [http://www.gacguidelines.ca](http://www.gacguidelines.ca/) |
| Institute for Clinical Systems Improvement (ICSI) | <http://www.icsi.org/knowledge/> |
| National Institute for Clinical Evidence (NICE) | <http://www.nice.org.uk/page.aspx?o=ourguidance> |
| New Zealand Guidelines Group | [http://www.nzgg.org.nz](http://www.nzgg.org.nz/) |
| Scottish Intercollegiate Guidelines Network (SIGN) | <http://www.sign.ac.uk/guidelines/index.html> |
| Canadian Agency for Drugs and Technology in Health | <http://www.cadth.ca/> |
| Canadian Medical Association Infobase | <http://mdm.ca/cpgsnew/cpgs/index.asp> |
| The Cochrane library | <http://www3.interscience.wiley.com/cgi-> bin/mrwhome/106568753/HOME |
| Food and Drug Administration | <http://www.fda.gov/cder/guidance/index.htm> |
| Centre for Reviews and Dissemination Health Technology Assessment Database | <http://www.york.ac.uk/inst/crd/crddatabases.htm#HTA> |
| Directory of evidence-based information Web sites | <http://132.203.128.28/medecine/repertoire/repertoire.asp> |
| Haute Autorité de Santé (HAS) | [http://has-](http://has-/)sante.fr/anaes/anaesparametrage.nsf/Page?ReadForm& Section=/anaes/SiteWeb.nsf/wRubriquesID/APEH-  3YTFUH?OpenDocument&Defaut=y& |
| CHU de Rouen - Catalogue & Index des Sites Médicaux Francophones (CISMef) | [http://doccismef.chu-](http://doccismef.chu-/)  rouen.fr/servlets/Simple?Mot=recommandations+professi onnelles&aff=4&tri=50&datt=1&debut=0&rechercher.x=2 9&rechercher.y=18 |
| Bibliothèque médicale AF Lemanissier | <http://www.bmlweb.org/consensus.html> |
| Direction de la lutte contre le cancer -  Ministère de la santé et des services sociaux du Québec | <http://www.msss.gouv.qc.ca/sujets/prob_sante/cancer/ind> ex.php?id=76,105,0,0,1,0 |
| SOR :Standards, Options et Recommandations | <http://www.fnclcc.fr/-sci/sor/index.htm> |
| Registered Nurses Association of Ontario | [http://www.rnao.org](http://www.rnao.org/) |
| Agency for Quality in Medicine | [http://www.aezq.de](http://www.aezq.de/) |
| Finnish Medical Society Duodecim | [http://www.kaypahoito.fi](http://www.kaypahoito.fi/) |
| American Society of Clinical Oncology | [http://www.asco.org](http://www.asco.org/) |
| Cancer Care Ontario Practice Guideline Initiative | [http://cancercare.on.ca](http://cancercare.on.ca/) |
| National Cancer Institute | [http://www.cancer.gov](http://www.cancer.gov/) |
| National Comprehensive Cancer Network | [http://www.nccn.org](http://www.nccn.org/) |
| Agence Française de Securite Sanitaire des Produits de Sante (AFSSAPS) | [http://afssaps.sante.fr](http://afssaps.sante.fr/) |

**2.Source of TCM / integrated Chinese and Western medicine guidelines (list is not detailed)**

| **Guideline Internet Sites** | **URL** |
| --- | --- |
| National group standard information platform | https://www.ttbz.org.cn/Home/Standard |
| China Association of Traditional Chinese Medicine | https://www.cacm.org.cn/category/zyzn/bzh/bzhsj/ Or TCM standardization public number |
| The World Federation of Traditional Chinese Medicine Societies | http://wfcms.org/list/52.html |
| The Chinese Society of Integrated Traditional Chinese and Western Medicine | http://www.caim.org.cn |
| China Association of Acupuncture and Moxibustion | http://www.caam.cn |
| The World Federation of Acupuncture Societies | http://www.wfas.org.cn |
| China Ethnic Chinese Medicine Association | http://www.chinaema.org.cn |
| The Chinese Information Society of Traditional Chinese Medicine | https://www.ciatcm.org.cn/ |
| The Chinese Medicinal Food Research Association | https://chinayaoshan.com.cn |
| China Association of Traditional Chinese Medicine | https://www.catcm.org.cn/index.html |
| Chinese Association of Minority Medicine | http://www.cmam.org.cn |
| CNKI (CNKI) | https://www.cnki.net |
| Wanfang full-text database | http://wanfangdata.svpcs.com.cn/ |
| VIP Chinese Technology Journal Database (VIP) | http://www.cqvip.com/ |
| Chinese Biomedical Literature Database (CBM) | http://www.sinomed.ac.cn/index.jsp |
| Medical pulse | https://www.medlive.cn/ |
| TCM standards and guidelines information service platform | http://standards.ccebtcm.org.cn/ |

**Choosing inclusion/exclusion criteria for guideline selection**

The chair or the panel will need to decide on some initial inclusion/exclusion criteria that will assist in the search and retrieval of guidelines. Some of the criteria that might be used include:

• Selecting only evidence-based guidelines (guideline must include a report on systematic literature searches and explicit links between individual recommendations and their supporting evidence)

• Selecting only national and/or international guidelines

• Specifying a range of dates for publication

• Selecting only those published since an important review was published

• Selecting peer reviewed publications only

• Selecting guidelines written in a particular language

• Excluding guidelines written by a single author not on behalf of an organization – in order to be valid and comprehensive, a guideline ideally requires multidisciplinary input

• Excluding guidelines published without references – as the panel needs to know

whether a thorough literature review was conducted and whether current evidence was used in the preparation of the recommendations

**Tool 3: Sample Declaration of Conflict of Interest**

**CONFLICT OF INTEREST**

**DISCLOSURE DECLARATION**

**NAME**

**NAME OF PANEL**

**DATE**

The following questions are designed to allow participants in the guideline appraisal group to

disclose any real or apparent conflict(s) of interest with respect to their activities in guideline

development. Conflicts of interest include the appraisers’ participation in the development or

endorsement of any of the guidelines that are being reviewed for the purpose of this project.

They may also involve relationships with pharmaceutical companies or other corporations whose products or services are related to the guideline topics. Financial interests or relationships

requiring disclosure include but are not limited to honoraria, consultancies, employment, or stock ownership.

The intent of the disclosure declaration is to have the participants in guideline appraisal identify any potential conflict(s) in relation to any of the guidelines that are under consideration in order that appraisal group members can form their own judgments, while taking the conflict(s) of

interest of other group members into consideration.

**Please answer each of the following questions by circling either “NO” or “YES” . If**

**you answer "YES" to any question, please describe the nature of the interest and/or relationship, and identify the relevant commercial entity.**

**1. PARTICIPATION IN GUIDELINE DEVELOPMENT**

Have you been involved in the development on any of the guidelines under review (e.g., a member of the guideline development committee)?

NO YES

If YES, please identify the guideline and describe your involvement:

Title of the guideline:

**2. GUIDELINE ENDORSEMENT**

Have you directly participated in any processes to formally endorse any of the guidelines under review?

NO YES

If YES, please identify the guideline and describe your involvement:

Title of the guideline:

|  |
| --- |
|  |
|  |
|  |

**3. EMPLOYMENT**

Are you or have you been employed by a guideline developer or an entity having a commercial interest in any of the guidelines under consideration?

NO YES

If YES, please describe:

**4. CONSULTANCY**

Have you served as a consultant for any guideline developer or an entity having a commercial interest in any of the guidelines under consideration?

NO YES

If YES, please describe:

**5. OWNERSHIP INTERESTS – PART A**

Do you have any ownership interests (including stock options) in any entity, the stock of which is not publicly traded, which has a commercial interest in any of guidelines under consideration?

NO YES

If YES, please describe:

**6. OWNERSHIP INTERESTS – PART B**

Do you have any ownership interests (including stock options but excluding indirect

investments through mutual funds and the like) valued at $1500 or more in any entity that has a commercial interest in any of the guidelines under consideration?

NO YES

If YES, please describe:

**7. RESEARCH FUNDING**

Are you currently receiving or have you received research funding from any entity that has a commercial interest in any of the guidelines under consideration?

NO YES

If YES, please describe:

**8. HONORARIA**

Have you been paid honoraria or received gifts of value equal to or greater than $3500 per year or $7500 over a three-year period from a guideline developer or an entity having a

commercial interest in any of the guidelines under consideration or from the developers of any of the guidelines under consideration?

NO YES

If YES, please describe:

**9. OTHER POTENTIAL CONFLICT(S) OF INTEREST**

**SIGNATURE**

**DATE** (Please print)

**Tool 4: Consensus Process Resources**

**References**

PagliariC, Grimshaw J. Impact of group structure and process on multidisciplinary evidence- based guideline development: an observational study. J Eval Clin Pract. 2002;8(2):145-53.

Raine R, Sanderson C, Hutchings A, Carter S, Larkin K, Black N. An experimental study of determinants of group judgments in clinical guideline development. Lancet. 2004;364(9432):429-37.

Hutchings A, Raine R. A systematic review of factors affecting the judgments produced by formal consensus development methods in healthcare. J Health Serv Res Policy. 2006;11(3):172-9.

Chinese Society of Traditional Chinese Medicine.T/CACM 1049-2017 Technical specifications for expert consensus in the formulation and revision of clinical practice guidelines for traditional Chinese medicine[S]. Beijing: Chinese Society of Traditional Chinese Medicine, 2017 [2023-11-22]. https://www.ttbz.org.cn/StandardManage/Detail/34381/.

**Tool 5: Example of Work Plan**

|  | **Guieline Phases** | **Taskes** | **Assigned To** | **Corresponding**  **Modules** | **Timeline** |
| --- | --- | --- | --- | --- | --- |
| **Preliminary Phase** |  | •Decide on broad topic area  •Assess feasibility of adaptation  •Identify needed resources  •Establish  multidisciplinary panel  •Write protocol  •Identify endorsing body  •Discuss authorship and accountability  •Discuss  dissemination and implementation | • Organizing committee | Preparation Module | Month 1 |
| **Adaptation Phase** | Initial Meeting (or conference call) | •Decide on terms of reference/consensus  • process  •Establish guideline inclusion/exclusion criteria  •Help identify key search terms  • Help identify key  documents/ sources | • Organizing committe  • Organizing committe  • Resource team  • Resource team  • Resource team | Preparation Module |  |
|  |  | • Refine topic area | • Panel | Scope and Purpose Module |  |
|  |  | •Complete guideline search  •Narrow list of CPGs (if needed) | • Resource team  • Organizing  committee/ resource team | Search and Screen Module |  |

|  | **Guieline Phases** | **Taskes** | **Assigned To** | **Corresponding**  **Modules** | **Timeline** |
| --- | --- | --- | --- | --- | --- |
|  |  | •Complete AGREE appraisal  •Assess guideline currency  •Complete evaluations (literature search and evidence, consistency of evidence and  conclusions,  conclusions and  recommendations) for all  recommendations (optional)  • Prepare  recommendations matrix  • Assess acceptability | • Panel  • Resource team  • Panel member(s)  • Resource team plus 1 clinician to review  • Panel | Assessment Module |  |
|  | Second meeting (face-to-face) | • Review all data  • Decide on  recommendations for adapted guideline | • Panel | Decision and Selection Module |  |
| **Finalization Phase** |  | •Write 1st draft of CPG and/or report on  process | • Chair | Customization Module |  |
|  | Third meeting (or conference call) | •ApprovE1st draft by panel | • Panel |  |  |
|  |  | •Send for external review and  consultation  • Get formal  endorsement | • Resource team  • Chair and designated panel member from professional society | External Review Module |  |
|  | Fourth meeting (or conference call) | • Discuss feedback from review and consultation | • Panel | Developing modules |  |
|  |  | •Decide on update process | • Panel | Aftercare planning Module |  |
|  |  | •Create final adapted guideline | • Designated author | Final Production Module |  |

|  | **Guieline Phases** | **Taskes** | **Assigned To** | **Corresponding**  **Modules** | **Timeline** |
| --- | --- | --- | --- | --- | --- |
| **Implementation Phase** |  | • Consider implementation issues and develop implementation plan | •Panel or implementation group |  |  |

**Tool 6: PIPOH**

*(NOTE: This tool was developed specifically for use in the adaptation of guidelines for Chinese Medicine/Chinese and Western Medicine.)*

The PIPOH items are:

• **P**atient population (including disease characteristics)

• **I**ntervention (s) of interest

• **P**rofessionals/patients (audience for whom the guideline is prepared)

• **O**utcomes to betaken into consideration (purpose of the guideline)

• **H**ealthcare setting and context

and their parameters, are to be used as prompts in the framing of the topic and health questions to be included or excluded from the guideline project.

For example, guideline developers and/or adapters might decide that a guideline on the general

topic of “Guidelines for TCM diagnosis and treatment of lung cancer” is to be developed. They then have to describe the population that the guideline is to discuss,e.g., which cancer stages, age groups, clinical circumstances,genetic considerations, and so forth, are to be included or excluded.

The kind of **i**nterventions to include or exclude are also to be decided, considering the following: Are only herbal treatments to be considered, or are other traditional Chinese medicine treatments such as acupuncture, moxibustion and tuina to be considered at the same time? Are other issues, such as diagnosis and daily regimen, considered?

The scope of the guideline also includes other considerations that guideline developers/adapters might want to discuss, including the following: Who is the intended audience of the guideline, **p**rofessional specialties and/or patients? As well, the purpose of the guideline should be defined, asking the question: What **o**utcomes are expected from publishing the guideline? Ideally, outcomes should be defined in away that provides benchmarks against which the impact of the guideline can be evaluated. Finally, the **h**ealthcare setting(s) where the guideline is to be implemented or exert its effects are to be described.

Framing the scope of the guideline as precisely as possible and as early as possible in the process of guideline development or adaptation facilitates the management of the project. The PIPOH checklist has been devised for such a purpose in the field of oncology.

**The PIPOH checklist for oncology**

Each PIPOH item,unless self explanatory, is followed by a brief tutorial.

|  |  | Inclue | Exclue | Detail |  |
| --- | --- | --- | --- | --- | --- |
| **Population (disease and patients characteristics)** | Site | □ | □ |  | |
|  | The majority of guidelines in the cancer field deal with at least one site (breast, colon, lung, etc..).However, guidelines can be produced that concern, for example, supportive treatments, where no specific site needs to be defined. | | | | |
|  | Stage | □ | □ |  | |
|  | Cancer stages can be described using a systematic terminology like that of the  AJCC :Cancer Staging Resource toolkit. Sixth edition. American Joint Committee on Cancer, Greene F.L.et al. Eds., Springer – Verlag, New-York, 2002  Some stages could be specifically excluded. For example *in situ* breast cancer | | | | |
|  | Histology |  |  |  | |
|  | Reference: Fritz A, Percy C, Jack A, Shanmugaratnam K, Sobin L, Parkin DM, Whelan S, editors. International classification of diseases for oncology. 3rd ed.. Geneva, Switzerland: World Health Organization； 2000. | | | | |
|  | Gender | □male  □female | □male  □female |  | |
|  | Age | □ | □ |  | |
|  | Clinically relevant examples for oncology :  □0-19 □19-49 □50-75 □75+ □premenopausal □postmenopausal | | | | |
|  | Clinical  circumstances | □ | □ |  | |
|  |  | □ | □ |  | |
|  |  | □ | □ |  | |
|  | Relevant examples for oncology: □treatment naive □ refractory □ optimal debulking or not □special physiological status like pregnancy □risk-modifying therapies (e.g., HRT) □high cancer risk group □performance status □comorbidity □neutropenia □hypercalcemia □diagnosis basis (e.g., clinical examination or tests) □ previous cancer □complications  tumour □ immunosuppression | | | | |
|  | **Genetics** | □ | □ |  | |
|  | Special genotypes (BRCA1 & 2, amplified HER2/*neu*) or phenotypes | | | | |
|  | Psychosocial/cultural | □ | □ |  | |
|  | For recommendations concerning, for example:  targeted supportive interventions  screening in specific professional groups  populations with a higher risk of cancer (Kaposi)  or recommendations in which self-reported symptoms are necessary (e.g., language barriers or education) | | | | |
|  | | | | | |
| **Interventions**  **Interventions** | Prevention-promotion | □ | ☑ |  | |
|  | Interventions that aim at modifying risks factors, risk evaluation included.  Examples of prevention interventions: □ Individual preventive measures □Public health interventions(e.g., heath education or preventive health services) □Environmental  □interventions □ Worksite □interventions □Interventions aimed at the organisation of health services | | | | |
|  | Screening | □ | □ |  | |
|  | Cancer detection in the population, genetic screening, screening processes, massscreening, early diagnosis, etc. | | | | |
|  | Diagnosis | □ | □ |  |  |
|  | Examples:  First evaluation  Physical examination  Tests  Surgery for diagnosis | | | | |
|  | Prognosis | □ | □ |  |  |
|  | E.g., markers | | | | |
|  | Treatment(s) | □ | □ |  | |
|  |  | □ | □ |  | |
|  |  | □ | □ |  | |
|  |  | □ | □ |  | |
|  | Examples of treatment topics in oncology:  □Internal treatment method of traditional Chinese medicine  □ External treatment method of traditional Chinese medicine  □Syndrome differentiation and treatment of internal treatment of traditional Chinese medicine  □traditional Chinese patent medicines and simple preparations  □Single herb extract of traditional Chinese medicine □ External treatment of drugs (Non pharmacological external treatment methods (needle therapy, ear needle therapy, fire needle therapy, moxibustion therapy, blood puncture and cupping therapy, cupping therapy) | | | | |
|  | Follow up  | □ | □ |  | |
|  | Rehabilitation | □ | □ |  | |
|  | End of life care | □ | □ |  | |
| **Professionals/Patients:**  **targeted users** | Providers | □ | □ |  | |
|  | □Chinese medicine practitioner □Chinese and western medicine practitioner  □ Western medicine practitioner □Chinese medicine nursing staff | | | | |
|  | Stakeholders | □ | □ |  | |
|  | □Hospital □Public health departments  □Government □Other organisations | | | | |
|  | Patients | □ | □ |  | |
|  | Should the guideline explicitly take into account patient preferences, opinions, expectations, and needs (reflected in the composition of the guideline development team) | | | | |
|  | | | | | |
| **Outcome – purpose of the guidlines** | Patients outcomes | □ | □ |  | |
|  | □Tumour response □ Survival □Disease-free survival □Quality of life (e.g., pain control,  psychological well being, performance status) □Innocuity □Test precision and reliability  □Treatment compliance | | | | |
|  | System outcomes | □ | □ |  | |
|  | □Costs  □Decrease in practice variation □Decrease in care system use  Improvements in quality of care indicators (e.g., appropriateness, optimized use, access, efficiency, timeliness, safety, continuity, etc.) | | | | |
|  | Public health  outcomes | □ | □ |  | |
|  | □Morbidity  □Mortality  □Incidence  □Prevalence | | | | |
|  | | | | | |
| **Healthy care setting** | Organisation | □ | □ |  | |
|  |  | □ | □ |  | |
|  | □Community Hospital □Chinese Medicine Hospital □Chinese and Western Medicine Hospital □General Hospital | | | | |

| **Other comments** |
| --- |
|  |
|  |
|  |

**Tool 7: Table for Summarizing Guideline Characteristics**

| **Title** | **Publisher** | **Country, language** | **Publication date** | **End of**  **search date** | **Comments** |
| --- | --- | --- | --- | --- | --- |
| ……………….  ………………. |  |  |  |  |  |
| ……………….  ………………. |  |  |  |  |  |
| ……………….  ………………. |  |  |  |  |  |
| ……………….  ………………. |  |  |  |  |  |
| ……………….  ………………. |  |  |  |  |  |
| ……………….  ………………. |  |  |  |  |  |
| ……………….  ………………. |  |  |  |  |  |

**Tool 8: Table for Summarizing Guideline Content**

|  |  | **Actual content of guidelines (CPG)**  (indicate with 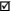 if included in guideline) | | | |
| --- | --- | --- | --- | --- | --- |
|  |  | **CPG #1** | **CPG #2** | **CPG #3** | **CPG #4** |
| **Health question #1** |  |  |  |  |  |
| **Health question #2** |  |  |  |  |  |
| **Health question #3** |  |  |  |  |  |
| **Health question #4** |  |  |  |  |  |
| **Health question #5** |  |  |  |  |  |
| **Health question #6** |  |  |  |  |  |
| **Population** | Insert definition here |  |  |  |  |
| **Intervention(s)** | Insert definition here |  |  |  |  |
| **Professionals/patients** | Insert definition here |  |  |  |  |
| **Outcome** | Insert definition here |  |  |  |  |
| **Healthcare setting** | Insert definition here |  |  |  |  |

**Population:** describe, if not adequately described in any health question discussed in the retrieved guidelines, the characteristics of the disease and patients for which there is to be some discussion (not necessarily a recommendation) in the guideline

**Intervention:** describe, if not adequately described in any health question discussed in the retrieved guidelines, the intervention(s) to be discussed

**Professionals/patients:** describe the targeted users of the guideline, e.g., specialists, professionals, and/or patients

**Outcome:** describe the purpose of the guideline and its objectives and outcome(s) against which an impact can be measured

**Healthcare setting:** describe the healthcare setting(s) in which the guideline is to be implemented

**Tool 9: AGREE II for Instrument**

**Evaluation system of Chinese Medicine Clinical Practice Guidelines and description of rules and regulations**

| **Item** | **Content description and scoring rules** |
| --- | --- |
| **Domain 1. Scope and purpose** | |
| 1 | **The overall objective(s) of the guidelines are clarified.**  **Description:**  This addresses the potential health effects of a set of guidelines on individuals, patient populations, and society as a whole. The overall objective(s) of the guidelines must be described in detail. For example, guideline contents include disease diagnosis, TCM syndrome diagnosis, treatment and prevention. The expected health benefits from the guidelines must be specific to the clinical problem or health topic. If it is a set of TCM guidelines for a certain disease, the purpose and advantages of TCM treatment must be clarified; the guidelines for using the disease name in Western medicine must specify the corresponding TCM disease name, and the guidelines for using the traditional Chinese medicine disease name must also specify the corresponding disease scope in Western medicine, For example, specific statements would be:   - These guidelines are applicable to patients with psoriasis vulgaris, which is known by names such as psoriasis vulgaris, tinea pinealis, dry tinea and snake lice. - These guidelines are applicable to cough, and common in Western medicine, such as in colds, acute bronchitis, chronic bronchitis, cough variant asthma and postnasal drip syndrome. - In treating this disease, TCM has a clear curative effect, can reduce skin lesions from erythema, scales and itching symptoms, reduce the disease recurrence, and delay the spread of the disease to other parts of the body.   **Rating:**  Item content includes the following criteria:   - health intent(s) (i.e., prevention, screening, diagnosis, treatment, prevention, etc.) - The disease names in both traditional Chinese medicine and Western medicine must be clarified in the guidelines (if appropriate). - advantages, expected benefits or outcomes of TCM treatment - target(s) (e.g., patient population, society)   Scoring:7 points will be awarded if all the content listed in “Evaluation” is explained in detail. 2-3 points will be deducted if any item is missing. Points will be deducted cumulatively according to how many items are missing, and only 1 point will be awarded if no content is described. |
| 2 | **The health question(s) covered by the guidelines are clarified.**  **Description:**  A detailed description of the health questions covered by the guidelines must be provided, particularly for the key recommendations (see Item 17). For example, specific statements would be:   - Can Chinese native medicine wash-outside in psoriasis treatment reduce the disease’s frequency? - Can biological agents combined with Chinese herbal decoction flakes improve the curative effect in plaque psoriasis treatment?   **Rating:**  Clinical problems cause PICO problems. Item content includes the following criteria:   - Target population (P) - intervention(s) or exposure(s) (e.g., Chinese herbal decoction flakes, Chinese patent medicine or Chinese medicine injection) (I) - comparisons (if appropriate) (C) - outcome(s) (Important patient outcome indicators should reflect the advantages of TCM treatment) (O) - healthcare setting or context   Scoring:7 points will be awarded if all the content listed in “Evaluation” is explained in detail. 2-3 points will be deducted if any item is missing. Points will be deducted cumulatively according to how many items are missing, and only 1 point will be awarded if no content is described. |
| 3 | **The population (patients, the general public, etc.) to whom the guidelines are meant to apply is described.**  **Description:** No change^a^  **Rating:**  The item’s content must include the following criteria: No change  Scoring:7 points will be awarded if all the content listed in "Evaluation" is explained in detail. 2-3 points will be deducted if any item is missing. Points will be deducted cumulatively according to how many items are missing, and only 1 point will be awarded if no content is described. |
| **Domain 2. Stakeholder involvement** | |
| 4 | **The guideline development group includes individuals from all relevant professional groups.**  **Description:**  This item refers to the professionals who were involved at some stage of the development process. This may include members of the steering committee, the research team involved in selecting and reviewing/rating the evidence, and individuals involved in formulating the final recommendations. This item excludes individuals who have externally reviewed the guidelines (see Item 13). It also excludes target population representation (see Item 5). Information about the guideline development group’s composition, discipline, and relevant expertise also must be provided.  **Rating:**  The item’s content must include the following criteria:  1) For each member of the guideline development group, the following information is included:   - name - discipline/content expertise (e.g., neurosurgeon, methodologist) - institution (e.g., St. Peter’s Hospital) - geographical location (e.g., Seattle, WA) - a description of the member’s role in the guideline development group   2) Are the members an appropriate match for the topic and scope? The members of the guideline development group must include experts of TCM or integrated traditional Chinese and Western medicine and experts of Chinese herbs. Other potential candidates include relevant clinicians and nurses, content experts, researchers, policy makers, clinical administrators.  3) Is there at least one methodology expert included in the development group (e.g., systematic review expert, epidemiologist, statistician, library scientist, etc.)?  Scoring:7 points will be awarded if all the content listed in "Evaluation" is explained in detail. 2-3 points will be deducted if any item is missing. Points will be deducted cumulatively according to how many items are missing, and only 1 point will be awarded if no content is described. |
| 5 | **The views and preferences of the target population (patients, the general public, etc.) have been sought.**  **Description:**  Information about the target population’s healthcare experiences and expectations should inform guideline development. There are various methods for ensuring that these perspectives inform the different stages of guideline development for stakeholders. Examples would include formal consultations with patients/the general public to determine priority topics, these stakeholders’ participation in the guideline development group, or external review by these stakeholders on draft documents. Alternatively, information could be obtained from interviews of these stakeholders, or from literature reviews of patient/general public’s values, preferences or experiences. There must be evidence that some process has taken place, and that stakeholders’ views have been considered.  **Rating:**  Item content must include the following criteria:   - The target population for collecting information must have experience with TCM diagnosis. - statement of strategy used to capture patients’/the general public’s views and preferences (e.g., participation in the guideline development group, literature review of values and preferences, or questionnaire survey) - outcomes/information gathered from patient/public information - description of how the information gathered informed the guideline development process, and/or drafting the recommendations   Scoring:7 points will be awarded if all the content listed in "Evaluation" is explained in detail. 2-3 points will be deducted if any item is missing. Points will be deducted cumulatively according to how many items are missing, and only 1 point will be awarded if no content is described. |
| 6 | **The guidelines’ target users are clearly defined.**  **Description:**  The target users must be clearly defined in the guidelines, so that the reader can immediately determine if the guidelines are relevant to them. For example, *TCM Guidelines for Diagnosis and Treatment of Common Diseases in Internal Medicine – Headaches* is applicable to the clinical diagnosis and treatment of headaches in TCM. It is applicable to all levels of TCM (Integrated Traditional Chinese and Western Medicine) medical institutions, as well as medical institutions offering TCM services. These guidelines’ target users may include Chinese medicine practitioners (Integrated Traditional Chinese and Western Medicine) and licensed assistant Chinese medicine practitioners (with the exception of Pediatrics). Clinical practitioners can also refer to them.  **Rating:**  The item’s content must include the following criteria:   - a clear description of the intended guideline audience (e.g. specialists, family physicians, patients, clinical or institutional leaders/administrators) - a description of how the guidelines may be used by their target audience (e.g., to inform clinical decisions, to inform policy, to inform standards of care)   Scoring:7 points will be awarded if all the content listed in "Evaluation" is explained in detail. 2-3 points will be deducted if any item is missing. Points will be deducted cumulatively according to how many items are missing, and only 1 point will be awarded if no content is described. |
| **Domain 3. Rigor of development** | |
| 7 | **Systematic methods were used to search for evidence.**  **Description**  Details of the strategy used to search for evidence must be provided; including the search terms used, sources consulted, and dates of the literature covered. Sources may include the tracing of ancient Chinese medical literature records and the searching for modern medical literature. Sources include ancient Chinese medical literature electronic databases (e.g., Chinese Medical Dictionary), Chinese electronic scholarly databases (e.g., CMKI, Wanfang, Wipu, CBM), English electronic scholarly databases (e.g., MEDLINE, ENBASE, CINAHL), other sources including systematic review databases (e.g., Cochrane Library, DARE), manually searched journals, conference proceedings, and other guidelines (e.g., the US National Guideline Clearinghouse, the German Guidelines Clearinghouse). The search terms must include the Chinese Medicine disease name and search terms such as "TCM, Chinese medicine, proprietary Chinese medicine, herbal medicine". The search strategy should be as comprehensive as possible and executed in a manner free from potential biases and detailed enough to be replicable.  **Rating**  The item’s content must include the following criteria:   - named electronic database(s) or evidence source(s) where the search was performed, ancient Chinese medical literature electronic databases (e.g., Chinese Medical Dictionary), Chinese electronic databases (e.g., CMKI, Wanfang, Wipu, CBM), English electronic databases (e.g., MEDLINE, ENBASE, CINAHL). - time periods searched (e.g., January 1, 2004 to March 31, 2008) - search terms used (e.g., text words, indexing terms, sub-headings) - full search strategy (e.g., possibly located in the appendix)   Scoring: 7 points will be awarded if all content listed in "Evaluation" is explained in detail. 2-3 points will be deducted if any item is missing. Points will be deducted cumulatively according to how many items are missing, and only 1 point will be awarded if no content is described. |
| 8 | **The criteria for selecting the evidence are clarified.**  **Description**  Criteria for the inclusion and exclusion of evidence at the time of search must be provided. These criteria and the reasons for exclusion/inclusion of evidence must be clarified. For example, the authors of a set of guidelines may decide to include only evidence from randomized controlled trials, and exclude non-Chinese literature.  **Rating**  The item’s content must include the following criteria:   - description of the inclusion criteria, including: - target population (patient, the general public, etc.) characteristics - study design - comparisons (if relevant) - outcomes - language (if relevant) - context (if relevant) - description of the exclusion criteria (if relevant; e.g., Chinese-language-only listed in the inclusion criteria statement could logically preclude non-Chinese-language from being listed in the exclusion criteria statement)   Scoring: 7 points will be awarded if all the content listed in "Evaluation" is explained in detail. 2-3 points will be deducted if any item is missing. Points will be deducted cumulatively according to how many items are missing, and only 1 point will be awarded if no content is described. |
| 9 | **The strengths and limitations of the body of evidence are clarified.**  **Description:** No change  **Rating:** No change  Scoring: 7 points will be awarded if all the content listed in "Evaluation" is explained in detail. 2-3 points will be deducted if any item is missing. Points will be deducted cumulatively according to how many items are missing, and only 1 point will be awarded if no content is described. |
| 10 | **The methods for formulating the recommendations are clarified.**  **Description**  A description of the methods used to formulate the recommendations and how final decisions were reached should be provided. For example, methods may include a voting system, informal consensus, and formal consensus techniques (e.g., Delphi, Glaser techniques). Experts participating in the drafting of recommendations must include experts of TCM, integrated traditional Chinese and Western medicine. Any areas of disagreement and the methods of resolving them should be specified. The TCM diagnosis must have clear sources and basis, such as expert consensus or literature.  **Rating**  The item’s content must include the following criteria:   - description of the recommendation development process (e.g., background information on the experts who participate in drafting the recommendations; the members of the expert group must include experts in TCM or integrative Chinese and Western medicine; steps used in the modified Delphi technique; voting procedures that were considered) - outcomes of the recommendation development process (e.g., the extent to which consensus was reached using the modified Delphi technique, outcome of voting procedures) - description of how the process influenced the recommendations (e.g., how the results of the Delphi technique influenced final recommendation, alignment with recommendations and the final vote)   Scoring: 7 points will be awarded if all the content listed in “Evaluation” is explained in detail. 2-3 points will be deducted if any item is missing. Points will be deducted cumulatively according to how many items are missing, and only 1 point will be awarded if no content is described. |
| 11 | **The health benefits, side effects, and risks have been considered in formulating the recommendations.**  **Description**  The guidelines should consider health benefits, side effects, and risks when formulating the recommendations.  For example, a set of guidelines on breast cancer management may include a discussion on the overall effects on various final outcomes. These may include: survival, quality of life, adverse effects, and symptom management, or a discussion comparing one treatment option to another. There should be evidence that these issues have been addressed. The characteristics and advantages of traditional Chinese medicine treatment must be explained; the health benefits and deficiencies of any Chinese medicinal herb with toxic side effects must be analyzed.  **Rating**  The item’s content must include the following criteria:   - supporting data and report of benefits - the characteristics and advantages of traditional Chinese medicine treatment - supporting data and report of harm/side effects/risks - reporting of the balance/trade-off between benefits and harm/side effects/risks, analysis of the health benefits and deficiencies of any Chinese medicinal herb with toxic side effects. - recommendations reflecting considerations of both benefits and harm/side effects/risks   Scoring:7 points will be awarded if all the content listed in "Evaluation" is explained in detail. 2-3 points will be deducted if any item is missing. Points will be deducted cumulatively according to how many items are missing, and only 1 point will be awarded if no content is described. |
| 12 | **There is an explicit link between the recommendations and the supporting evidence.**  **Description**  An explicit link between the recommendations and the evidence on which they are based should be included in the guidelines. The user of the guidelines should be able to identify the components of the body of evidence relevant to each recommendation. There must be clear correspondence between the etiology and pathogenesis, syndrome differentiation and treatment principles, therapeutic formulas or proprietary Chinese medicine in TCM guidelines. The composition of the therapeutic formulas in the recommendations must have the same name and composition as those in the evidence.  **Ratings**  The item’s content must include the following criteria:   - the guidelines describe how the guideline development group linked and used the evidence to inform recommendations (When evidence is lacking or a recommendation is informed primarily by consensus of opinion by the guideline group, rather than the evidence, is this clearly stated and described?) - each recommendation is linked to a key evidence description/paragraph and/or reference list - recommendations linked to evidence summaries, evidence tables in the results sections of guidelines; the etiology and pathogenesis, syndrome differentiation and classification in the TCM guidelines have clear correspondence with treatment principles, treatment prescriptions or proprietary Chinese medicines - the composition of the therapeutic formula in the recommendation must have the same name and composition as the formula in the supporting evidence.   Scoring: 7 points will be awarded if all content listed in "Evaluation" is explained in detail. 2-3 points will be deducted if any item is missing. Points will be deducted cumulatively according to how many items are missing, and only 1 point will be awarded if no content is described. |
| 13 | **The guidelines have been externally reviewed by experts prior to their publication.**  **Description**  Guidelines should be reviewed externally before they are published. Reviewers should not have been involved in the guideline development group. Reviewers should include experts in clinical TCM, as well as methodological experts. Target population representatives (e.g., patients, the general public) may also be included. A description of the methodology used to conduct the external review should be presented, which may include a list of the reviewers and their affiliations.  **Rating**  The item’s content must include the following criteria:   - a clear description of the intended guideline audience’s (e.g., specialists, family physicians, patients, clinical or institutional leaders/administrators) purpose and intent regarding the external review (e.g., to improve quality, gather feedback on draft recommendations, assess applicability and feasibility, disseminate evidence) - methods utilized for the external review (e.g., rating scale, open-ended questions) - description of the external reviewers (e.g., number, type of reviewers, affiliations) - outcomes/information gathered from the external review (e.g., summary of key findings) - description of how the information gathered was used to inform the guideline development process and/or drafting of recommendations (e.g., the guideline panel considered the review results in drafting the final recommendations)   Scoring: 7 points will be awarded if all the content listed in "Evaluation" is explained in detail. 2-3 points will be deducted if any item is missing. Points will be deducted cumulatively according to how many items are missing, and only 1 point will be awarded if no content is described. |
| 14 | **A procedure for updating the guidelines is provided.**  **Description:** No change  **Rating**  The item’s content must include the following criteria: No changes  Scoring: 7 points will be awarded if all the content listed in “Evaluation” is explained in detail. 2-3 points will be deducted if any item is missing. Points will be deducted cumulatively according to how many items are missing, and only 1 point will be awarded if no content is described. |
| **Domain 4. Clarity of presentation** | |
| 15 | **The recommendations are specific and unambiguous.**  **Description**  A recommendation must provide a concrete and precise description of which option is appropriate in which situation and in what population group, as informed by the body of evidence. The usage and dosage of the Chinese medicinal herb must be specified in the recommendation; the diagnostic criteria of TCM syndromes must be clear; there must be indications and detailed administration methods for TCM therapies; Chinese herbal medicines must have clear sources if they are non-self-formulated; there must be explanations for the special TCM terms that affect the guidelines.  It is important to note that in some instances, evidence is not always clear-cut and there may be uncertainty about the best care option(s). In this case, the uncertainty should be stated in the guidelines.  **Rating:**  The item’s content must include the following criteria:   - statement of the recommended action - identification of the intent or purpose of the recommended action (e.g., to improve quality of life, to mitigate side effects) - the content of the recommendations is clear and unambiguous - identification of the relevant population (e.g., patients, the general public) - caveats or qualifying statements, if relevant (e.g., patients or conditions for whom the recommendations would not apply)   Scoring: 7 points will be awarded if all the content listed in "Evaluation" is explained in detail. 2-3 points will be deducted if any item is missing. Points will be deducted cumulatively according to how many items are missing, and only 1 point will be awarded if no content is described. |
| 16 | **The options for management of the condition or health issue**  **are clearly presented**  **Description**  A set of guidelines that targets the management of a disease must consider all possible options for screening, prevention, diagnosis or treatment of the condition it covers. These possible options must be clearly presented in the guidelines.  For example, a recommendation on the management of depression may contain the following treatment alternatives:  a. Chinese medicine treatment  b. Acupuncture  c. Acupoint application  **Rating**  The item’s content must include the following criteria:   - a clear description of the intended guideline audience (e.g. specialists, family physicians, patients, clinical or institutional leaders/administrators), a description of options - description of the population or clinical situation most appropriate for each option   Scoring: 7 points will be awarded if all the content listed in "Evaluation" is explained in detail. 2-3 points will be deducted if any item is missing. Points will be deducted cumulatively according to how many items are missing, and only 1 point will be awarded if no content is described. |
| 17 | **Key recommendations are easily identifiable**  **Description:** No changes  **Rating**  The item’s content must include the following criteria: No changes  Scoring: 7 points will be awarded if all content listed in "Evaluation" is explained in detail. 2-3 points will be deducted if any item is missing. Points will be deducted cumulatively according to how many items are missing, and only 1 point will be awarded if no content is described. |
| **Domain 5. Applicability** | |
| 18 | **The guidelines describe facilitators and barriers to their application.**  **Description**  There may be existing facilitators and barriers that will influence the application of guideline recommendations. For example:  i. A set of guidelines on stroke may require that care be coordinated through stroke units and stroke services. There may be a special funding mechanism in the region to enable the creation of stroke units.  ii. A set of guidelines on diabetes in primary care may require that patients are seen and followed up in  diabetic clinics. There may be an insufficient number of clinicians available in a region to enable the establishment of clinics.  **Rating**  The item’s content must include the following criteria:   - identification of the facilitators and barriers that were considered - methods through which information regarding the facilitators and barriers to implementing recommendations were sought (e.g., feedback from key stakeholders, pilot testing of guidelines before widespread implementation) - information/description of the facilitators and barriers that emerged from the inquiry (e.g., certain therapeutic manipulation techniques in TCM guidelines, such as bone-setting techniques, require practitioners to have the appropriate skills to disseminate this recommendation) - description of how the information influenced the guideline development process and/or drafting of the recommendations   Scoring: 7 points will be awarded if all content listed in "Evaluation" is explained in detail. 2-3 points will be deducted if any item is missing. Points will be deducted cumulatively according to how many items are missing, and only 1 point will be awarded if no content is described. |
| 19 | **The guidelines provide advice and/or tools on how the recommendations can be put into practice.**  **Description**  For a set of guidelines to be effective, it needs to be disseminated and implemented with additional materials. For example, specific opinions for implementing the recommendations, such as conditions that need to be tailored to unique implementations, must be provided; Suggestions or practical booklets to guide the specific decoctions and administration methods for Chinese medicinal herbs. Any additional materials must be provided with the guidelines. These may include: a summary document, a quick reference guide, educational tools, results from a pilot test, patient leaflets, or computer support, such as mobile apps or websites.  **Ratings**  The item’s content must include the following criteria:   - an implementation section in the guidelines - tools and resources to facilitate application - guideline summary documents - links to checklists and algorithms - links to how-to manuals - solutions linked to barrier analysis (see Item 18) - tools to capitalize on guideline facilitators (see Item 18) - outcomes of pilot tests and lessons learned - directions on how users can access tools and resources   Scoring: 7 points will be awarded if all the content listed in "Evaluation" is explained in detail. 2-3 points will be deducted if any item is missing. Points will be deducted cumulatively according to how many items are missing, and only 1 point will be awarded if no content is described. |
| 20 | **The potential resource implications of applying the recommendations have been considered.**  **Description**  The recommendations may require additional resources in order to be applied. For example, there may be a need for more specialized staff, new equipment, or expensive drug treatment. These may have cost implications for health care budgets. The guidelines should contain a discussion regarding recommendations’ potential effects on resources. For example, the TCM guidelines for psoriasis may recommend that the dermatology department implement TCM external therapy or acupuncture for patients. To implement these recommendations, the departments must provide appropriate equipment, and the operators must have the appropriate qualifications.  **Rating**  The item’s content must include the following criteria:   - identification of the types of cost information under consideration (e.g., economic evaluations, drug acquisition costs) - methods by which the cost information was sought (e.g., a health economist was part of the guideline development panel, use of health technology assessments for specific drugs, etc.) - information/description of the cost information that emerged from the inquiry (e.g., specific drug acquisition costs per treatment course) - description of how the information gathered was used to inform the guideline development process and/or drafting of the recommendations - Were appropriate experts involved in finding and analyzing the cost information?   Scoring: 7 points will be awarded if all the content listed in "Evaluation" is explained in detail. 2-3 points will be deducted if any item is missing. Points will be deducted cumulatively according to how many items are missing, and only 1 point will be awarded if no content is described. |
| 21 | **The guidelines present monitoring and/or auditing criteria.**  **Description:** No changes  **Rating**  The item’s content must include the following criteria: No changes  Scoring: 7 points will be awarded if all the content listed in "Evaluation" is explained in detail. 2-3 points will be deducted if any item is missing. Points will be deducted cumulatively according to how many items are missing, and only 1 point will be awarded if no content is described. |
| **Domain 6. Editorial independence** | |
| 22 | **The views of the funding body have not influenced the content of the guidelines.**  **Description:** No changes  **Rating**  The item’s content must include the following criteria: No changes  Scoring: 7 points will be awarded if all content listed in "Evaluation" is explained in detail. 2-3 points will be deducted if any item is missing. Points will be deducted cumulatively according to how many items are missing, and only 1 point will be awarded if no content is described. |
| 23 | **Competing interests of guideline development group members have been recorded and addressed.**  **Description:** No changes  **Rating**  The item’s content must include the following criteria: No changes  Scoring: 7 points will be awarded if all the content listed in "Evaluation" is explained in detail. 2-3 points will be deducted if any item is missing. Points will be deducted cumulatively according to how many items are missing, and only 1 point will be awarded if no content is described. |

^a^No changes: the content did not change from AGREE Ⅱ.

**Tool 10: Quality evaluation of TCM diagnosis based on syndrome differentiation**

| Does the traditional Chinese medicine syndrome differentiation diagnosis in this guide come from systematic research on syndrome diagnosis criteria? | **Yes** | **No** |
| --- | --- | --- |
|  |  |  |
| Is the traditional Chinese medicine diagnosis based on literature research in this guideline? | **Yes** | **No** |
|  |  |  |
| Is the traditional Chinese medicine diagnosis based on expert consensus in this guideline? | **Yes** | **No** |
|  |  |  |
| Is the traditional Chinese medicine diagnosis based on epidemiological investigations in this guideline? | **Yes** | **No** |
|  |  |  |
| Is the TCM syndrome differentiation diagnosis in this guideline based on TCM syndrome diagnosis test research? | **Yes** | **No** |
|  |  |  |

**Tool 11: Sample Currency Survey of Guideline Developers**

| **Are you aware of any new evidence relevant to this clinical practice guidelines statement?** | **Yes** | **No** |
| --- | --- | --- |
|  |  |  |
| If so, please provide a reference for this new evidence. | | |
| **Is there any new evidence to invalidate any of the recommendations comprising the guideline?** | **Yes** | **No** |
|  |  |  |
| If so, please indicate which recommendation(s) are in need of updating, and provide the reference for this new evidence. | | |
| **Are there any plans to update the guideline in the near future?** | **Yes** | **No** |
|  |  |  |
| If so, when? | | |
| **When was the clinical practice guideline last updated?**  **What is the citation for the latest version?** | | |

**Tool 12: Sample Recommendation Matrix**

The following is an example of a recommendation matrix created for the creation of a guideline on systemic therapy for recurrent ovarian cancer using the adaptation process.

|  | **Dermatology Branch of Chinese Association of Traditional Chinese Medicine —— Psoriasis vulgaris (white) Clinical evidence-based practice Guide of Traditional Chinese medicine** | **WHO Western Pacific Region Grant —— TCM Clinical Practice guidelines for psoriasis vulgaris** | **World Federation of Chinese Medicine Societies —— Guidelines for integrated Chinese and Western medicine diagnosis and treatment of psoriasis vulgaris** |
| --- | --- | --- | --- |
| blood-heat syndrome | Treatment method —— to clear away heat, cool blood and detoxify | Treatment method —— to clear away heat, cool blood and detoxify | Treatment method —— to clear the heat and cool the blood |
|  | Recommended prescription medicine 1 cool blood detoxification soup 1: soil Poria cocos 30 g, Sophora japonica 15 g, purple grass 10 g, heavy building 9 g, raw rehmannia 15 g, white fresh skin 10 g, red peony root 10 g. One dose per day and two twice for 8 weeks.(Ib, strong recommendation)  Compound: 15g, 1,15 g, 30g, 12g, 12g, 9g, 12g, red peony 6g, 9g, 12 g, 30g, 9g, 6g schizonebera, 20g.1 dose of water every day, two oral, 100 ml, each course of 8 weeks, common adverse reactions are thin stool or increased stool frequency.(Ib, strong recommendation)  3: Sophora 30g, 30g, 15g, 15g, 15g, 15g, 15g, 15g, isatidis 30g, 30g, 15g, honeysuckle, forsythia 12g, 15 g, white skin 15 g.150ml twice daily for 2 months.(A, weak recommendation)  Recommended prescription 4 cool blood blood soup add and subtract: Sophora 30 g, white grass root 30g, rehmannia 30g, 15g, 15g, 15 g, Salvia miltiorrhiza 15g, chicken blood vine 30g, isatidis root 30g, 15 white skin 15 g. Dry stool is obvious to increase yellow, itching and even add skin, with sore pain plus forsythia, Scutellaria baicalensis, rash progress quickly with antelope horn powder rushed. One dose daily was oral twice after decocting in water for 2 months. The common adverse reaction was mild diarrhea.(A, weak recommendation)  Recommended prescription 5: composition: soil drink: honeysuckle 21 g, Poria 21 g, fried locust rice  15 g, 15 g rehmannia, 15 g peony bark, 15 g red baseony, 15 g purple grass, 15 g Salvia miltiorrhiza, 30 g root isatidis, 21 g white skin, 21g skin, and 6 g of licorice. One dose daily, fried in water, divided in two oral doses for 8 weeks.(A, weak recommendation)  Recommended prescription 6 silver soup: rehmannia, chicken blood vine, Sophora flower, purple grass, red peony, white root, Salvia miltiorrhiza, peony skin, white fresh skin. One dose in water a day in two oral doses for 2 months.(A, weak recommendation)  Recommended prescription 7 composition: 30 g, Poria cocos, 15 g, Sophora sinensis, 20g, 10g, and 5 g licorice. Add and subtraction: for swollen and sore throat, add blue root and bean root; constipation, add yellow; pruritus is obvious, add white skin and black snake; more scales, add white peony root and angelica, plus rash and zedoary. One dose daily, fried in water, divided in two oral doses for 8 weeks.(A, weak recommendation)  Recommended prescription 8 composition: 15 g, 15g, 30g, 30g, 30 g, 15g, 15g, 15g, 15g, 15g, 30 g, 15 g, 15 g, 15 g, 15 g, 0. 6 g of antelope horn powder (blunt). Add and minus: 6g, bean root, 15g, 15g, fresh skin, and 10 g; 6 g (after lower). One dose per day, fried in water, divided in two oral doses for 40 days.(A, weak recommendation) | Recommended prescription: keyin side: Poria, honeysuckle, north bean root, flea, white skin, white spirit fairy, isatidis root, raw licorice.(Recommended strength: B, evidence level: level b) | Treatment classic prescription: can use rhinoceros horn hmannia soup (source "Outer Taiwan secret" or Qing ying soup (source "warm disease"). Other therapeutic prescription: can use clear heat cool blood soup or cool blood detoxification soup. |
| Chinese patent drug | Compound Qingdai capsule (pill) oral administration, mainly suitable for blood heat syndrome.(A, weak recommendation) | Blood heat syndrome: silver elimination particles, 3.5g in each bag, 1 bag for each time, 3 times a day.(Recommended strength: B, evidence level: a); gram silver pills, 10g per bag, twice a day, 1 bag for each time.(Recommended intensity: B, Evidence level: b); Compound Qingdai Capsule, 0.5g each, 3 times a day, 3 capsules each time.(Recommended strength: B, Evidence level: A)  Recommended TCM injection: Qingkailing injection [08-69140~60ml, add 5% glucose solution or 0.9% normal saline intravenous drip, once a day.(Recommended strength: B, level of evidence: Ib) | Blood fever syndrome: it is recommended to use compound Qingdai capsule (pill), silver particles, silver particles, psoriasis capsule, psoriasis capsule, psoriasis capsule, can use keyin pill, silver capsule, silver capsule, silver capsule; |
| Traditional Chinese medicine bath method | It can be used for each syndrome type, especially the blood dryness syndrome and blood stasis syndrome are the most suitable, but if the blood heat syndrome is bright red or progressing quickly, the drug bath method should not be applied. The principle of TCM medicine should be to avoid allergies and drug irritation.  Recommended medicine bath prescription: Salvia miltiorrhiza, angelica, red peony root, earth skin, snake bed, white skin, bitter ginseng 30 g each, used for blood dryness syndrome.(A, weak recommendation) | Medicine bath no. 1 square: rhubarb, cypress, bitter, weed, wild chrysanthemum, snake bed, dandelion, angelica dahurica, calamus, safflower, mint, skin, alum. Put the first 11 Chinese medicines into the steam pot, add 180~200L, boil with steam for 20 minutes, filter the slag into the bathtub, and then add the skin and alum, melt to warm, do a whole medicine bath, 20 minutes each time, twice a day, for 4 weeks for 1 course of treatment, suitable for the blood heat of ordinary psoriasis.(Recommended strength: B, level of evidence: a)  Medicine bath no. 2 square: large land, angelica, angelica, chicken, blood vine, five skin, ground skin, flea, Xu Changqing, thorn, chrysanthemum, white fairy, peach leaves, cypress leaves, Salvia miltiorrhiza, Chinese prickly ash. Put all the drugs into the steam pot, add 180~200L, boil with steam for 20 minutes, pour the filter residue into the bathtub, until warm, do a whole body medicine bath, 20 minutes each time, 2 times a day, suitable for blood deficiency syndrome of ordinary psoriasis.(Recommended strength: B, level of evidence: a) | Traditional Chinese medicine bath method is recommended.  Indications: For skin lesions of common psoriasis in each stage and syndrome differentiation, clear heat and cool blood drugs, blood activating drugs, dispel wind, moisten dryness and stop itching drugs can be selected according to different syndrome types. Reduce the use of acute use, and peaceful drugs should be used when necessary.  Common medicine: Traditional Chinese medicine can be selected according to different syndrome types: a) clear heat cool blood medicine: Scutellaria baicalensis, dandelion, purple flower, Coptis chinensis, cypress, Scutellaria baicalensis, bitter ginseng, purple grass, Xu Changqing, white skin, white skin, lateral cypress, rehmannia, etc.; b) blood circulation medicine: Salvia miltiorrhiza, red peony, etc.; c) dispel wind and stop itching medicine: snake bed, etc.  Usage and dosage: The dosage of each Chinese medicine should be 15~30g, which is composed by the doctor under the guidance of syndrome differentiation and treatment. One dose a day, decocting 3 times, each time for 40 minutes, mix the fried liquid, and then add the right amount of hot water. the water volume of the whole body bath is about 50-60L (the water volume is adjusted according to the size of the bath tool, and the semi-sitting position or sitting liquid is soaked but the chest), and the water volume of foot bath and local immersion should be reduced as appropriate. The water temperature should be 35-38℃, and the time of bathing, foot bathing and local soaking should be 15-20 minutes. |

**Tool 13: Evaluation Sheet – Search and Selection of Evidence**

|  | **Guideline #1** | | | **Guideline #2** | | |
| --- | --- | --- | --- | --- | --- | --- |
|  | **Yes** | **Unsure** | **No** | **Yes** | **Unsure** | **No** |
| **Overall, was the search for evidence comprehensive?** | 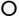 | 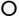 | 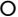 | 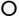 | 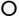 | 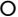 |
| The authors had a clearly focused question (population, intervention, outcome) | 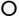 | 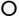 | 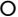 | 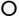 | 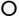 | 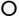 |
| Appropriate databases were searched for source guidelines | 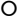 | 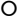 | 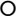 | 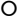 | 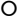 | 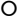 |
| Internet sites were searched for source guidelines | 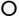 | 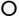 | 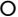 | 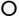 | 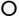 | 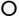 |
| Years covered in search | 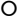 | 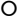 | 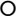 | 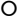 | 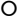 | 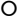 |
| Languages covered in search | 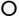 | 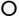 | 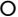 | 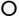 | 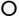 | 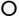 |
| Keywords used | 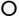 | 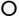 | 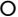 | 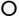 | 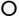 | 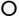 |
| Combinations of keywords | 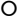 | 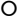 | 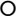 | 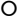 | 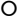 | 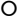 |
| Detailed search strategies are provided with the guideline | 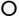 | 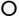 | 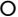 | 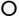 | 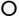 | 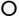 |
| Snowball methods were used | 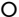 | 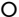 | 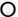 | 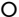 | 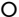 | 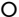 |
| A hand search of the reference lists was completed | 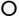 | 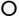 | 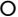 | 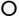 | 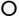 |  |
| Local experts and/or societies were asked for guideline recommendations |  |  |  |  |  |  |

|  | **Guideline #1** | **Guideline #2** |
| --- | --- | --- |
|  | **Yes** **Unsure No** | **Yes** **Unsure No** |
| **Overall, was bias in the selection of articles avoided?** |  |  |
| Inclusion and exclusion criteria reported |  |  |
| The number of persons who selected and analysed the data is documented |  |  |
| The procedure to solve disagreement is described |  |  |
| The number of references analysed is documented |  |  |
| The number of excluded references is documented |  |  |
| The reasons for excluding references are given |  |  |
| The criteria for inclusion and exclusion are clinically and methodologically valid |  |  |
| The reasons for exclusion conform to the selection and exclusion criteria |  |  |
| The process for selection of evidence is adequately described |  |  |
|  | **Comments** | **Comments** |

**Tool 14: Evaluation Sheet – Scientific Validity of Guidelines (Consistency between Evidence, Its Interpretation and Recommendations)**

| **Health question 1** | **Guideline #1** | | | **Guideline #2** | | |
| --- | --- | --- | --- | --- | --- | --- |
|  | **Yes** | **Unsure** | **No** | **Yes** | **Unsure** | **No** |
| **Overall, the evidence was valid** |  |  |  |  |  |  |
| Given the search strategy, the risk that relevant evidence has been missed is low |  |  |  |  |  |  |
| The criteria for selecting the evidence is explicit |  |  |  |  |  |  |
| Settings and protocols of selected studies fit with the health question |  |  |  |  |  |  |
| Outcomes were clinically sound (e.g., duration of disease-free survival might be considered too weak as evidence compared to overall survival) |  |  |  |  |  |  |
| The criteria used for assessing the quality and validity of the selected studies are adequately reported (type of studies, randomization methods, patient retention in groups etc.) |  |  |  |  |  |  |
| The risk that biased evidence has been reported is low |  |  |  |  |  |  |
| The outcomes were considered clinically sound (e.g., duration of disease free survival might be considered too weak as evidence compared to overall survival) |  |  |  |  |  |  |
| When a meta-analysis was performed, statistical analyses were appropriate. Sensitivity analysis and test of heterogeneity was performed |  |  |  |  |  |  |

| . **Health question 1** | **Guideline #1** | | | **Guideline #2** | | |
| --- | --- | --- | --- | --- | --- | --- |
|  | **Yes** | **Unsure** | **No** | **Yes** | **Unsure** | **No** |
| **Coherence between the evidence and** · **recommendations** |  |  |  |  |  |  |
| The evidence was direct. Patients and  interventions included in the studies were comparable to those targeted by the  recommendation |  |  |  |  |  |  |
| Conclusions were supported by data and/or the analysis; results were consistent from study to study. When inconsistencies existed in data,  considered judgment was applied and reported. |  |  |  |  |  |  |
| The conclusions are clinically relevant. (Statistical  significance is not always equal to clinical significance) |  |  |  |  |  |  |
| The conclusions derived from data point to  effectiveness/ineffectiveness of the intervention and the recommendation is written accordingly |  |  |  |  |  |  |
| There is some justification to recommend/not recommend the intervention eventhough the evidence is weak |  |  |  |  |  |  |
| The hierarchy of strength of evidence is adequately described |  |  |  |  |  |  |
| **Overall, the scientific quality of this recommendation does not present risks of bias** |  |  |  |  |  |  |
| The strength of evidence attributed to the  recommendation is adequately described and justified |  |  |  |  |  |  |
| Risks and benefits have been weighed |  |  |  |  |  |  |

(Process is repeated as needed for additional health questions)

**Tool 15: Evaluation sheet – Acceptability/Applicability**

| **Health question 1** | **Guideline #1** | | | **Guideline #2** | | |
| --- | --- | --- | --- | --- | --- | --- |
|  | **Yes** | **Unsure** | **No** | **Yes** | **Unsure** | **No** |
| **Overall, the recommendation is acceptable** |  |  |  |  |  |  |
| The strength of evidence and the magnitude of effect adequately support the grade of the  recommendation |  |  |  |  |  |  |
| There is sufficient benefit of the intervention, compared with other available management |  |  |  |  |  |  |
| The recommendation is compatible with the culture and values in the setting where it is to be used |  |  |  |  |  |  |
|  |  | **Comments** |  |  | **Comments** |  |
|  | **Yes** | **Unsure** | **No** | **Yes** | **Unsure** | **No** |
| **Overall, the recommendation is applicable** |  |  |  |  |  |  |
| The intervention is applicable to the patients in the context of use |  |  |  |  |  |  |
| The intervention/equipment is available in the context of use |  |  |  |  |  |  |
| The necessary expertise is available in the context of use |  |  |  |  |  |  |
| There are no constraints, legislation, policies, or resources in the healthcare setting of use that would impede the implementation of the recommendation |  |  |  |  |  |  |

(Process is repeated as needed for additional health questions)

**Tool 16: Checklist of Adapted Guideline Content**

| **Guideline section** | **When to be completed/ Completed** |
| --- | --- |
| 1. Overview material  • Structured abstract including:  o Guideline’srelease date  o Status (original, adapted, revised, updated)  o Print and electronic sources  • Adapter and source guideline developer |  |
| 2. Introduction and background |  |
| 3. Scope and purpose |  |
| 4. Target audience of the guideline |  |
| 5. Health questions |  |
| 6. Recommendations  • Risks and benefits associated with the recommendations  • Specific circumstances under which to perform the recommendation  • Strength of recommendation (if assigned) |  |
| 7. Supporting evidence and information for the recommendations  • Panel rationale behind the recommendations  • Presentation of additional evidence  • How and why existing recommendations were modified |  |
| 8. External review and consultation process  • Who was asked to review the guideline  • What process was followed  • Discussion of feedback  • Feedback incorporated into the final document |  |
| 9. Plan for scheduled review and update |  |
| 10. Algorithm or summary document |  |
| 11. Implementation considerations |  |
| 12. Glossary (for unfamiliar terms) |  |
| 13. References of all material used in creating the guideline |  |
| 1. Acknowledgment of source guideline developers and permission granted (where necessary) |  |
| 15. List of panel members and their credentials, declaration of conflicts of interest |  |
| 16. List of funding sources |  |
| 17. Appendix describing adaptation process including:  • Guideline search and retrieval including list of guidelines and whether they were included/excluded, with rationale  • Guideline assessments including a summary of results for each assessment (including AGREE domain scores)  • Decision process followed by panel  • Results and decisions of each evaluation |  |

**Tool 17: Checklist of the RIGHT Extension Statement for traditional Chinese medicine (RIGHT-TCM)**

| **Section/Topic** | **Number** | **Item** |
| --- | --- | --- |
| **Basic information** |  |  |
| Title/subtitle | 1a | Identify the report as a guideline, that is, with “guideline(s)” or “recommendation(s)” in the title. |
|  | S1 | Identify as a clinical guideline for traditional Chinese medicine through the title. |
|  | 1b | Describe the year of publication of the guideline. |
|  | 1c | Describe the focus of the guideline, such as screening, diagnosis, treatment, management, prevention, or others. |
| Executive summary | 2 | Provide a summary of the recommendations contained in the guideline. |
| Abbreviations and acronyms | 3 | Define new or key terms, and provide a list of abbreviations and acronyms if applicable. |
| Corresponding developer | 4 | Identify at least 1 corresponding developer or author who can be contacted about the guideline. |
| **Background** |  |  |
| Brief description of the health problem(s) | 5 | Describe the basic epidemiology of the problem, such as the prevalence/incidence, morbidity, mortality, and burden (including financial) resulting from the problem. |
| Aim(s) of the guideline and specific objectives | 6 | Describe the aim(s) of the guideline and specific objectives, such as improvements in health indicators (e.g., mortality and disease prevalence), quality of life, or cost savings. |
| Target population(s) | 7a | Describe the primary population(s) that is affected by the recommendation(s) in the guideline. |
|  | 7b | Describe any subgroups that are given special consideration in the guideline. |
| End users and settings | 8a | Describe the intended primary users of the guideline (such as primary care providers, clinical specialists, public health practitioners, program managers, and policymakers) and other potential users of the guideline. |
|  | 8b | Describe the setting(s) for which the guideline is intended, such as primary care, low- and middle-income countries, or inpatient facilities. |
| Guideline development groups | 9a | Describe how all contributors to the guideline development were selected and their roles and responsibilities (e.g., steering group, guideline panel, external reviewers, systematic review team, and methodologists). |
|  | 9b | List all individuals involved in developing the guideline, including their title, role(s), and institutional affiliation(s). |
| **Evidence** |  |  |
| Health care questions | 10a | State the key questions that were the basis for the recommendations in PICO (population, intervention, comparator, and outcome) or other format as appropriate. |
|  | 10b | Indicate how the outcomes were selected and sorted. |
| Systematic reviews | 11a | Indicate whether the guideline is based on new systematic reviews done specifically for this guideline or whether existing systematic reviews were used. |
|  | 11b | If the guideline developers used existing systematic reviews, reference these and describe how those reviews were identified and assessed (provide the search strategies and the selection criteria, and describe how the risk of bias was evaluated) and whether they were updated. |
| Assessment of the certainty of the body of evidence | 12 | Describe the approach used to assess the certainty of the body of evidence. |
| **Recommendations** |  |  |
| Recommendations | 13a | Provide clear, precise, and actionable recommendations. |
|  | S2 | Describe the principle and method of treatment for traditional Chinese medicine in the recommendations. |
|  | S3 | Describe whether to treat disease based on the syndrome differentiation of traditional Chinese medicine. |
|  | S4 | Provide clear and accurate description of traditional Chinese medicine decoction in the intervention. |
|  | S4-1 | Describe the administration route (e.g., oral, topical), frequency of traditional Chinese medicine decoction. |
|  | S5 | Provide clear and accurate description of the acupuncture in the intervention. |
|  | S5-1 | Describe the acupuncture points, major points, matching points, and their addition and subtraction information. |
|  | 13b | Present separate recommendations for important subgroups if the evidence suggests that there are important differences in factors influencing recommendations, particularly the balance of benefits and harms across subgroups. |
|  | 13c | Indicate the strength of recommendations and the certainty of the supporting evidence. |
| Rationale/explanation for recommendations | 14a | Describe whether values and preferences of the target population(s) were considered in the formulation of each recommendation. If yes, describe the approaches and methods used to elicit or identify these values and preferences. If values and preferences were not considered, provide an explanation. |
|  | 14b | Describe whether cost and resource implications were considered in the formulation of recommendations. If yes, describe the specific approaches and methods used (such as cost-effectiveness analysis) and summarize the results. If resource issues were not considered, provide an explanation. |

**External Review and Acknowledgement Module**

**Tool 18: Sample External Review Surveys**

The following are examples of external review surveys used to gather feedback from practitioners on an adapted guideline.

**Physician Questionnaire for the Evaluation Program of Chinese Medicine**

**Guidelines for Psoriasis vulgaris**

**Length of time in business**: **Gender**:F□ M□

**Practice setting**:Chinese medicine hospital□ General hospital□ Community hospital□ Private hospital□ Individual clinic□

**Which Chinese medicine guidelines for psoriasis vulgaris do you currently follow**:

Chinese Association of Traditional Chinese Medicine□ World Federation of Chinese Medicine□ Chinese Society of Integrated Traditional Chinese and Western Medicine□

Other□  Please indicate which:

Provincial guidelines □ Please indicate which:

Not sure□

| For each item, please check off the box that most adequately reflects your opinion. | Strongly Strongly  Agree Disagree |
| --- | --- |
| **Current use of clinical practice guidelines (CPGs)** | 1 2 3 4 5 |
| I receive TCM CPGs on psoriasis vulgaris from a variety of sources | □ □ □ □ □ |
| I receive TCM CPGs on psoriasis vulgaris that contradict one another | □ □ □ □ □ |
| Contradictory CPGs make it difficult to decide which to use | □ □ □ □ □ |
| **Panel process and consensus statement** | □ □ □ □ □ |
| The cervical cancer screening panel is credible | □ □ □ □ □ |
| The consensus statement made by the panel is reasonable | □ □ □ □ □ |
| The consensus statement may have been influenced by vested interests | □ □ □ □ □ |
| The process used by the panel to come to consensus is credible | □ □ □ □ □ |
| If I agreed with the recommendations, I would use a guideline that was developed outside of Canada | □ □ □ □ □ |
| The consensus statement is applicable to the majority of female patients in my practice | □ □ □ □ □ |
| Following this consensus statement would not require major changes to my practice | □ □ □ □ □ |
| This consensus statement is likely to be used by most of my colleagues | □ □ □ □ □ |
| This consensus statement is flexible enough to allow for clinical judgment | □ □ □ □ □ |
| If the Canadian College of Family Physicians endorsed this consensus statement, I would be more likely to follow it | □ □ □ □ □ |
| If the Canadian Strategy for Cancer Control endorsed this consensus statement, I would be more likely to follow it | □ □ □ □ □ |
| I would find it useful to have access to quality systematic appraisals of existing CPGs for topics related to family practice | □ □ □ □ □ |

**I would *accept* the consensus statement made by this expert panel:**

Absolutely□ With modifications□ I reject the consensus statement□

**I would *follow* the consensus statement made by this expert panel:**

Very likely□ Somewhat likely□ Not at all likely□

**Comments:**

All information you provide will remain CONFIDENTIAL. Results of the survey will only be presented in

aggregate form and your name will not appear on any reports.

**Practitioner Feedback Survey**

| **GUIDELINES FOR CHINESE MEDICINE DIAGNOSIS AND TREATMENT**  **OF PSORIASIS VULGARISPRACTITIONER FEEDBACK** |
| --- |

**DRAFT PRACTICE GUIDELINE REPORT #**

For each item, please check off the box that most adequately reflects your opinion.

| 1. Are you responsible for the care of patients for whom this draft guideline report is relevant? This may include the referral, diagnosis, treatment, or follow-up of  patients. | □  yes | □  no | □  unsure |
| --- | --- | --- | --- |
| If you answered “*No*” or “ *Unsure*”, please return this questionnaire to the address on the reverse side. If you answered “ *Yes*”, please answer the questions below and return to the address on the reverse side. | | | |
|  | strongly disagree  strongly agree  neither agree or disagree | | |
| 2. The rationale for developing a guideline, as stated in the “*Choice of Topic*” section of this draft report, is clear. | □ □ □ □ □ | | |
| 3. There is a need for a guideline on this topic. | □ □ □ □ □ | | |
| 4. The literature search is relevant and complete (e.g., no key trials were missed nor any included that should not have been) in this draft guideline. | □ □ □ □ □ | | |
| 5. I agree with the methodology used to summarize theevidence included in this draft guideline. | □ □ □ □ □ | | |
| 6. The results of the trials described in this draft guideline are interpreted according to my understanding of the data. | □ □ □ □ □ | | |
| 7. The draft recommendations in this report are clear. | □ □ □ □ □ | | |
| 8. I agree with the draft recommendations as stated. | □ □ □ □ □ | | |
| 9. The draft recommendations are suitable for the patients for whom they are intended. | □ □ □ □ □ | | |
| 10. The draft recommendations are too rigid to apply to individual patients. | □ □ □ □ □ | | |
| 11. When applied, the draft recommendations will produce more benefits for patients than harms. | □ □ □ □ □ | | |
| 12. The draft guideline report presents options that will be acceptable to patients. | □ □ □ □ □ | | |
| 13. To apply the draft recommendations will require reorganization of services/care in my practice setting. | □ □ □ □ □ | | |
| 14. To apply the draft recommendations will be technically challenging. | □ □ □ □ □ | | |
| 15. The draft recommendations are too expensive to apply. | □ □ □ □ □ | | |
| 16. The draft recommendations are likely to be supported by a majority of my colleagues. | □ □ □ □ □ | | |
| 17. If I follow the draft recommendations, the expected effects on patient outcomes will be obvious. | □ □ □ □ □ | | |
| 18. The draft recommendations reflect a more effective approach for improving  patient outcomes than is current usual practice. (if they are the same as current practice, please tick NA). *NA* □ | □ □ □ □ □ | | |

| 19. When applied, the draft recommendations will result in better use of resources than current usual practice (if they are the same as current practice, please tick NA). *NA* □ | □ □ □ □ □ | | |
| --- | --- | --- | --- |
| 20. I would feel comfortable if my patients received the care recommended in the draft guideline. | □ □ □ □ □ | | |
| 21. This draft report should be approved as a practice guideline. | □ □ □ □ □ | | |
|  | not at  All likely | unsure | Very  likely |
| 22. If this draft report were to be approved as a practice guideline, how likely would you be to make use of it in your own practice? | □ □ □ □ □ | | |
| 23. If this draft report were to be approved as a practice guideline, how likely would you be to apply the recommendations to your patients? | □ □ □ □ □ | | |

**COMMENTS ABOUT THE DRAFT PRACTICE GUIDELINE REPORT**

|  |
| --- |
|  |
|  |
|  |
|  |
|  |
|  |
|  |
|  |
|  |
|  |
|  |
|  |
|  |
|  |
|  |
|  |

Thank you for taking time to respond.

Please visit our Website for access to the most up-to-date versions of all completed

clinical practice guideline and evidence summary reports.

**Tool 19: Table for Reporting on Results of Update Process**

| **Health**  **question** | **Recommendation**  **in original guideline(s)** | **End date of literature**  **search** | **New**  **evidence**  **(references)** | **Final**  **recommendation** | **Comments** |
| --- | --- | --- | --- | --- | --- |
| Q 1 |  |  |  |  |  |
| Q 2 |  |  |  |  |  |
| Q 3 |  |  |  |  |  |
| Q 4 |  |  |  |  |  |
| Q 5 |  |  |  |  |  |
| Q n |  |  |  |  |  |
