# Supplementary material for: Adapting the ADAPTE framework for Traditional Chinese Medicine clinical practice guidelines: a methodological study
Source: Chin Med. 2026 Jan 20;21:42. doi: 10.1186/s13020-026-01323-1 (PMC12817658; doi:10.1186/s13020-026-01323-1)
Supplement: Supplementary file 3 — Supplementary Material 3. [file 13020_2026_1323_MOESM3_ESM.docx]

Comparison Table: Original ADAPTE vs. Initial version of ADAPTE-TCM

| **Step number** | **Original ADAPTE component** | **ADAPTE-TCM** |
| --- | --- | --- |
| Step 1 | Tool 1 and Tool 2 | Additions to websites for TCM/Integrated Chinese and Western medicine guideline sources in tool 1.and the addition of the search sources and strategies provided in Tool 2. |
| Step 2 | For the remainder of the document, the term 'panel' will refer to the multidisciplinary group convened for the tasks of the adaptation process.  Members of the organizing committee may also be panel members or may solely act to set the process in place. | Additions of references to the information provided when the guideline is formed into an adapted group: *Manual for the Development of Integrated Chinese and Western Medicine Treatment Guidelines.* |
| Step 3 | There are a number of criteria that can be used to identify and prioritize areas for best practice and guideline adaptation.  For example, these criteria might include:   - The existence of underuse, overuse, or misuse of interventions | An additional criterion has been added: characteristic advantageous diseases of traditional Chinese medicine, modifying criterion 3 to: insufficient, excessive or improper use of interventions (such as acupuncture and massage) |
| Step 4 | The following skills should be represented on the panel:   - Clinical knowledge in the topic area—knowledge of the issues related to the application of the guideline in local practice and of the latest research in the topic area. | Ensuring resources needed for guideline adaptation, suggests adding specialized skills in TCM clinical, guideline methodology and information retrieval. |
| Step 6 | - Illustration – Set Up Phase | - Demonstration of switching to TCM diagnosis and treatment of psoriasis vulgaris in the example section. |
| Step 7 | Tool 6 – PIPOH | Switching to TCM for psoriasis vulgaris raises PIPOH questions in Tool 6 |
| Step 10, | Tool 9 – AGREE Instrument | Suggesting the utilization of the Chinese Medicine Guidelines Research and Evaluation Tool (AGREE II for TCM) tool for screening of TCM guidelines. |
| Step 11 | The AGREE instrument  The appraisal of guidelines research & evaluation (AGREE) Instrument (www.agreetrust.org) provides a framework for assessing the quality of clinical practice guidelines. | - It is recommended that the quality of TCM CPGs be evaluated using the Research and Evaluation Tool for TCM CPGs (AGREE II for TCM), and it is recommended that the quality evaluation tool for TCM Evidence-based diagnosis be applied to evaluate the quality of the evidence-based diagnostic part of the TCM CPGs. - Addition of Tool 10–Quality evaluation of TCM diagnosis based on syndrome differentiation. |
| Step 12 | Assess guideline currency | When evaluating the timeliness of TCM CPGs, it is recommended that attention should be paid to the time span of evidence in the guidelines and analyze whether ancient evidence is included. |
| Step 13 | - Step 13. Assess guideline content - Matrices are tables of recommendations drawn from the guidelines under review, although they also might include recommendations from systematic reviews or health technology assessments. We recommend that a clinician who specializes in the topic produce or review the matrices to ensure that no recommendation has been taken out of context. - Tool 12-Sample Recommendations Matrices | - When conducting and evaluating the summary of guideline content, it is recommended that the summary form be filled out or reviewed by a TCM clinician who specializes in the topic. - Revise the sample in Tool 12 into a comparative summary table of clinical recommendations for the blood-heat syndrome, as outlined in *TCM diagnosis and treatment guidelines for psoriasis vulgaris* and *Integrated traditional Chinese and western medicine diagnosis and treatment guidelines for psoriasis vulgaris*. |
| Step 14 | In performing these evaluations, the panel will need to review the source guidelines thoroughly. The evaluations will help identify any recommendations in the source guidelines that do not follow directly from the evidence; panel members can then determine whether they will eliminate those recommendations from further consideration. | - When evaluating guideline consistency, it is recommended that it should be conducted by both TCM clinicians and methodologies. |
| Step 15 | Assessing whether a recommendation is acceptable and/or applicable or not is done by discussing each recommendation in light of the following questions | - When evaluating the acceptability or feasibility of the recommendations, it is suggested that a particular consideration for TCM CPGs is the acceptance of local culture and policy for TCM, Chinese medicine, and invasive procedures such as acupuncture. - It is recommended that the applicability of these guidelines be assessed using the resource of *the manual for developing integrated traditional Chinese and western medicine clinical practice guidelines*. |
| Step 16 | review assessments | Integrate the evaluation results of all TCM CPGs assessment tools mentioned in the previous steps into the review summary information. |
| Step 17 | Caution | Focus on how to transform complex interventions based on "syndrome differentiation and treatment" (such as rheumatic fever accumulation syndrome, blood deficiency and wind-dryness syndrome) into clear recommendations that healthcare practitioners in the target country or region can understand and patients can accept, while fully considering the locally available herbal products and acupuncture services. |
| Step 19 | A structured questionnaire is helpful for this step | It is recommended to refer toChapter 11 of *Manual for the Formulation of Guidelines for Combined Traditional Chinese and Western Medicine Diagnosis and Treatment.* |
| Step 20 | In order to help with widespread implementation, we recommend that the adapted guideline be formally endorsed by professional body(ies) or organization(s) most closely connected to the guideline topic (e.g., a national college of family physicians might endorse guidelines related to primary care).. The endorsement of a guideline by relevant professional organizations has been shown to enhance the acceptability of a guideline to the organization's members. | Before the guideline is released, it is recommended that it be formally endorsed by the local TCM administration or TCM societies/associations or alliances before release. |
| Step 22 | Illustration – Process of external review of the cervical cancer screening guideline | Replace the illustration with the process of external review of the Chinese medicine diagnosis and treatment of psoriasis vulgaris guideline. |
| Step 24 | The final product might be reviewed using the AGREE instrument (6) as a checklist to assess how the adapted guideline rates with respect to quality criteria. | It is recommended that the final guideline be reviewed using the AGREE for TCM instrument or RIGHT-TCM instrument. |
